# Cys-SH based quantitative redox proteomics of salt induced response in sugar beet monosomic addition line M14

**DOI:** 10.1186/s40529-021-00320-x

**Published:** 2021-10-18

**Authors:** Jinna Li, Kun Wang, Meichao Ji, Tingyue Zhang, Chao Yang, He Liu, Sixue Chen, Hongli Li, Haiying Li

**Affiliations:** 1 Ministry of Education, School of Chemistry and Materials Science, Heilongjiang University, Harbin, 150080 China; 2 Key Laboratory of Molecular Biology of Heilongjiang Province, College of Life Sciences, Heilongjiang University, Harbin, 150080 China; 3 Engineering Research Center of Agricultural Microbiology Technology, Ministry of Education, Heilongjiang University, Harbin, 150080 China; 4 Proteomics and Mass Spectrometry, Interdisciplinary Center for Biotechnology Research, University of Florida, Gainesville, FL 32610 USA; 5 Department of Biology, Genetics Institute, Plant Molecular and Cellular Biology Program, University of Florida, Gainesville, FL 32610 USA

**Keywords:** Sugar beet M14 line, Salt stress, Redox proteomics, iodoTMTRAQ, Molecular mechanisms

## Abstract

**Background:**

Salt stress is a major abiotic stress that limits plant growth, development and productivity. Studying the molecular mechanisms of salt stress tolerance may help to enhance crop productivity. Sugar beet monosomic addition line M14 exhibits tolerance to salt stress.

**Results:**

In this work, the changes in the *BvM14* proteome and redox proteome induced by salt stress were analyzed using a multiplex iodoTMTRAQ double labeling quantitative proteomics approach. A total of 80 proteins were differentially expressed under salt stress. Interestingly, A total of 48 redoxed peptides were identified for 42 potential redox-regulated proteins showed differential redox change under salt stress. A large proportion of the redox proteins were involved in photosynthesis, ROS homeostasis and other pathways. For example, ribulose bisphosphate carboxylase/oxygenase activase changed in its redox state after salt treatments. In addition, three redox proteins involved in regulation of ROS homeostasis were also changed in redox states. Transcription levels of eighteen differential proteins and redox proteins were profiled. (The proteomics data generated in this study have been submitted to the ProteomeXchange and can be accessed via username: reviewer_pxd027550@ebi.ac.uk, password: q9YNM1Pe and proteomeXchange# PXD027550.)

**Conclusions:**

The results showed involvement of protein redox modifications in *BvM14* salt stress response and revealed the short-term salt responsive mechanisms. The knowledge may inform marker-based breeding effort of sugar beet and other crops for stress resilience and high yield.

**Supplementary Information:**

The online version contains supplementary material available at 10.1186/s40529-021-00320-x.

## Background

Salinity is a global challenge to plant growth, agriculture and world food security (Yu et al. 2016; Hsu et al. 2009; Chang et al. 2012). When plants are subjected to salt stress, it can induce osmotic stress, ionic stress, oxidative stress and other secondary stress (Khan et al. 2007; Yang et al. 2018). Plants respond and adapt to adverse environments through a variety of physiological, biochemical and molecular processes (Howat et al. 2000; Xu et al. 2019). The protein stability, catalytic activity and interaction with other molecules were affected the posttranslational modifications of amino acid residues. Redox plays a multifaced role in regulates signaling, metabolic and developmental activities (Mock et al. 2016). One redox chemistry involves reversible oxidation/reduction of the sulfhydryl groups of protein cysteine residues (Cys-SH) that directly influence protein structures and functions (Heppner et al. 2018). Cysteine thiols can be oxidized in a variety of reactions (Baez et al. 2015). The redox posttranslational modifications (PTMs) include disulfide formation (S–S), S-glutathionylation (SSG), S-nitrosylation (SNO), S-sulfenylation (SOH), and S-sulfhydration (SSH), all of these can be reduced to free thiols by cellular antioxidant systems (Ji et al. 2017; Claiborne et al. 2005; Poole et al. 2004; Gupta et al. 2013; Heppner et al. 2017). Reactive oxygen species (ROS) are generated in the course of salt stress. Two ROS scavenging systems are mainly responsible for alleviation of salt stress-induced oxidative stress, i.e., enzymatic antioxidant system (e.g., glutathione S-transferase (GST), glutaredoxin (GR), superoxide dismutase (SOD) and catalase (CAT)) and non-enzymatic antioxidant system (e.g., ascorbate (AsA) and glutathione (GSH)) (Dave et al. 2012; Farooq et al. 2016; Jung et al. 2019). Experimental and bioinformatic analyses of the cysteine redoxome have been conducted to identify cellular redox active cysteines and reveal the redox networks that include ROS generation, specific types of ROS, redox sensitive proteins, GSH-linked enzymes, and biological impact (Thamsen et al. 2011; Kemp et al. 2008; Kitajima 2008; Kitajima et al. 2008). However, redox proteomic research in sugar beet response to salt stress is yet to be conducted.

Several salt stress proteomic studies in sugar beet have been reported. Wakeel A et al. identified nine proteins from sugar beet shoots and roots that changed significantly in abundance under salt stress (Wakeel et al. 2011). Sugar beet monosomic addition line M14 (hereafter named *BvM14*) was produced by crossing *Beta vulgaris* L. and *B. corolliflora* Zoss. It retains chromosome 9 of *B. corolliflora* Zoss in addition to the *B. vulgaris* L. genome (Li et al. 2009). Our previous studies have demonstrated that the *BvM14* plants can growth for seven days under 500 mM NaCl, which caused significant abundance changes to 67 unique proteins (Yang et al. 2012). Quantitative proteomics of the *BvM14* under lower salt concentrations (200 and 400 mM NaCl) revealed 75 differentially changed proteins in leaves and 43 differential proteins in roots (Yang et al. 2013). The data showed that enhancement of photosynthesis and energy metabolism, accumulation of osmolyte and antioxidant enzymes, and regulation of methionine metabolism and ion uptake/exclusion were key processed underlying the salt stress responses. Furthermore, they compared gene transcription data with the corresponding protein data (Yang et al. 2013). Later, Li et al. analyzed the changes in *BvM14* membrane proteome under salt stress (Li et al. 2015). In total, 50 proteins exhibited differential changes among the 274 identified membrane proteins. The proteins were mainly involved in transport, metabolism, protein synthesis, photosynthesis, protein folding and degradation, signal transduction, stress and defense, energy, and cell structure (Li et al. 2015). Clearly, the membrane proteomic research complemented previous work on the soluble proteins. To explore potential PTMs during the salt stress, Yu et al. studied the *BvM14* proteome and phosphoproteome under salt stress, they identified 189 phosphoproteins and 2182 unique proteins (Yu et al. 2016). This study highlighted specific kinase signaling mechanisms underlying the *BvM14* response to salt stress. Interestingly, under 200 mM NaCl condition, proteins important for redox regulations such as GR and peroxiredoxin (PrxR) were both increased at the phosphorylation level (Yu et al. 2016). GR (also known as thioltransferase) can reduce glutathionylated proteins, and PrxR uses a similar thiol-based mechanism to reduce H_2_O_2_ (Yu et al. 2020).

Oxidative stress and redox regulation appear to be important processes of sugar beet salt stress response (Yang et al. 2013; Yu et al. 2016). To understand how redox regulation plays a role in the response of *BvM14* to salt stress, it is important to profile redox PTMs that occur to redox sensitive proteins. Most of the redox proteomics experiments showed changes in the protein thiol redox state do not address changes in the overall protein turnover. To overcome this potential complication, a double-labeling strategy iodoTMTRAQ was developed to integrate iodoacetyl (iodo)TMT reagents for profiling redox PTMs with the isobaric Tags for Relative and Absolute Quantitation (iTRAQ) reagents designed for quantifying total protein level changes (Parker et al. 2015). In this study, we apply the iodoTMTRAQ strategy to identify and quantify redox proteome and total proteome changes in *BvM14* line under short-term salt stress. The data have revealed new redox responsive proteins and their potential roles in the response to salt stress. The results have improved our understanding of redox responsive proteins in plants salt stress response.

## Materials and methods

### Plant materials and NaCl treatment

The sugar beet M14 seeds were sterilized with 70% (v/v) ethanol, 0.1% (w/w) mercurial chloride and 0.2% (w/w) thiram, and then sown in vermiculite for germination. After one week, the seedlings were transferred to hydroponic medium of the Hoagland solution (Ghoulam et al. 2002). Seedlings were grown in a growth chamber under a 13 h light/11 h dark cycle, 25/20°Cday/night temperature, 450 μmol m^−2^ s^−1^ light intensity and a relative humidity of 70%. Three-week-old seedlings were divided into two groups: (1) control group (without NaCl); (2) treatment group (200 mM and 400 mM NaCl for 5, 10, 20, 30, 60, 90 min). The NaCl concentrations were chosen according to a previous report showing that the M14 line can tolerate up to 500 mM NaCl (Yang et al. 2012). Leaves of control and treated M14 seedlings were harvested directly into liquid nitrogen and stored in − 80 °C. At least three independent biological replicates of control and treated samples were analyzed in all the experiments.

### Ascorbic acid (AsA) and glutathione (GSH) content assay

For ascorbic acid (AsA) and glutathione (GSH) content assays, 0.1 g leaf material was ground in 1 mL reagent from either the ascorbic acid assay kit (AsA-1-W) or the glutathione assay kit (GSH-1-W) from Comin Inc (Harbin, China). After centrifugation at 8000 rpm, 4 °C for 20 min, the supernatant was used for AsA and GSH content assays according to manufacturer instructions. Three independent biological replicates were prepared for each sample.

### Protein extraction and blockage of free thiols

Protein extraction from the *BvM14* leaves was performed according to a phenol extraction method (Ghoulam et al. 2002). Briefly, 2 g M14 leaves were ground into a fine powder in liquid nitrogen and suspended in 1.25 mL Tris saturated phenol (pH8.8) and 1.25 mL phenol extraction buffer (900 mM sucrose, 100 mM Tris–HCl (pH8.8), 1 mM PMSF, 20 mM N-ethylmaleimide (NEM), 10 mM EDTA) (Parker et al. 2015; Yuan et al. 2019). NEM will irreversibly block free cysteine thiols during the protein extraction process. Protein samples were prepared from three independent biological replicates, and protein concentration was determined using a 2D Quant kit (GE Healthcare, USA) with BSA (2 mg/mL) as the standard (Parker et al. 2012).

### iodoTMT labeling and trypsin digestion

Reduced thiols for reverse labeling were generated by incubating the protein samples with 5 mM tris (2-carboxyethyl) phosphine for 1 h at 50 °C. We labeled 0, 30 and 60 min control samples with 126, 128 and 130 iodoTMT reagents, and the salt treated samples with 127, 129 and 131 reagents, respectively. Labeling was performed at 37 °C for 2 h in the dark, then quenched with 0.5 M DTT for 15 min at 37℃ in the dark. Trypsin (Sequencing grade, Promega, Madison) was added with an enzyme to protein ratio of 1:50 (w/w) and the digestion was performed at 37 °C overnight (Parker et al. 2012). Peptides were cleaned up with C18 desalting columns (The Nest Group Inc., Southborough, MA) and lyophilized to dryness.

### iTRAQ labeling, strong cation exchange fraction and LC–MS/MS

The C18 cleaned peptides were labeled with iTRAQ reagents according to the manufacturer’s protocol (AB Sciex Inc., Framingham, MA, USA). The 0, 30 and 60 min control samples were labeled with reporter tags 113, 115 and 117, and the treatment samples were labeled with reporter tags 114, 116 and 118, respectively. The labeling was conducted at 37℃ for 2 h, and the labeled peptides were desalted according to a previous procedure (Yu et al. 2016; Parker et al. 2012). LC–MS/MS was carried on an Easy-nLC 1000 connected to a Q-Exactive Plus MS/MS system (Thermo Fisher Scientific, Bremen, Germany). The peptides were loaded onto an Acclaim Pepmap 100 pre-column and separated on a PepMap RSLC analytical column, followed by tandem mass spectrometry according to the method of Yu et al. (Yu et al. 2016).

### Data analysis

The MS/MS data were searched against the *B. vulgaris* database (52,749 entries) using Proteome Discoverer 2.1 (Thermo Fisher Scientific, Bremen, Germany) with the parameters from a previous publication (Yin et al. 2017). We used iodoTMT and iTRAQ reporter ion peak intensities for relative quantification with unique peptides. Each iodoTMT tag was exported as unique peptide peak intensities, and ratios were calculated accordingly peak intensity values. We used student’s *t*-test conducted between the fold change of iodoTMT labeled peptides and the fold change of the corresponding proteins based on iTRAQ. The protein should be quantified in all the three biological replicates. The protein fold change > 1.2 or < 0.8 (p-value < 0.05) were used to determine significant redox or total protein level changes. All the proteins were searched by NCBInr and Uniprot (http://www.ebi.uniprot.org) for functional annotation, subcellular location and gene ID numbers of the homologous proteins. Gene ontology (GO) (http://geneontology.org) terms and imported Kyoto Encyclopedia of Genes and Genomes (KEGG) (https://www.kegg.jp) database were used for Blast2GO analysis (Conesa et al. 2005). The functional enrichment analysis was performed according to Yu’s procedure (Yu et al. 2016).

### Quantitative Real time PCR analysis

Total RNA was isolated from frozen samples using a TRIZOL reagent (Invitrogen). By adding DNase I, genomic DNA was removed and cDNA was synthesized using the PrimeScript™ RT Master Mix (Perfect Real Time) (TakaRa, Shiga, Japan). Gene specific primers of the target genes were designed using online Primer3 Plus according to a previous report (Untergasser et al. 2007). Quantitative RT-PCR analysis was performed in a 30 µL volume containing 15 µL of Power UpTM SYBRTM green master (Applied Biosystems, Vernon, CA, USA), 3 µL of 20-fold diluted cDNA, 3 µL of each gene-specific primer, and 9 µL of ddH_2_O. The PCR conditions were as follows: 95 °C for 3 min; 95 °C for 15 s, 59 °C for 30 s, 40 cycles. Three biological replicates were used for each sample. Reaction was conducted on an ABI7500 (Applied Biosystems, Vernon, CA, USA). All the data were analyzed using ABI7500 software (Applied Biosystems, Vernon, CA, USA) and Graphpad Prism 6.01. The comparative CT method (2^−ΔΔCT^) was used for relative quantification of gene transcripts. Each biological sample comprised of three technical repeats and each experiment was repeated three times (Pichon et al. 2017).

## Results

### Changes of AsA and GSH in *BvM14* leaves under salt stress

**Fig.1:**
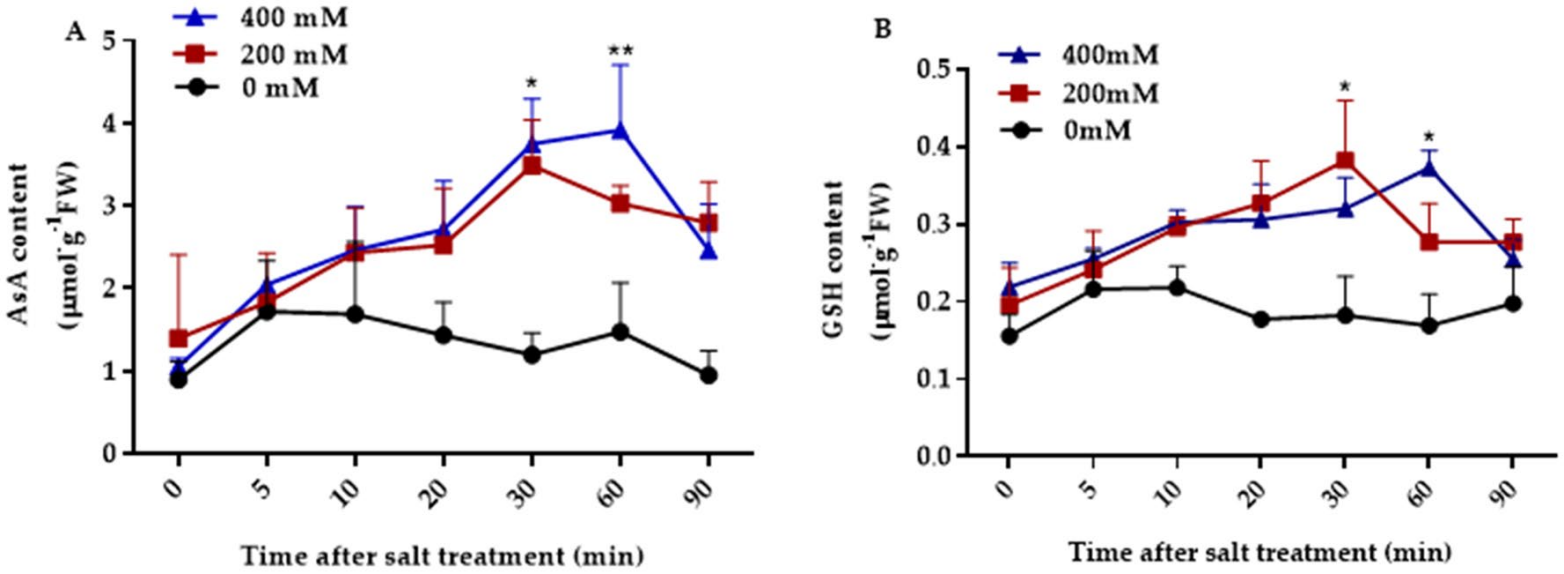
Temporal changes of AsA and GSH contents in leaves of *BvM14* plants after salt stress treatments. **A** AsA contents under 200 mM and 400 mM NaCl stress. **B** GSH contents under 200 mM and 400 mM NaCl stress. The values are the mean of three biological replicates from different samples with standard errors

Recent studies showed that AsA and GSH are major antioxidants in plant salt stress response (Navrot et al. 2011; Lin et al. 2020; Khan et al. 2020), here we measured changes of two major antioxidants AsA and GSH at 0, 5, 10, 20, 30, 60 and 90 min after 0, 200, 400 mM NaCl treatments. As shown in Fig. 1, under control conditions, the contents of AsA and GSH in *BvM14* leaves maintained at fairly constant levels during the 90 min of assay time. Compared to control conditions, both the AsA and GSH contents reached maximum after 30 min of 200 mM NaCl stress. While after 60 min of 400 mM NaCl stress, both the AsA and GSH contents reached the peak level (Fig. 1). The results clearly showed that salt stress caused significant cellular redox changes as early as 10 min after the treatment. Based on the AsA and GSH changes, we selected samples collected at 30 min and 60 min of 200 mM and 400 mM NaCl conditions, respectively, for iodoTMTRAQ-based redox proteomics.

### Identification of differential proteins and different redox proteins in response to salt stress

**Fig.2:**
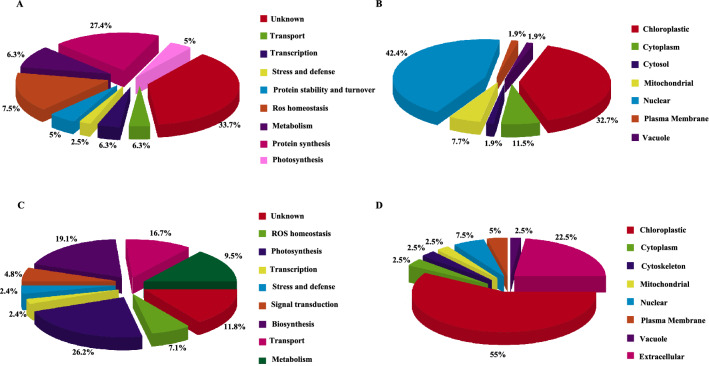
Functional classification and subcellular location of the differential redox proteins and differential proteins. **A** Functional classification of 80 differential proteins; **B** Subcellular location of 80 differential proteins; **C** Functional classification of 42 differential redox proteins; **D** Subcellular location of 42 differential redox proteins

Using iodoTMTRAQ LC–MS/MS and database searching, a total of 1290 proteins were identified in *BvM14* leaves (Additional file 3: Table S1). Eighty proteins were differentially changed in abundance (based on iTRAQ reporter fold change > 1.2, or < 0.8, p < 0.05) in salt-treated samples compared to the control samples (Additional file 4: Table S2). Only four differential proteins were identified under the 200 mM NaCl treatment, while 77 were identified under the 400 mM NaCl treatment. Functional classification of the differential proteins revealed the following distribution: metabolism (6.3%), protein synthesis (27.4%), transport (6.3%), stress and defense (2.5%), ROS homeostasis (7.5%), protein stability and turnover (5%), photosynthesis (5%), transcription related (6.3%) and unknown (33.7%) (Fig. 2A). The subcellular locations of the 80 differential proteins were classified to the chloroplast (32.7%), cytoplasm (11.5%), cytosol (1.9%), mitochondrial (7.7%), nuclear (42.4%), plasma membrane (1.9%) and vacuole (1.9%) (Fig. 2B). Biological process, molecular functional and cell components were significantly enriched by AgriGO (Additional file 2: Fig. S2). And among these biological processes, the interesting terms included response to stimulus, response to stress, and so on, whereas the interesting molecular functions such as RNA binding, structural molecule activity.

Based on iodoTMT reporter intensities, we identified 42 proteins with significant redox changes in response to salt stress (Additional file 5: Table S3). Here are the functional categories of the differential redox proteins: metabolism (9.5%), transport (16.7%), biosynthesis (19.1%), transcription related (2.4%), signal transduction (4.8%), stress and defense (2.4%), ROS homeostasis (7.1%), photosynthesis (26.2%) and unknown (11.8%) (Fig. 2C). The subcellular localizations of the redox proteins were classified to the chloroplast (55%), cytoplasm (2.5%), cytoskeleton (2.5%), mitochondrial (2.5%), nuclear (7.5%), plasma membrane (5%), extracellular (22.5%) and vacuole (2.5%) (Fig. 2D). Biological process, molecular functional and cell components were significantly enriched by AgriGO (Additional file 1: Fig. S1). And among these biological processes, the interesting terms included response to stimulus, cellular process and so on, the molecular functions such as RNA binding, hydrolase activity. Among the 42 differential redox proteins, four were identified under 200 mM NaCl treatment, and 40 were identified under 400 mM NaCl treatment. There were 31 oxidized and 18 reduced cysteine residues in the redox proteins (Tables 1, 2).

**Table 1:** List of 28 up-regulated redox proteins from sugar beet M14 leaves between control and NaCl treatment (all the entries with p < 0.05 from three biological replicates)

No	Protein ID^a^	Description	Plant species	Sequence with modification^b^	iodoTMTsalt200 /control Ratio^c^	iodoTMTsalt400 /control Ratio^d^	*p*-value	iTRAQ salt200 /control Ratio^f^	iTRAQ salt400 /control Ratio^g^	Function^e^
1	G1E6K5	Carbonic anhydrase	*Dimocarpus longan*	FMVFAC^210^SDSR	–	1.40	0.01	–	1.09	Metabolism (3)
2	A0A0K9RGC9	Beta-galactosidase	*Spinacia oleracea*	YWPTFGPQC^653^NLYVPAPLLR	–	1.20	0.03	–	1.01	
3	A0A2P6UZB2	Triosephosphate chloroplastic	*Micractinium conductrix*	VIAC^1073^VGETLEQR	–	1.40	0.01	–	0.93	
4	Q8MC96	ATP synthase epsilon chain	*Apium graveolens*	TLNLC^5^VLTPNR	2.40	–	0.00	–	0.81	Transport (3)
5	A0A1S2XUR4	GDSL esterase/lipase At5g33370	*Cicer arietinum*	VLVTGTGPLGC^230^VPGELASQ GSQNGEC^245^APEPQR		1.66	0.01	–	0.31	
6	A0A1U7VX65	GDSL esterase/lipase At5g45670-like	*Nicotiana sylvestris*	FALIGIGQIGC^221^SPNQLAQRSP DGATC^239^DDTVNSANR	–	1.52	0.01	–	0.31	
7	C0Z387	AT2G21660 protein	*Arabidopsis thaliana*	C^10^FVGGLAWATDDR	–	1.48	0.01	–	1.07	Biosynthesis (5)
8	A0A0K9QZ36	Biotin carboxyl carrier protein of acetyl-CoA carboxylase	*Spinacia oleracea*	QYDC^147^ELLIR	–	1.21	0.01	–	1.11	
9	A0A1J3HHY8	Glutamate-1-semialdehyde 2,1-aminomutase	*Noccaea caerulescens*	FVNSGTEAC^200^MGVIR	–	1.57	0.04	–	1.18	
10	O24365	Chloroplast mRNA-binding protein CSP41	*Spinacia oleracea*	LC^325^AQATGR	–	1.94	0.01	–	0.95	
11	O50036	Heat shock 70 protein	*Spinacia oleracea*	FEELC^377^SDLLDR	*–*	1.31	0.00	*–*	1.12	
12	A0A1D1Y6P4	Vacuolar-sorting receptor 2	*Anthurium amnicola*	YC^286^APDPEQDFSR	*–*	1.22	0.02	*–*	1.08	Signal transduction (2)
13	A0A1R3GZ43	EGF-like calcium-binding protein	*Corchorus olitorius*	YC^269^APDPEQDFSR	–	1.22	0.01	–	1.00	
14	A0A161DY72	DUF642	*Vitis quinquangularis*	SDDFSSLC^352^GPVIDDVR; VAEIMIHNPGVEEDPAC^173^GPLIDSVAMR	–	1.37	0.04	–	0.80	Stress and defense (1)
15	A0A0K9QD73	Profilin	*Spinacia oleracea*	TGQALVIGLYDEPVTPGQC^117^NMIVER	–	1.32	0.02	–	0.77	Transcription (1)
16	P10871	Ribulose bisphosphate carboxylase/oxygenase activase	*Spinacia oleracea*	MC^223^ALFINDLDAGAGR	1.51	1.48	0.03	1.09	1.07	Photosynthesis (7)
17	A0A0K9QU20	Fructose-bisphosphate aldolase	*Spinacia oleracea*	TVVSVPC^197^GPSALAVKEAAWGLAR	–	1.20	0.00	0.94	0.92	
18	A0A1U8EH95	Ribulose bisphosphate carboxylase/oxygenase activase 2	*Capsicum annuum*	KGNMC^470^VLFINDLDAGAGR	–	1.78	0.03	–	1.05	
19	O20252	Sedoheptulose-1,7-bisphosphatase	*–*	LFC^255^PGNLR	–	1.47	0.04	–	0.87	
20	P09559	Phosphoribulokinase	*Spinacia oleracea*	FFNPVYLDEGSTISWIPC^296^GR	*–*	1.33	0.04	–	1.08	
21	P12355	Photosystem I reaction center subunit III	*Spinacia oleracea*	FENYGNYGLLC^139^GSDGLPHLIVSGDQR	*–*	1.20	0.01	*–*	0.80	
22	P10871	Ribulose bisphosphate carboxylase/oxygenase activase	*Spinacia oleracea*	IGVC^316^TGIFR	*–*	1.26	0.00	*–*	1.00	
23	A0A0K9QDU1	Peroxidase	*Spinacia oleracea*	NSFYASTC^31^PGVEGIVR	–	1.49	0.03	*–*	0.67	ROS homeostasis (2)
24	A0A1J6IUE1	Thioredoxin-like 3–1	*Nicotiana attenuata*	ENSQPIIIDWMANWC^108^R	–	1.43	0.01	*–*	1.06	
25	A0A0K9R8D4	Uncharacterized protein LOC104907026	*–*	AGQFC^117^GGFTAIER	0.38	1.47	0.00	1.01	0.95	Unknown (4)
26	A0A068TKJ7	Uncharacterized protein	*–*	YTEGFSGADITEIC^478^QR	–	1.45	0.02	–	0.76	
27	A0A0J8CV41	Chalcone-flavonone isomerase family protein	*Beta vulgaris subsp. vulgaris*	TLPEEILNSIIGETGVC^199^PQAR	–	1.45	0.02	–	1.14	
28	A0A0K9R8D4	Uncharacterized protein	*–*	AGQFC^117^GGFTAIER	–	1.47	0.00	–	0.98	

^a^Protein ID, gi number of NCBI

^b^ Sequence with modification, the lower case letter are phosphorylation site in each peptide

^c^salt200/control Ratio, a relative abundance of proteins at redox peptide level ( 200 mM NaCl treatment versus control), P-value < 0.05

^d^salt400/control Ratio, a relative abundance of proteins at redox peptide level ( 400 mM NaCl treatment versus control), P-value < 0.05

^e^Function, according to Blast2GO software

^f^salt200/control Ratio, a relative abundance of proteins at total protein level ( 200 mM NaCl treatment versus control), P-value < 0.05

^g^salt400/control Ratio, a relative abundance of proteins at total protein level ( 400 mM NaCl treatment versus control), P-value < 0.05. The number in brackets, indicate the numbers of proteins in corresponding function

**Table 2:** List of 15 down-regulated redox proteins from sugar beet M14 leaves between control and NaCl treatment (all the entries with p < 0.05 from three biological replicates)

No	Protein ID^a^	Description	Plant species	Sequence with modification^b^	iodoTMTsalt200 /control Ratio^c^	iodoTMTsalt400 /control Ratio^d^	*p*-value	iTRAQ salt200 /control Ratio^f^	iTRAQ salt400 /control Ratio^g^	Function^e^
1	A0A1S3CE63	Cysteine proteinase RD19a-like	*Cucumis melo*	LVSLSEQQLVDC^137^DHEC^141^DPEER	*–*	0.71	0.01	*–*	1.12	Metabolism (1)
2	A0A0K9RNM7	Non-specific lipid-transfer protein	*Spinacia oleracea*	C^100^GVSIPGPVGPQADC^114^SQIH	*–*	0.62	0.01	*–*	0.81	Transport (4)
3	P81760	Thylakoid lumenal 17.4 kDa protein	*Arabidopsis thaliana*	LPPLSTEPNRC^92^ER	*–*	0.80	0.03	*–*	0.74	
4	A0A1U7ZGK1	Mitochondrial import inner membrane translocase subunit TIM8	*Nelumbo nucifera*	FSSSEATC^55^LNNCAQR	*–*	0.60	0.02	*–*	1.05	
5	A0A1U8LRP7	Thylakoid lumenal 17.4 kDa protein	*Gossypium hirsutum*	LPPLSTEPNRC^191^ER	*–*	0.80	0.03	*–*	1.07	
6	A0A314V1F4	Extracellular ribonuclease LE-like	*Prunus yedoensis var. nudiflora*	NAIEGGVGFTPAIGC^182^NVDPAGTTQLYR ISFC^198^VDNTASNLIEC^209^PR	*–*	0.07	0.00 0.00	*–*	0.51 0.60	Biosynthesis (3)
7	A0A0K9S0G4	Peptidylprolyl isomerase	*Spinacia oleracea*	IEYYATTAEPSC^100^ELNVVR SGLAYC^112^DLVVGSGVPAPYNTLINVHYTAR	*–*	0.70	0.02 0.03	*–*	1.08 1.08	
8	B0M184	Chloroplast RNA binding protein	*Mesembryanthemum crystallinum*	EFEPTC^216^R	*–*	0.65	0.02	*–*	1.13	
9	A0A061F296	Rubredoxin-like superfamily protein	*Theobroma cacao*	FAVLNTGIYEC^118^R	*–*	0.71	0.02	*–*	0.63	Photosynthesis (5)
10	D7KW69	Ferredoxin	*Arabidopsis lyrata subsp. lyrata*	FITPEGEQEVEC^70^DDDVYVLDAAEEAGIDLPYSC^91^R	*–*	0.09	0.00	*–*	0.90	
11	A0A1U7XAS5	NADH dehydrogenase [ubiquinone] 1 beta subcomplex subunit 7-like	*Nicotiana sylvestris*	C^61^EYELVMER	*–*	0.75	0.00	*–*	1.00	
12	E5GBR8	Rubredoxin	*Cucumis melo subsp. melo*	FAVLNTGIYEC^238^R	*–*	0.80	0.02	*–*	0.77	
13	O24360	calvin cycle protein CP12	*Spinacia oleracea*	KEAQETC^69^SDDPVSSEC^78^VAAWDVVEEVSAAASHAR	*–*	0.77	0.03	*–*	0.97	
14	Q9M0C2	Putative EG45-like domain containing protein 1	*Arabidopsis thaliana*	VTC^95^VSGTNQGVPQPC^107^R	0.64	*–*	0.01	0.87	–*–*	ROS homeostasis (1)
15	A0A0K9R8D4	Uncharacterized protein	*–*	AGQFC^117^GGFTAIER	0.38	1.47	0.00	0.55	0.69	Unknown (1)

^a^Protein ID, gi number of NCBI

^b^ Sequence with modification, the lower case letter are phosphorylation site in each peptide

^c^salt200/control Ratio, a relative abundance of proteins at redox peptide level ( 200 mM NaCl treatment versus control), P-value < 0.05

^d^salt400/control Ratio, a relative abundance of proteins at redox peptide level ( 400 mM NaCl treatment versus control), P-value < 0.05

^e^Function, according to Blast2GO software

^f^salt200/control Ratio, a relative abundance of proteins at total protein level ( 200 mM NaCl treatment versus control), P-value < 0.05

^g^salt400/control Ratio, a relative abundance of proteins at total protein level ( 400 mM NaCl treatment versus control), P-value < 0.05. The number in brackets, indicate the numbers of proteins in corresponding function

### Mapping redox responsive cysteine residues in the *BvM14* response to salt stress

**Fig.3:**
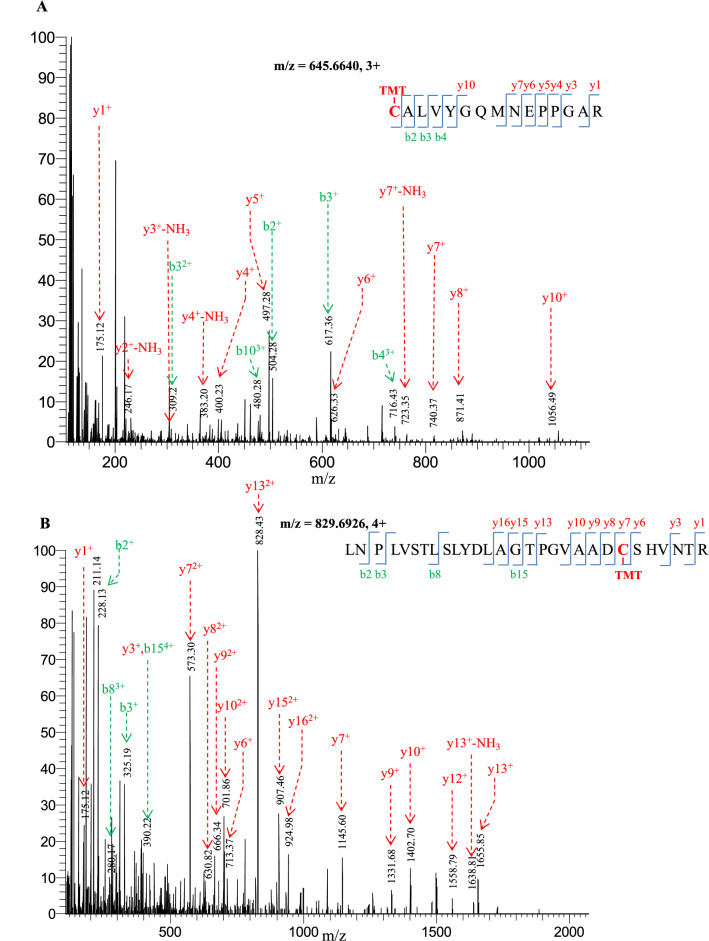
Example MS/MS spectra showing redox modified cysteine sites. **A** MS/MS spectrum of C^−TMT^ALVYGQMNEPPGAR derived from an ATP synthase **B** MS/MS spectrum of LNPLVSTLSLYDLAGTPGVAADC^−TMT^SHVNTR derived from a malate dehydrogenase

With the acquired MS/MS spectra, a total of 48 redox responsive peptides were identified in the 42 redox proteins (Additional file 5: Tables S3). In these peptides, the redox modified cysteine residues could be mapped. In Fig. 3, the MS/MS spectra of two redox peptides derived from ATP synthase (731,341,013) and malate dehydrogenase (731,329,081) were shown as examples (Fig. 3A, B).

### Transcriptional analysis of differential redox proteins and differential proteins

**Fig.4:**
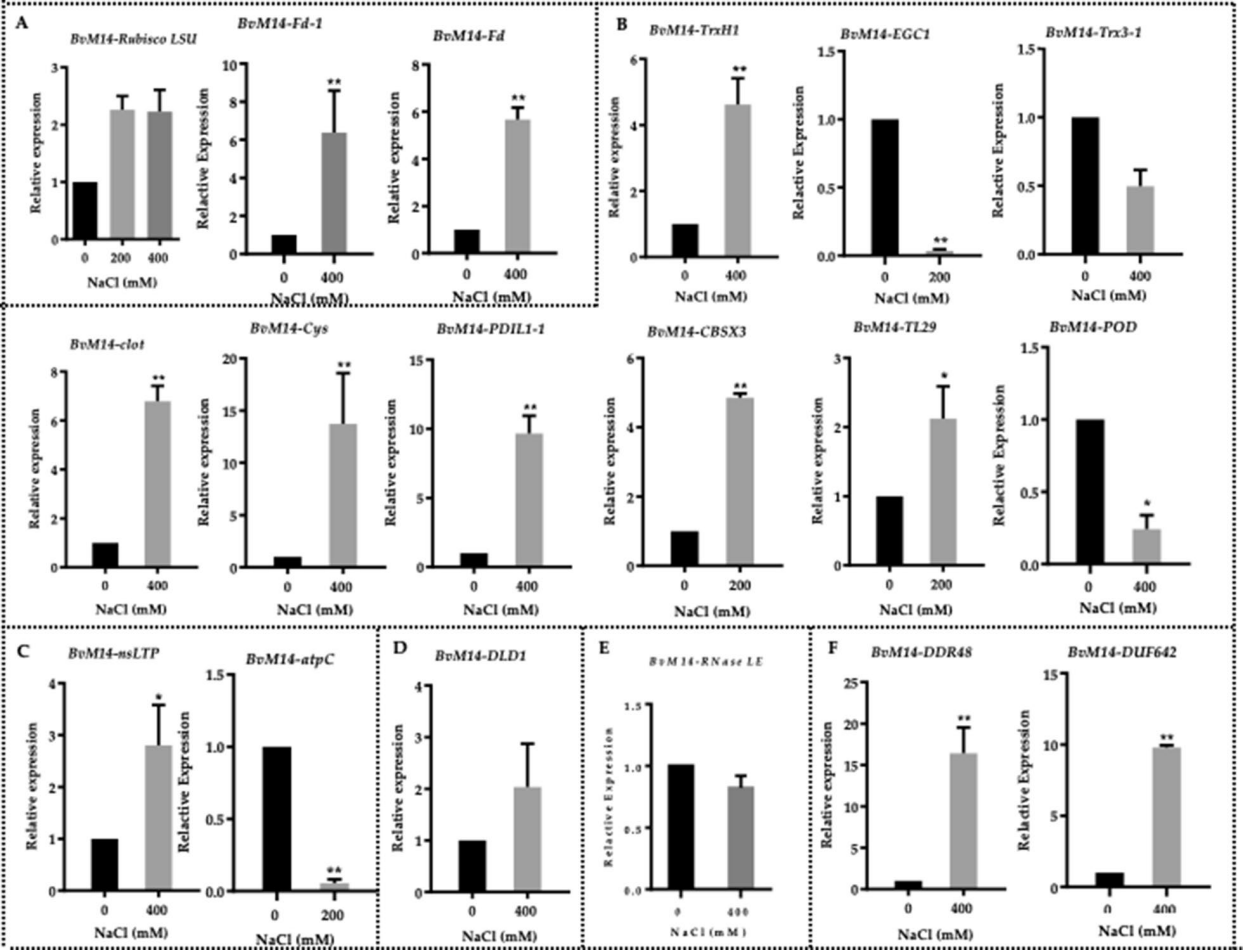
Real-time PCR analysis of the genes encoding the differential redox proteins and differential proteins in different pathways. **A** Photosynthesis, **B** ROS homeostasis, **C** Transport, **D** Metabolism, **E** Biosynthesis, and **F** Stress and defense. The x-axis is salt concentration. The y-axis is the relative expression of each gene (2^−ΔΔCT^). Asterisks represent significant differences (*p < 0.05, **p < 0.001) determined by one-way ANOVA followed by Tukey’s HSD comparisons

To test how transcriptional level changes correlate with protein level and redox protein level, 11 differential proteins and seven differential redox protein were selected for analysis of their gene transcriptional level changes. The Real-time PCR primer sequences can be found in Additional file 6: Table S4. We categorized the transcriptional expression patterns of these genes into six groups based on their functions (Fig. 4, Additional file 6: Table S4). The first group proteins were involved in photosynthesis, including Rubisco LSU, Fd, Fd-1. The second group proteins were involved in ROS homeostasis, including Clot, Cys, PDIL1-1, CBSX3, EGC1, peroxidase (POD), Trx3-1, TrxH1, and TL29. The third group belonged to transport-related pathway including nsLTP, atpC protein. The fourth group DLD1 proteins belonged to metabolism. The fifth group RNase LE proteins belonged to biosynthesis. The last group proteins were stress and defense cascade, including DDR48 and DUF642.

Among the 18 genes encoding for the differential proteins, the transcriptional levels of 12 genes were coincide with the corresponding redox level trends and total protein level trends (Additional file 7: Table S5). The transcriptional levels of ATP synthase epsilon chain (*atpC*), ferredoxin (*Fd-1*), *POD* and thioredoxin-like 3–1 (*Trx3-1*) showed different trends with the corresponding redox changes, while the extracellular ribonuclease LE-like (*RNase LE*), *DUF642* and EG45-like domain containing protein 1(*EGC1*) showed the same trend at both the protein level and transcriptional level (Additional file 7: Table S5).

### A review of potential salt stress response mechanisms in *BvM14*

**Fig.5:**
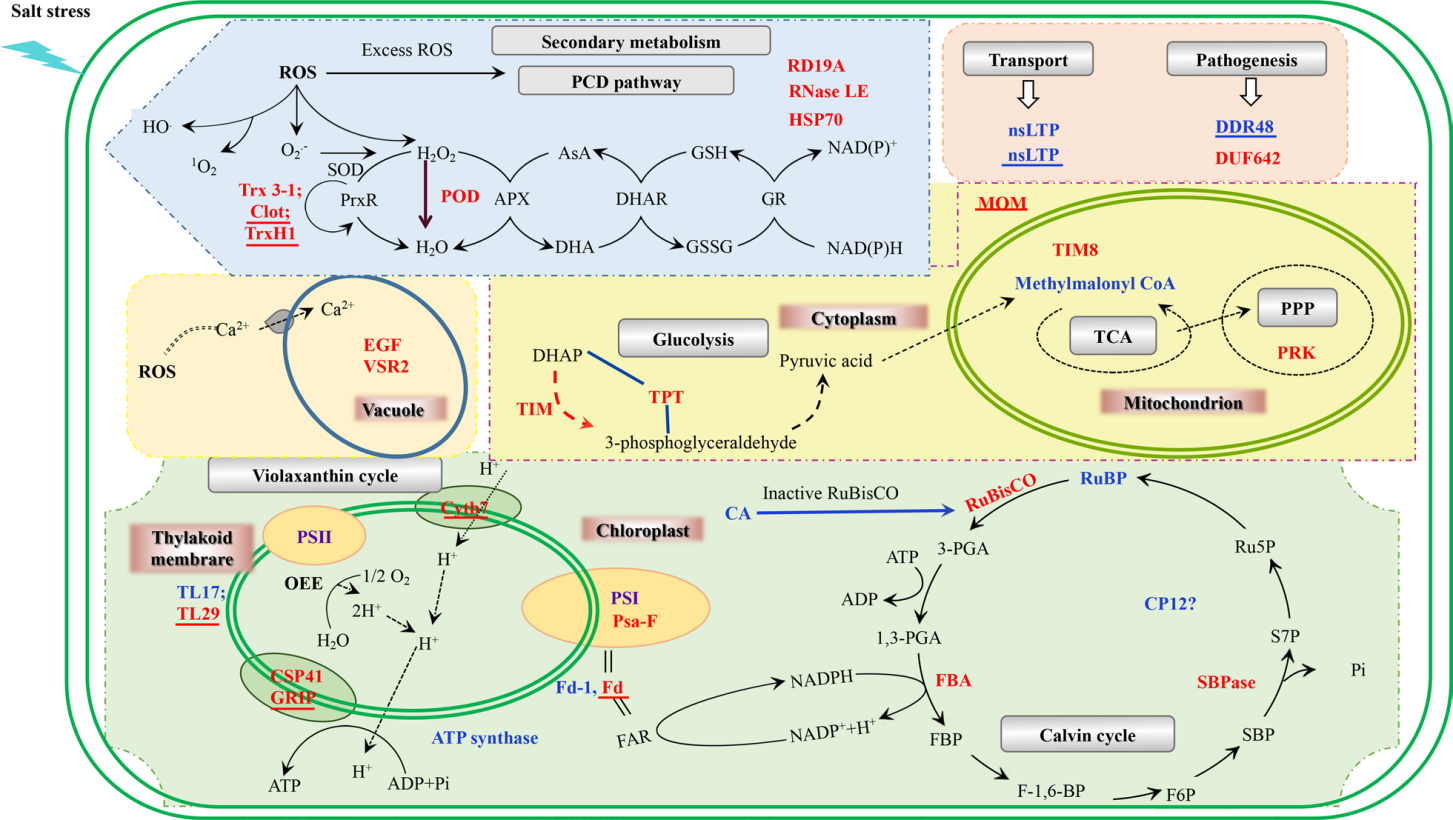
Schematic presentation of systematic salt tolerance mechanisms in *BvM14* leaves. The identified proteins and redox-responsive proteins were put into subcellular locations and KEGG pathways. The red and blue colors indicate increased and decreased in redox PTMs, respectively, and those underlined red and blue highlight proteins with increased and decreased levels, respectively. Please refer to Additional file 8: Table S6 for protein IDs

On the basis of the aforementioned results, we proposed a potential mechanism in the s *BvM14* response to short-term salt stress (Fig. 5, Additional file 8: Table S6). The differential redox proteins and total proteins put into context of subcellular locations and pathways under salt stress. The key pathways in Fig. 5 include ROS homeostasis, photosynthesis, stress and defense, transport related processes. Nevertheless, our results highlight the following potential mechanisms under salt stress: Salt stress leads to ROS production and oxidative stress, which lead to redox changes in microenvironment of cytoplasm and various organelles, resulting in redox PTMs of proteins in biochemical pathways dominated by photosynthesis and ROS homeostasis. The redox PTMs revealed in this study may play important regulatory roles in the *BvM14* salt stress response and contribute to the development of salt stress tolerance.

## Discussion

Previous work has shown that *BvM14* grew slowly and the leaves showed slightly chlorotic under 200 mM and 400 mM NaCl treatment. Obviously, the growth phenotype *of BvM14* under 400 mM NaCl treatment was suppressed (Yang et al. 2012, 2013). In this study, we have successfully applied the iodoTMTRAQ technology and identified many interesting redox-responsive proteins in the processes of metabolism, transport, biosynthesis, transcription related, signal transduction, photosynthesis, stress and defense and ROS homeostasis. The iodoTMT signal from treated samples compared to control increased that indicate oxidation of sensitive cysteines. In the discussion sections, we focus on discussing total protein and redox protein changes that are important for understanding the *BvM14* salt stress response mechanisms.

### ROS homeostasis and protein redox PTMs in *BvM14* response to salt stress

In the *BvM14* leaves, three and six ROS homeostasis proteins were identified in redox proteomics and total proteomics, respectively (Tables 1, 2; Additional file 4: Table S2). For example, peroxidase (POD) and thioredoxin-like 3–1 (Trx3-1) increased in oxidation under 400 mM NaCl treatment. Other thioredoxins, such as Trx Clot (Clot) and TrxH1 increased at total protein level under the 400 mM NaCl treatment. Trxs are important players in the antioxidant defense system by inhibiting oxidative stress induced protein oxidation, which can also be triggered by other environmental stress factors (Miller et al. 2010). They modulate the target proteins’ function by oxidoreductase activities (Meyer et al. 2012) and play critical regulatory roles in signal transduction under adverse environments (Kneeshaw et al. 2014; Mata-Pérez et al. 2019). A recent study has shown that *AtTrx-h2* can improve *Brassica napus*’s salt tolerance by increasing the activities of antioxidant enzymes and biomass. The *AtTrx-h2* maybe a promising genetic resource to boost salt stress tolerance in plants. (Ji et al. 2020). In the special *Bv*M14, both increased levels of TrxH1 and increased redox PTMs seem to be required for enhancing the antioxidant system under salt stress. POD Clot proteins was first identified in *Drosophila*, it is an essential for the biosynthesis of drosopterin (an eye pigment) and the protein were supposed to be GSH-dependent enzymes (Giordano et al. 2003). Clot belongs to classes of atypical Trxs. However, in plants, how Clot plays a role in stress responses is not clear. Plants remove ROS by antioxidative enzymes except for Trxs, which protect plants from oxidative damage (Choudhury et al. 2017). APX, CAT, POD and SOD are key factors in plant under salt stress. Overexpression of their corresponding genes led to higher antioxidant enzyme activities and boost the ROS detoxification pathway related genes’ expression compared to those in control plants under salt stress (Ahmad et al. 2008; Wang et al. 2009; Li et al. 2020). Two cystathionine-β-synthase domain-containing proteins (CBSX1 and CBSX2) were increased under salt stress. It was reported that CBSX1 and CBSX2 as the redox regulators can directly regulate the activation of Trxs in the chloroplasts. Overexpressed CBSX1 and CBSX2 can promoted plant growth and development by increasing Trxs, meanwhile they could modulate their target proteins (Jung et al. 2013; Yoo et al. 2011; Shin et al. 2020).

### Photosynthesis proteins in *BvM14* response to salt stress

Under salt stress, stomatal closure restricts carbon dioxide intake, and thus impaired photosynthesis. Stress tolerant plants can maintain capacity for photosynthesis to meet the energy need (Kosova et al. 2011). In this work, 12 and 4 photosynthesis proteins were identified in redox proteomics and in total proteomics, respectively (Table 1; Additional file 4: Table S2). The 12 photosynthesis proteins include rubredoxin (Rub), rubredoxin-like superfamily protein (Rubl), three ribulose bisphosphate carboxylases/oxygenase activases (Rubisco), photosystem I reaction center subunit III (PSI-RC), ferredoxin (Fd), fructose-bisphosphate aldolase(FBA), fedoheptulose-1,7-bisphosphatase (SBPase), phosphoribulokinase (PRK), Calvin cycle protein CP12 (CP12) and NADH dehydrogenase [ubiquinone] 1 beta subcomplex subunit 7-like (NDUFB7) (Table 1; Fig. 5, Additional file 4: Table S2). The Fds involved in photosynthesis reside within the thylakoids in the chloroplasts or at their cytoplasmic side in cyanobacteria. They are key components of the photosynthetic electron transport chain, acting as main donors of electrons to the regulatory redox protein thioredoxin (Hanke et al. 2013; Buchanan et al. 2005). Fds also mediate electrons to O_2_ (Mehler reaction) and to some of the cyclic electron transport pathways (Shahak et al. 1981; Shikanai et al. 2007; Strand et al. 2017; Marcus et al. 2020). In plants, ROS formation are in the electron transport chains (ETC) of the chloroplasts and the mitochondria. At low levels, ROS are key factors in physiological redox signaling when plants response to stresses, while on the contrary, they are associated with oxidative stress (Gómez et al. 2020). In photosystem I (PSI), the electron transport chain light energy is drived electrons to the acceptor molecule. Sedoheptulose-1,7-bisphosphatase (SBPase) plays key role in the Calvin cycle, which produces the substrate (RuBP) for Rubisco. The electrons of PSI reduce Fd by the enzyme ferredoxin/thioredoxin reductase, which in turn leads to the reduction of thioredoxin f, Finally, trxs activate the SBPase enzyme can promote Cys-52 and Cys-57 to form two thiol groups by reducing the disulfide bond between them (Christine et al. 1999). Redox regulation of the photosynthesis-related proteins has been well-known, but how they change in terms of protein levels and redox states under salt stress has been rarely reported.

### Stress and defense proteins in *BvM14* response to salt stress

Plants experiencing salt stress often exhibit osmotic stress, ionic stress and oxidative stress, which can lead to the accumulation of ROS and malondialdehyde (Jiang et al. 2020; Zhao et al. 2021). Moreever, stress and defense related proteins have been studied under adverse environments (Liu et al. 2018). Under salt stress, we identified stress protein DDR48 in total protein level and DUF642 protein in redox proteomics. In Arabidopsis, four DNA damage-inducible genes (*DDR*) were induced under osmotic stress. These genes have two sets of different osmotic stress-inducible promoters. The *DDR48* was regulated by a different promoter than the one operating in the other three genes under osmotic stress. One significant difference between the two sets of promoters is their sensitivity to different salt conditions (Miralles et al. 1995). As to DUF642, it was a positive regulator of pectin methylesterase (PME) activity (Zúñiga-Sánchez et al. 2014). The *AhDGR2* gene, encoding the DUF642 protein, was significantly up-regulated in roots and leaves of young *A. hypochondriacus* plants under water-deficit and salt stress, suggesting its participation in abiotic stress resistance (Palmeros-Suárez et al. 2017). Here in *BvM14*, we did not observe increase in DUF642 protein levels, but detected for the first time it was oxidized under 400 mM NaCl. It is not known whether oxidation decreases or increases its activity.

### Transport proteins in *BvM14* response to salt stress

In this study, non-specific lipid-transfer protein (nsLTP), thylakoid luminal 17.4 kDa protein (TL17) and mitochondrial import inner membrane translocase subunit (TIM8) were reduced in response to the salt stress. Mitochondrial outer membrane protein porin of 36 kDa (MOM) and trigger factor-like protein (TIG) were increased at the protein level. To date, many LTPs have been described in multiple species, such as Arabidopsis, cotton, wheat, rice, and tobacco (Kinlaw et al. 1994; Kader et al. 1997; Feng et al. 2004; Liu et al. 2006; Boutrot et al. 2008). For example, overexpression a potato *nsLTP1* contributed to the reduced the accumulation of ROS induced by boosting the expression of antioxidant enzyme genes under adverse stresses (Gangadhar et al. 2016). In plants, like nucleus and chloroplasts, mitochondria have two membranes: outer and inner mitochondrial membranes. The existence of a double membrane capsule defines four kinds of mitochondrial sub-compartments with different structures and functions: mitochondrial outer membrane (MOM), mitochondrial inner membrane (MIM), inter membrane space (IMS) and matrix (Schneiter et al. 1999; Dukanovic et al. 2011). In this work, MOM and IMS translocase subunits (TIM8) were differential expressed. It was shown that protein import into mitochondria was changed under adverse stresses that also inhibited mitochondrial functions (Taylor et al. 2003). Arabidopsis mitochondrial proteomics also revealed negative effects of oxidative stress and respiratory inhibitors on important mitochondrial functions (Sweetlove et al. 2003).

### Transcriptional regulation of redox proteins and proteins in *BvM14* response to salt stress

The work showed many protein levels changes and redox level changes under salt stress treatments. Gene transcription can result in the protein level changes. In addition, stress and defense, ROS homeostasis and photosynthesis changes may be affected by redox protein level and protein level changes. The transcriptional levels of the 18 genes encoding for the proteins, the transcriptional level changes of 12 genes stayed in synchronization with the corresponding redox level trend and total protein level trend (Fig. 4, Additional file 4: Table S4), indicating interesting regulatory mechanisms at transcriptional level and PTM level. It should be noted that PTM studies in plant salt response are underrepresented in present knowledge. The identification and cysteine site-mapping of the 42 redox proteins in this work highlight the significance of redox PTMs in the *BvM14* salt stress response (Additional file 9: Table S7).

## Conclusions

The iodoTMTRAQ double labeling quantitative proteomics identified 1290 proteins in the *BvM14*, of which 80 proteins and 42 redox-responsive proteins showed differential changes under salt stress. The salt-stress responsive proteins and redox modified proteins were mainly involved in metabolism, transport, biosynthesis, transcription related, signal transduction, stress and defense, ROS homeostasis and photosynthesis. The results have shown total protein changes and protein redox changes (with more than 53 redox sites in 42 proteins) in different cellular pathways and processes in the *BvM14* plant short-term salt stress response. Obviously, the potential salt response mechanisms involve many different components, pathways and processes (Fig. 5). The interesting findings from this quantitative redox proteomics study include: (1) Several different proteins exhibited significant changes under short term salt stress, including thioredoxin-like 3–1, peroxidase and EG45-like domain containing proteins; (2) redox modifications responsive to the salt stress are not limited to ROS homeostasis and photosynthesis. They were distributed in key physiological processes including transport, transcription, metabolism, and stress and defense (Fig. 5). This explains how the *BvM14* plants can rapidly perceived salt stress, make appropriate changes in cellular biochemical and physiological processes, and adapt for long-term growth and development. As the phosphorylation study has discovered many novel proteins (Yu et al. 2016), this work on redox proteomics has revealed many redox responsive proteins and redox modifications. For example, reduction of extracellular ribonuclease LE-like and nsLTP is a novel discovery. For future research, we will focus on resolving the functional implication and significance of these redox PTM events in plant salt stress response and tolerance.

## Supplementary Information

**Additional file 1: Figure S1**. Singular enrichment analysis (SEA) for redox proteins in biological process (A), cellular components (B) and molecular function (C) was conducted using AgriGO. Each box shows the GO term, GO description, the number mapping the GO and total number of query in the backgroud. Box color indicates levels of statistical significance. More statistically significant nodes result in darker red color.

**Additional file 2: Figure S2**. Singular enrichment analysis (SEA) for total proteins in biological process (A), cellular components (B) and molecular function (C) was conducted using AgriGO. Each box shows the GO term, GO description, the number mapping the GO and total number of query in the backgroud. Box color indicates levels of statistical significance. More statistically significant nodes result in darker red color.

**Additional file 3: Table S1**. List of the identified 1290 proteins from BvM14 leaves between control and NaCl treatment using LC-MS/MS.

**Additional file 4: Table S2**. List of 80 differentially expressed proteins from BvM14 leaves between control and NaCl treatment using LC-MS/MS.

**Additional file 5: Table S3**. List of 42 differential redox proteins from BvM14 leaves between control and NaCl treatment using LC-MS/MS.

**Additional file 6: Table S4**. List of the primer sequences for the 19 genes tested by qRT-PCR in Figure 4.

**Additional file 7: Table S5**. Transcriptional level, redox level and protein expressing pattern of seven differential redox proteins and 11 differential proteins.

**Additional file 8: Table S6**. List of protein IDs shown in Figure 5.

**Additional file 9: Table S7**. MS/MS spectra showing redox modified cysteine sites.

## Data Availability

The data and materials used and analyzed in the current study can be provided by the corresponding author for scientific, non‐profit purposes.

## References

[CR1] Ahmad R, Kim MD, Back KH, Kim HS, Lee HS, Kwon SY (2008). Stress-induced expression of choline oxidase in potato plant chloroplasts confers enhanced tolerance to oxidative, salt, and drought stresses. Plant Cell Rep.

[CR2] Baez NOD, Reisz JA, Furdui CM (2015). Mass spectrometry in studies of protein thiol chemistry and signaling: opportunities and caveats. Free Radic Biol Med.

[CR3] Boutrot F, Chantret N, Gautier MF (2008). Genome wide analysis of the rice and *Arabidopsis* non-specifific lipid transfer protein (*nsLtp*) gene families and identification of wheat *nsLtp* genes by EST data mining. BMC Genomics.

[CR4] Buchanan BB, Balmer Y (2005). Redox regulation: a broadening horizon. Annu Rev Plant Biol.

[CR5] Chang IF, Hsu JL, Hsu PH, Sheng WA, Lai SJ, Lee C, Chen CW, Hsu JC, Wang SY, Wang LY, Chen CC (2012). Comparative phosphoproteomic analysis of microsomal fractions of Arabidopsis thaliana and Oryza sativa subjected to high salinity. Plant Sci.

[CR6] Choudhury FK, Rivero RM, Blumwald E, Mittler R (2017). Reactive oxygen species, abiotic stress and stress combination. Plant J.

[CR7] Raines CA, Lloyd JC, Dyer TA (1999). New insights into the structure and function of sedoheptulose-1,7-bisphosphatase; an important but neglected Calvin cycle enzyme. J Exp Bot.

[CR8] Claiborne A, Yeh JI, Mallett TC, Luba J, Crane EJ, Charrier V, Parsonage D (2005). Protein-sulfenic acids: diverse roles for an unlikely player in enzyme catalysis and redox regulation. Biochemistry.

[CR9] Conesa A, Götz S, García-Gómez JM, Terol J, Talón M, Robles M (2005). Blast2GO: a universal tool for annotation, visualization and analysis in functional genomics research. Bioinformatics.

[CR10] Dave R, Tripathi RD, Dwivedi S, Tripathi P, Dixit G, Sharma YK, Trivedi PK, Corpas FJ, Barroso JB, Chakrabarty D (2012). Arsenate and arsenite exposure modulate antioxidants and amino acids in contrasting arsenic accumulating rice (*Oryza sativa* L.) genotypes. Hazard Mater.

[CR11] Dukanovic J, Rapaport D (2011). Multiple pathways in the integration of proteins into the mitochondrial outer membrane. Biochim Biophys Acta.

[CR12] Farooq MA, Gill RA, Islam F, Ali B, Liu H, Xu J, He S, Zhou W (2016). Methyl jasmonate regulates antioxidant defense and suppresses arsenicuptake in *Brassica napus* L. Front Plant Sci.

[CR13] Feng JX, Ji SJ, Shi YH, Wei G, Zhu YX (2004). Analysis of five differentially expressed gene families in fast elongating cotton fiber. Acta Biochim Biophys Sin.

[CR14] Gangadhar BH, Sajeesh K, Venkatesh J, Baskar V, Abhinandan K, Yu JW, Prasad R, Mishra RK (2016). Enhanced tolerance of transgenic potato plants over-expressing non-specific lipid transfer protein-1 (StnsLTP1) against multiple abiotic stresses. Front Plant Sci.

[CR15] Ghoulam C, Foursy A, Fares K (2002). Effects of salt stress on growth, inorganic ions and proline accumulation in relation to osmotic adjustment in five sugar beet cultivars. Environ Exp Bot.

[CR16] Giordano E, Peluso I, Rendina R, Digilio A, Furia M (2003). The clot gene of *Drosophila melanogaster* encodes a conserved member of the thioredoxin-like protein superfamily. Mol Genet Genomics.

[CR17] Gómez R, Figueroa N, Melzer M, Hajirezaei MR, Carrillo N, Lodeyro AF (2020). Photosynthetic characterization of flavodoxin-expressing tobacco plants reveals a high light acclimation-like phenotype. Biochim Biophys Acta Bioenerg.

[CR18] Gupta V, Carroll KS (2013). Sulfenic acid chemistry, detection and cellular lifetime. Biochim Biophys Acta.

[CR19] Hanke G, Mulo P (2013). Plant type ferredoxins and ferredoxin-dependent metabolism. Plant Cell Environ.

[CR20] Heppner DE, Janssen-Heininger YMW, Van der Vliet A (2017). The role of sulfenic acids in cellular redox signaling: reconciling chemical kinetics and molecular detection strategies. Arch Biochem Biophys.

[CR21] Heppner DE, Hristova M, Ida T, Mijuskovic A, Dustin CM, Bogdándi V, Fukuto JM, Dick TP, Nagy P, Li J, Akaike T, Vliet A (2018). Cysteine perthiosulfenic acid (Cys-SSOH): a novel intermediate in thiol-based redox signaling?. Redox Biol.

[CR22] Howat D (2000) Acceptable salinity, sodicity and pH values for boreal forest reclamation. In: ESD. Alberta Environment, Edmonton Alberta. pp. 2–191.

[CR23] Hsu JL, Wang LY, Wang SY, Lin CH, Ho KC, Shi FK, Chang IF (2009). Functional phosphoproteomic profiling of phosphorylation sites in membrane fractions of salt-stressed Arabidopsis thaliana. Proteome Sci.

[CR24] Ji D, Matthew J, Gaffrey WQ (2017). Quantitative proteomic characterization of redox-dependent post-translational modifications on protein cysteines. Mol Biosystem.

[CR25] Ji MG, Park HJ, Cha JY, Kim JA, Shin GI, Jeong SY, Lee ES, Yun DJ, Lee SY, Kim WY (2020). Expression of *Arabidopsis thaliana* thioredoxin-h2 in *Brassica napus* enhances antioxidant defenses and improves salt tolerance. Plant Physiol Biochem.

[CR26] Jiang J, Ren X, Li L, Hou R, Sun W, Jiao C, Yang N, Dong Y (2020). H_2_S Regulation of Metabolism in cucumber in response to salt-stress through transcriptome and proteome analysis. Front Plant Sci.

[CR27] Jung KW, Kim YY, Yoo KS, Ok SH, Cui MH, Jeong BC, Yoo SD, Jeung JU, Shin JS (2013). Acystathionine-β-synthase domain-containing protein, CBSX2, regulates endothecial secondary cell wall thickening in anther development. Plant Cell Physiol.

[CR28] Jung HI, Kong MS, Lee BR, Kim TH, Chae MJ, Lee EJ, Jung GB, Lee CH, Sung JK, Kim YH (2019). Exogenous glutathione increases arsenic translocation into shoots and alleviates arsenic-induced oxidative stress by sustaining ascorbate-glutathione homeostasis in rice seedlings. Front Plant Sci.

[CR29] Kader JC (1997). Lipid transfer proteins: a puzzling family of plant proteins. Trends Plant Sci.

[CR30] Kemp M, Go YM, Jones DP (2008). Nonequilibrium thermodynamics of thiol/disulfide redox systems: a perspective on redox systems biology. Free Radic Biol Med.

[CR31] Khan PSSV, Hoffmann L, Renaut J, Hausman JF (2007). Current initiatives in proteomics for the analysis of plant salt tolerance. Curr Sci.

[CR32] Khan MN, Siddiqui MH, AlSolami MA, Alamri S, Hu Y, Ali HM, Al-Amri AA, Alsubaie QD, Al-Munqedhi BMA, Al-Ghamdi A (2020). Crosstalk of hydrogen sulfide and nitric oxide requires calcium to mitigate impaired photosynthesis under cadmium stress by activating defense mechanisms in Vigna radiata. Plant Physiol Biochem.

[CR33] Kinlaw CS, Gerttula SM, Carter MC (1994). Lipid transfer protein genes of loblolly pine are members of a complex gene family. Plant Mol Biol.

[CR34] Kitajima S (2008). Hydrogen peroxide-mediated inactivation of two chloroplastic peroxidases, ascorbate peroxidase and 2-Cys peroxiredoxin. Photochem Photobiol.

[CR35] Kitajima S, Kurioka M, Yoshimoto T, Shindo M, Kanaori K, Tajima K, Oda K (2008). A cysteine residue near the propionate side chain of heme is the radical site in ascorbate peroxidase. FEBS J.

[CR36] Kneeshaw S, Gelineau S, TadaY LGJ, Spoel SH (2014). Selective protein denitrosylation activity of thioredoxin-h5 modulates plant immunity. Mol Cell.

[CR37] Kosova K, Vitamvas P, Prasil IT, Renaut J (2011). Plant proteome changes under abiotic stress-contribution of proteomics studies to understanding plant stress response. J Proteomics.

[CR38] Li H, Cao H, Wang Y, Pang Q, Ma C, Chen S (2009). Proteomic analysis of sugar beet apomictic monosomic addition line M14. J Proteomics.

[CR39] Li H, Pan Y, Zhang Y, Wu C, Ma C, Yu B, Zhu N, Koh J, Chen S (2015). Salt stress response of membrane proteome of Sugar beet monosomic addition line M14. J Proteomics.

[CR40] Li C, Ji J, Wang G, Li Z, Wang Y, Fan Y (2020). Over-expression of *LcPDS*, *LcZDS*, and *LcCRTISO*, genes from wolfberry for carotenoid biosynthesis, enhanced carotenoid accumulation, and salt tolerance in Tobacco. Front Plant Sci.

[CR41] Lin Y, Chen G, Lin H, Lin M, Wang H, Lin Y (2020). Chitosan postharvest treatment suppresses the pulp breakdown development of longan fruit through regulating ROS metabolism. Int J Biol Macromol.

[CR42] Liu K, Jiang H, Moore S, Watkins C, Jahn M (2006). Isolation and characterization of a lipid transfer protein expressed in ripening fruit of *Capsicum chinense*. Planta.

[CR43] Liu YL, Cao D, Ma LL, Jin XF, Yang PF, Ye F (2018). TMT-based quantitative proteomics analysis reveals the response of tea plant (*Camellia sinensis*) to fluoride. J Proteomics.

[CR44] Marcus Y, Gurevitz M (2020). Ferredoxin-mediated reduction of 2-nitrothiophene inhibits photosynthesis: mechanism and herbicidal potential. Biochem J.

[CR45] Mata-Pérez C, Spoel SH (2019). Thioredoxin-mediated redox signaling in plant immunity. Plant Sci.

[CR46] Meyer Y, Belin C, Delorme-Hinoux V, Reichheld JP, Riondet C (2012). Thioredoxin and glutaredoxin systems in plants: molecular mechanisms, crosstalks, and functional significance. Antioxid Redox Signal.

[CR47] Miller G, Suzuki N, Ciftci-Yilmaz S, Mittler R (2010). Reactive oxygen species homeostasis and signalling during drought and salinity stresses. Plant Cell Environ.

[CR48] Miralles VJ, Serrano R (1995). A genomic locus in *Saccharomyces cerevisiae* with four genes up-regulated by osmotic stress. Mol Microbiol.

[CR49] Mock HP, Dietz KJ (2016). Redox proteomics for the assessment of redox-related posttranslational regulation in plants. Biochim Biophys Acta.

[CR50] Navrot N, Finnie C, Svensson B, Hägglund P (2011). Plant redox proteomics. J Proteomics.

[CR51] Palmeros-Suárez PA, Massange-Sánchez JA, Sánchez-Segura L, Martínez-Gallardo NA, Espitia-Rangel E, Gómez-Leyva JF, Délano-Frier JP (2017). *AhDGR2*, an amaranth abiotic stress-induced DUF642 protein gene, modifies cell wall structure and composition and causes salt and ABA hyper-sensibility in transgenic Arabidopsis. Planta.

[CR52] Parker J, Zhu N, Zhu M, Chen S (2012). Profiling thiol redox proteome using isotope tagging mass spectrometry. J vis Exp.

[CR53] Parker J, Balmant K, Zhu F, Zhu N, Chen S (2015). cysTMTRAQ-An integrative method for unbiased thiol based redox proteomics. Mol Cell Proteomics.

[CR54] Pichon M, Gaymard A, Josset L, Valette M, Millat G, Lina B, Escuret V (2017). Characterization of oseltamivir-resistant influenza virus populations in immunosuppressed patients using digital-droplet PCR: comparison with qPCR and next generation sequencing analysis. Antiviral Res.

[CR55] Poole LB, Karplus PA, Claiborne A (2004). Protein sulfenic acids in redox signaling. Annu Rev Pharmacol Toxicol.

[CR57] Schneiter R, Brugger B, Sandhoff R, Zellnig G, Leber A, Lampl M, Athenstaedt K, Hrastnik C, Eder S, Daum G, Paltauf F, Wieland FT, Kohlwein SD (1999). Electrospray ionization tandem mass spectrometry (ESI-MS/MS) analysis of the lipid molecular species composition of yeast subcellular membranes reveals acyl chain-based sorting/remodeling of distinct molecular species en route to the plasma membrane. J Cell Biol.

[CR58] Shahak Y, Crowther D, Hind G (1981). The involvement of ferredoxin-NADP^+^ reductase in cyclic electron transport in chloroplasts. Biochim Biophys Acta.

[CR59] Shikanai T (2007). Cyclic electron transport around photosystem I: genetic approaches. Annu Rev Plant Biol.

[CR60] Shin JS, So WM, Kim SY, Noh M, Hyoung S, Yoo KS, Shin JS (2020). CBSX3-Trxo-2 regulates ROS generation of mitochondrial complex II (succinate dehydrogenase) in Arabidopsis. Plant Sci.

[CR61] Strand DD, Fisher N, Kramer DM (2017). The higher plant plastid NAD(P)H dehydrogenase-like complex (NDH) is a high effificiency proton pump that increases ATP production by cyclic electron flow. J Biol Chem.

[CR62] Sweetlove LJ, Heazlewood JL, Herald V, Holtzapffel R, Day DA, Leaver CJ, Millar AH (2002). The impact of oxidative stress on Arabidopsis mitochondria. Plant J.

[CR63] Taylor NL, Rudhe C, Hulet JM, Lithgow T, Glaser E, Day DA, Millar AH, Whelan J (2003). Environmental stresses inhibit and stimulate different protein import pathways in plant mitochondria. FEBS Lett.

[CR64] Thamsen M, Jakob U (2011). The redoxome proteomic analysis of cellular redox networks. Curr Opin Chem Biol.

[CR65] Untergasser A, Nijveen H, Rao X, Bisseling T, Geurts R, Leunissen JA (2007). Primer3 Plus, an enhanced web interface to primer3. Nucleic Acids Res.

[CR66] Wakeel A, Asif AR, Pitann B, Schubert S (2011). Proteome analysis of sugar beet (*Beta vulgaris* L.) elucidatas constitutive adaptation during the first phase of salt stress. J Plant Physiol.

[CR67] Wang WB, Kim YH, Lee HS, Kim KY, Deng XP, Kwak SS (2009). Analysis of antioxidant enzyme activity during germination of alfalfa under salt and drought stresses. Plant Physiol Biochem.

[CR68] Xu X, Wan W, Jiang G, Xi Y, Huang H, Cai J, Chang Y, Duan CG, Mangrauthia SK, Peng X, Zhu JK, Zhu G (2019). Nucleocytoplasmic trafficking of the Arabidopsis WD40 repeat protein XIW1 regulates ABI5 stability and abscisic acid responses. Mol Plant.

[CR69] Yang Y, Guo Y (2018). Elucidating the molecular mechanisms mediating plant salt-stress responses. New Phytol.

[CR70] Yang L, Ma C, Wang L, Chen S, Li H (2012). Salt stress induced proteome and transcriptome changes in sugar beet monosomic addition line M14. J Plant Physiol.

[CR71] Yang L, Zhang Y, Zhu N, Koh J, Ma C, Pan Y, Yu B, Chen S, Li H (2013). Proteomic analysis of salt tolerance in sugar beet monosomic addition line M14. J Proteome Res.

[CR72] Yoo KS, Ok SH, Jeong BC, Jung KW, Cui MH, Hyoung S, Le MR, Song HK, Shin JS (2011). Single cystathionine beta-synthase domain-containing proteins modulate development by regulating the thioredoxin system in Arabidopsis. Plant Cell.

[CR73] Yu B, Li J, Koh J, Dufresne C, Yang N, Qi S, Zhang Y, Ma C, Duong BV, Chen S, Li H (2016). Quantitative proteomics and phosphoproteomics of sugar beet monosomic addition line M14 in response to salt stress. J Proteomics.

[CR74] Yu J, Li Y, Qin Z, Guo S, Li Y, Miao Y, Song C, Chen S, Dai S (2020). Plant chloroplast stress response: insights from thiol redox proteomics. Antioxid Redox Signal.

[CR75] Yuan L, Wang J, Xie S, Zhao M, Nie L, Zheng Y, Zhu S, Hou J, Chen G, Wang C (2019). Comparative proteomics indicates that redox homeostasis is involved in high- and low-temperature stress tolerance in a novel wucai (*Brassica campestris* L.) Genotype. Int J Mol Sci.

[CR76] Zhao S, Zhang Q, Liu M, Zhou H, Ma C, Wang P (2021). Regulation of plant responses to salt stress. Int J Mol Sci.

[CR77] Zúñiga-Sánchez E, Soriano D, Martínez-Barajas E, Orozco-Segovia A, Gamboa-deBuen A (2014). *BIIDXI*, the *At4g32460 DUF642* gene, is involved in pectin methylesterase regulation during *Arabidopsis thaliana* seed germination and plant development. BMC Plant Biol.

