# Supplementary figures and images for: Cys-SH based quantitative redox proteomics of salt induced response in sugar beet monosomic addition line M14
Source: Bot Stud. 2021 Oct 18;62:16. doi: 10.1186/s40529-021-00320-x (PMC8523603; doi:10.1186/s40529-021-00320-x)

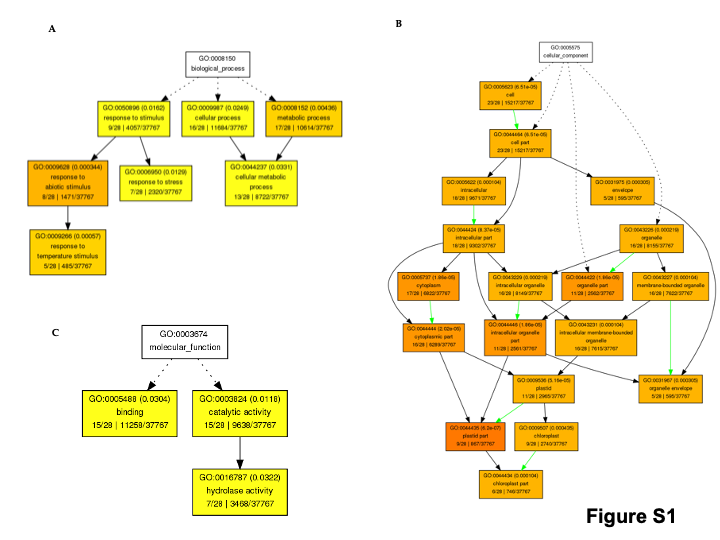

Supplement: Supplementary file 1 — Additional file 1: Figure S1. Singular enrichment analysis (SEA) for redox proteins in biological process (A), cellular components (B) and molecular function (C) was conducted using AgriGO. Each box shows the GO term, GO description, the number mapping the GO and total number of query in the backgroud. Box color indicates levels of statistical significance. More statistically significant nodes result in darker red color. [file 40529_2021_320_MOESM1_ESM.tiff]

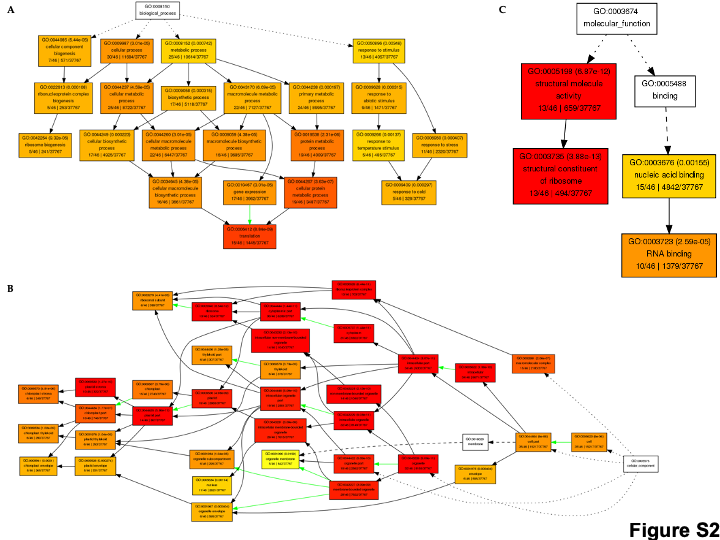

Supplement: Supplementary file 2 — Additional file 2: Figure S2. Singular enrichment analysis (SEA) for total proteins in biological process (A), cellular components (B) and molecular function (C) was conducted using AgriGO. Each box shows the GO term, GO description, the number mapping the GO and total number of query in the backgroud. Box color indicates levels of statistical significance. More statistically significant nodes result in darker red color. [file 40529_2021_320_MOESM2_ESM.tiff]
