# Supplementary material for: Cys-SH based quantitative redox proteomics of salt induced response in sugar beet monosomic addition line M14
Source: Bot Stud. 2021 Oct 18;62:16. doi: 10.1186/s40529-021-00320-x (PMC8523603; doi:10.1186/s40529-021-00320-x)
Supplement: Supplementary file 4 — Additional file 4: Table S2. List of 80 differentially expressed proteins from BvM14 leaves between control and NaCl treatment using LC-MS/MS. [file 40529_2021_320_MOESM4_ESM.doc]

**Supplemental Table S2. List of 80 differentially expressed proteins from sugar beet M14 leaves between control**

**and NaCl treatment using LC-MS/MS (all entries with p < 0.05).**

| No. | Protein ID^a^ | Description | Plant species | salt200/control Ratio^b^ | salt400/control Ratio^c^ | *p*-value | Function^d^ |
| --- | --- | --- | --- | --- | --- | --- | --- |
| 1 | 731355347 | RuBisCO large subunit-binding protein subunit alpha | *Beta vulgaris subsp. vulgaris* | 1.28 | 1.30 | 0.02 | Photosynthesis  (4) |
| 2 | A0A0J8E3S1 | Ferredoxin | *Beta vulgaris subsp. vulgaris* | –– | 1.22 | 0.03 |
| 3 | A0A1U7WY41 | Protease Do-like 8 | *Nicotiana sylvestris* | –– | 1.33 | 0.02 |
| 4 | A0A1D6GF76 | Cytochrome b-c1 complex subunit 7 | *Zea mays* | –– | 1.23 | 0.03 |
| 5 | 1108926884 | Elongation factor Tu | *Beta vulgaris subsp. vulgaris* | –– | 1.22 | 0.04 | Protein synthesis (22) |
| 6 | 731345000 | UPF0426 protein At1g28150 | *Beta vulgaris subsp. vulgaris* | –– | 1.27 | 0.02 |
| 7 | 731367357 | 31 kDa ribonucleoprotein | *Beta vulgaris subsp. vulgaris* | –– | 1.28 | 0.00 |
| 8 | 731333097 | 33 kDa ribonucleoprotein | *Beta vulgaris subsp. vulgaris* | –– | 1.30 | 0.00 |
| 9 | 731324331 | Eukaryotic translation initiation factor 3 subunit E | *Beta vulgaris subsp. vulgaris* | –– | 1.24 | 0.00 |
| 10 | 731317922 | Elongation factor Tu | *Beta vulgaris subsp. vulgaris* | –– | 1.31 | 0.03 |
| 11 | A0A0B2RHJ1 | 40S ribosomal protein S24 | *Glycine soja* | –– | 1.23 | 0.04 |
| 12 | 1108781787 | 50S ribosomal protein L3 | *Beta vulgaris subsp. vulgaris* | –– | 1.25 | 0.01 |
| 13 | A0A0K9PKE9 | 40S ribosomal protein S17 | *Zostera marina* | –– | 1.26 | 0.04 |
| 14 | 731310838 | 50S ribosomal protein L21 | *Beta vulgaris subsp. vulgaris* | –– | 1.28 | 0.00 |
| 15 | 731312233 | 50S ribosomal protein L31 | *Beta vulgaris subsp. vulgaris* | –– | 1.28 | 0.04 |
| 16 | A0A103XHD2 | Ribosomal protein L9 | *Cynara cardunculus var. scolymus* | –– | 1.32 | 0.03 |
| 17 | P22798 | 50S ribosomal protein L15 | *Spinacia oleracea* | –– | 1.32 | 0.00 |
| 18 | 731349570 | 60S acidic ribosomal protein P2A | *Beta vulgaris subsp. vulgaris* | –– | 1.32 | 0.01 |
| 19 | 731350328 | 30S ribosomal protein S5 | *Beta vulgaris subsp. vulgaris* | –– | 1.35 | 0.03 |
| 20 | 731316270 | 50S ribosomal protein L1 | *Beta vulgaris subsp. vulgaris* | –– | 1.38 | 0.01 |
| 21 | 731348660 | 50S ribosomal protein L4 | *Beta vulgaris subsp. vulgaris* | –– | 1.41 | 0.01 |
| 22 | 731345787 | 60S ribosomal protein L12-1 | *Beta vulgaris subsp. vulgaris* | –– | 1.41 | 0.00 |
| 23 | 731324870 | 50S ribosomal protein 5alpha | *Beta vulgaris subsp. vulgaris* | –– | 1.50 | 0.00 |
| 24 | 731359542 | 30S ribosomal protein S17 | *Beta vulgaris subsp. vulgaris* | –– | 1.50 | 0.02 |
| 25 | 731337895 | Nucleosome assembly protein 1;2 | *Beta vulgaris subsp. vulgaris* | –– | 1.65 | 0.04 |
| 26 | 731323749 | 30S ribosomal protein S1 | *Beta vulgaris subsp. vulgaris* | –– | 1.68 | 0.00 |
| 27 | 731362409 | Petal death protein | *Beta vulgaris subsp. vulgaris* | –– | 1.21 | 0.02 | Metabolism (5) |
| 28 | 731322038 | Dihydrolipoyl dehydrogenase 1 | *Beta vulgaris subsp. vulgaris* | –– | 1.38 | 0.04 |  |
| 29 | 731325938 | Rhodanese-like/PpiC domain-containing protein 12 | *Beta vulgaris subsp. vulgaris* | –– | 1.38 | 0.00 |
| 30 | A0A2K3NVX1 | Chloroplast stem-loop binding protein of 41 kDa a chloroplastic-like | *Trifolium pratense* | –– | 1.86 | 0.02 |
| 31 | 731337601 | Protein GOS9 | *Beta vulgaris subsp. vulgaris* | –– | 1.27 | 0.02 |
| 32 | 731316096 | Thioredoxin-like protein Clot | *Beta vulgaris subsp. vulgaris* | –– | 1.25 | 0.04 | Ros homeostasis (6) |
| 33 | 731312103 | Thioredoxin H-type 1 | *Beta vulgaris subsp. vulgaris* | –– | 1.30 | 0.04 |
| 34 | M4DVR1 | Cysteine synthase | *Brassica rapa subsp. pekinensis* | –– | 1.30 | 0.02 |
| 35 | 731327123 | Protein disulfide isomerase-like 1-1 | *Beta vulgaris subsp. vulgaris* | –– | 1.36 | 0.03 |
| 36 | 731312686 | Thylakoid lumenal 29 kDa protein | *Beta vulgaris subsp. vulgaris* | 1.32 | –– | 0.02 |
| 37 | 731346319 | CBS domain-containing protein CBSX3 | *Beta vulgaris subsp. vulgaris* | 1.35 | –– | 0.00 |
| 38 | 731365169 | 20 kDa chaperonin, chloroplastic | *Beta vulgaris subsp. vulgaris* | –– | 1.24 | 0.04 | Protein stability and turnover (4) |
| 39 | A0A2P6PWQ8 | HSP20-like chaperone | *Rosa chinensis* | –– | 1.30 | 0.00 |
| 40 | 731363918 | Proteasome subunit alpha type-5 | *Beta vulgaris subsp. vulgaris* | –– | 1.29 | 0.02 |  |
| 41 | 731337576 | Peptidyl-prolyl cis-trans isomerase Pin1 | *Beta vulgaris subsp. vulgaris* | –– | 1.77 | 0.00 |  |
| 42 | 1108945926 | Rhodanese-like domain-containing protein 15 | *Beta vulgaris subsp. vulgaris* | –– | 1.26 | 0.02 | Stress and defense (2) |
| 43 | 731313572 | Stress protein DDR48 | *Beta vulgaris subsp. vulgaris* | –– | 0.50 | 0.04 |
| 44 | B9RHH4 | Ribonucleoprotein | *Ricinus communis* | –– | 1.80 | 0.03 | Transcription (5) |
| 45 | A0A2I0VI92 | Polyadenylate-binding protein | *Dendrobium catenatum* | –– | 1.26 | 0.03 |
| 46 | 731368824 | Zinc finger CCCH domain-containing protein 11 | *Beta vulgaris subsp. vulgaris* | –– | 1.28 | 0.01 |
| 47 | 731345735 | RNA-binding protein CP29B | *Beta vulgaris subsp. vulgaris* | –– | 1.40 | 0.02 |  |
| 48 | A0A2K3L2Z0 | RNA-binding (RRM/RBD/RNP motif) family protein | *Trifolium pratense* | –– | 1.32 |  |  |
| 49 | 731344143 | Mitochondrial outer membrane protein porin of 36 kDa | *Beta vulgaris subsp. vulgaris* | 1.32 | –– | 0.00 | Transport (5) |
| 50 | 731329823 | Trigger factor-like protein TIG | *Beta vulgaris subsp. vulgaris* | –– | 1.25 | 0.04 |
| 51 | 731365676 | GRIP and coiled-coil domain-containing protein C27D7.02c | *Beta vulgaris subsp. vulgaris* | –– | 1.34 | 0.00 |
| 52 | 731312251 | Nascent polypeptide-associated complex subunit beta-like | *Beta vulgaris subsp. vulgaris* | –– | 1.33 | 0.04 |
| 53 | W6JNH5 | Non-specific lipid-transfer protein | *Suaeda japonica* | –– | 0.46 | 0.03 |
| 54 | 731359069 | Uncharacterized protein LOC104904517 | *Beta vulgaris subsp. vulgaris* | –– | 1.20 | 0.02 | Unknown (27) |
| 55 | A0A0K9Q959 | Uncharacterized protein | *Spinacia oleracea* | –– | 1.21 | 0.04 |
| 56 | 731315114 | Uncharacterized protein LOC104903583 | *Beta vulgaris subsp. vulgaris* | –– | 1.22 | 0.02 |
| 57 | 731372115 | Uncharacterized protein LOC104883422 isoform X1 | *Beta vulgaris subsp. vulgaris* | –– | 1.23 | 0.03 |
| 58 | V4TS47 | Uncharacterized protein | *Citrus clementina* | –– | 1.23 | 0.03 |
| 59 | M5WXI2 | Uncharacterized protein | *Prunus persica* | –– | 1.24 | 0.00 |
| 60 | 731339723 | Uncharacterized protein LOC104896030 | *Beta vulgaris subsp. vulgaris* | –– | 1.24 | 0.04 |
| 61 | A0A0J8BEJ7 | Uncharacterized protein | *Beta vulgaris subsp. vulgaris* | –– | 1.25 | 0.00 |
| 62 | A0A0J8B4M5 | Uncharacterized protein | *Beta vulgaris subsp. vulgaris* | –– | 1.25 | 0.00 |
| 63 | A0A0K9RJE8 | Uncharacterized protein | *Spinacia oleracea* | –– | 1.26 | 0.01 |
| 64 | 731329733 | Uncharacterized protein LOC104891716 | *Beta vulgaris subsp. vulgaris* | –– | 1.26 | 0.04 |
| 65 | A0A2H9ZYY8 | Uncharacterized protein | *Apostasia shenzhenica* | –– | 1.27 | 0.00 |
| 66 | A0A0J8BPT8 | Uncharacterized protein | *Beta vulgaris subsp. vulgaris* | –– | 1.27 | 0.03 |
| 67 | S8DN84 | Uncharacterized protein (Fragment) | *Genlisea aurea* | –– | 1.27 | 0.00 |
| 68 | 731335861 | Uncharacterized protein At5g48480 | *Beta vulgaris subsp. vulgaris* | –– | 1.29 | 0.03 |
| 69 | A0A0J8B8J2 | Uncharacterized protein | *Beta vulgaris subsp. vulgaris* | –– | 1.30 | 0.04 |
| 70 | V7BUF6 | Uncharacterized protein | *Phaseolus vulgaris* | –– | 1.32 | 0.02 |
| 71 | 731371557 | Uncharacterized protein At4g28440 | *Beta vulgaris subsp. vulgaris* | –– | 1.34 | 0.01 |
| 72 | M0RXB6 | Uncharacterized protein | *Musa acuminata subsp. malaccensis* | –– | 1.35 | 0.00 |
| 73 | A0A0J8FKL0 | Uncharacterized protein | *Beta vulgaris subsp. vulgaris* | –– | 1.35 | 0.02 |
| 74 | A0A0K9QVH3 | Uncharacterized protein | *Spinacia oleracea* | –– | 1.37 | 0.03 |
| 75 | J3NAI0 | Uncharacterized protein | *Oryza brachyantha* | –– | 1.38 | 0.03 |
| 76 | A0A087H4X4 | Uncharacterized protein | *Arabis alpina* | –– | 1.40 | 0.03 |
| 77 | 731326027 | Uncharacterized protein LOC104890134 | *Beta vulgaris subsp. vulgaris* | –– | 1.41 | 0.02 |
| 78 | A0A0J8CM43 | Uncharacterized protein | *Beta vulgaris subsp. vulgaris* | –– | 1.42 | 0.01 |
| 79 | A0A0J8B4P1 | Uncharacterized protein | *Beta vulgaris subsp. vulgaris* | –– | 2.75 | 0.04 |
| 80 | 731359069 | Uncharacterized protein LOC104904517 | *Beta vulgaris subsp. vulgaris* | –– | 1.20 | 0.02 |

^a^Protein ID, gi number of NCBI; ^b^salt200/control Ratio, a relative abundance of protein at protein level (200 mM NaCl treatment versus control), *P*-value < 0.05; ^c^salt400/control Ratio, a relative abundance of protein at protein level (400 mM NaCl treatment versus control) , P-value < 0.05;  ^d^Function, according to Blast2GO software. The number in brackets, indicate the numbers of proteins in corresponding function; Induction, only detected in salt treated samples, but not in control samples; Suppression, only detected in control samples, but not in salt treated samples; ––, no change of protein level.
