# Supplementary material for: Cys-SH based quantitative redox proteomics of salt induced response in sugar beet monosomic addition line M14
Source: Bot Stud. 2021 Oct 18;62:16. doi: 10.1186/s40529-021-00320-x (PMC8523603; doi:10.1186/s40529-021-00320-x)
Supplement: Supplementary file 6 — Additional file 6: Table S4. List of the primer sequences for the 19 genes tested by qRT-PCR in Figure 4. [file 40529_2021_320_MOESM6_ESM.docx]

Supplemental Table S4. List of the primer sequences for 18 genes in Figure 4

| No. | Protein ID^a^ | Gene name^b^ | Primer sequence^c^ |
| --- | --- | --- | --- |
| 1 | Q8MC96 | *BvM14-atpC* | *atpC*s：5-TCGCCAAATTACCTGAGGCC-3  *atpC*as：5-AGCAGCTTGCCAAACAAAGG-3 |
| 2 | A0A314V1F4 | *BvM14-RNase LE* | *RNase LE* s：5-GGCTGCAATGTTGATCCAGC-3  CBPas：5-GGGAAGGACAATTGCTCCGA-3 |
| 3 | A0A161DY72 | *BvM14-DUF642* | *DUF642*s：5-CAGGCAAAGACACGGCAAAA-3  *DUF642*as：5-GGGTTCGAGTTTGGGTAGCA-3 |
| 4 | D7KW69 | *BvM14-Fd-1* | *Fd-1*s：5-CAGCCACCTTTGTGCCAAAA-3  *Fd-1*as：5-AACTCTTGAGTGCCCGATGG-3 |
| 5 | Q9M0C2 | *BvM14-EGC1* | *EGC1*s：5-GGTAGGTCGTACCGTGTCAC-3  *EGC1*as：5-CGGGGCAAAGGTCTACAACT-3 |
| 6 | A0A0K9QDU1 | *BvM14-POD* | *POD*s：5-ACTTGCCAAGGAGTAGAGGG-3  *POD*as：5-AGAAACAACTCCTGGGCACA-3 |
| 7 | A0A1J6IUE1 | *BvM14-Trx3-1* | *Trx3-1*s：5-AGCGGCCGAATATGATACCA-3  *Trx3-1*as：5-CCGCGCTTGACAAGAGTTTG-3 |
| 8 | 731355347 | *BvM14-Rubisco LSU* | *Rubisco LSU*s：5-attcctcaacgagcgcagaa-3  *Rubisco LSU*as：5-tctccctctgggaccaagag-3 |
| 9 | A0A0J8E3S1 | *BvM14-Fd* | *FdX*s：5-ccgacctgcacgaatacctt-3  *FdX*as：5-gtaaggatgctctcgtcggg-3 |
| 10 | 731322038 | *BvM14-DLD1* | *DLD1*s：5-cagtgagttctcgccgagtt-3  *DLD1*as：5-aacgccagctccaatgatga-3 |
| 11 | 731316096 | *BvM14-clot* | *clot*s：5-tccctcagttggtgtcctga-3  *clot*as：5-taggggtcctccaagtaggc-3 |
| 12 | 731312103 | *BvM14-TrxH1* | *TrxH1*s：5-ccgcttcatcgcaccaattc-3  *TrxH1*as：5-aaaacaaaggtcggcatggc-3 |
| 13 | M4DVR1 | *BvM14-Cys* | *Cys*s：5-ttgctggtgttggaactggt-3  *Cys*as：5-tccagaggatatgcccacca-3 |
| 14 | 731327123 | *BvM14-PDIL1-1* | *PDIL1-1*s：5-aatgctgctgctttgaaggc-3  *PDIL1-1*as：5-tcgagcttgaagtgctggag-3 |
| 15 | 731312686 | *BvM14-TL29* | *TL29*s：5-tcgcgtatgctgacctcatc-3  *TL29*as：5-ccccactgtccagaagaacc-3 |
| 16 | 731346319 | *BvM14-CBSX3* | *CBSX3*s：5-tttgcacgctttgagtctgc-3  *CBSX3*as：5-tacctcttgctctccaggct-3 |
| 17 | 731313572 | *BvM14-DDR48* | *DDR48*s：5-atcgctacggttccgatgac-3  *DDR48*as：5-gagccatagcggtcagtctc-3 |
| 18 | W6JNH5 | *BvM14-nsLTP* | *nsLTP*s：5-atgtgcatgttggtggttgc-3  *nsLTP*as：5-ccgcaagcagttttcctgtc-3 |
| 19 |  | *BvM14-18S rRNA^d^* | *18S*-F：5-CCC CAA TGG ATC CTC GTT A-3  *18S*-R：5-TGA CGG AGA ATT AGG GTT CG-3 |

^a^ Protein ID , gi number of NCBI; ^b^Gene name,1-7, the genes of 7 differential redox proteins ,8-18, the genes of 11 differential proteins ；^c^Primer sequence, the primer sequences for 18 genes (s represents sense primer of the gene, as represents anti-sense primer of the gene); *^d^18S rRNA*, the reference gene for real-time PCR in sugar beet M14.
