# Supplementary material for: Cys-SH based quantitative redox proteomics of salt induced response in sugar beet monosomic addition line M14
Source: Bot Stud. 2021 Oct 18;62:16. doi: 10.1186/s40529-021-00320-x (PMC8523603; doi:10.1186/s40529-021-00320-x)
Supplement: Supplementary file 7 — Additional file 7: Table S5. Transcriptional level, redox level and protein expressing pattern of seven differential redox proteins and 11 differential proteins. [file 40529_2021_320_MOESM7_ESM.docx]

Supplemental Table S5. The transcriptional level, redox protein level and protein expressing pattern of 7 differential redox proteins and 11 differential proteins

| No. | Protein ID^a^ | Gene name^b^ | qRT-PCR  (200mM  /control^c^) | qRT-PCR  (400mM  /control^d^) | Protein name^e^ | Redox level (200mM  /control^f^) | Redox level (400mM /control^g^) | Protein  Abundanc  (200mM  /control^h^) | Protein  Abundanc  (400mM  /control^i^) |
| --- | --- | --- | --- | --- | --- | --- | --- | --- | --- |
| 1 | Q8MC96 | *BvM14-atpC* |  | –– | BvM14-atpC |  | –– | –– | –– |
| 2 | A0A314V1F4 | *BvM14-RNase LE* | –– |  | BvM14-RNase LE | –– |  | –– | –– |
| 3 | A0A161DY72 | *BvM14-DUF642* | –– |  | BvM14-DUF642 | –– |  | –– | –– |
| 4 | D7KW69 | *BvM14-Fd-1* | –– |  | BvM14-Fd-1 | –– |  | –– | –– |
| 5 | Q9M0C2 | *BvM14-EGC1* |  | –– | BvM14-EGC1 |  | –– | –– | –– |
| 6 | A0A0K9QDU1 | *BvM14-POD* | –– |  | BvM14-POD | –– |  | –– | –– |
| 7 | A0A1J6IUE1 | *BvM14-Trx3-1* | –– |  | BvM14-Trx3-1 | –– |  | –– | –– |
| 8 | 731355347 | *BvM14-Rubisco LSU* |  |  | BvM14-Rubisco LSU | ■ | ■ |  |  |
| 9 | A0A0J8E3S1 | *BvM14-Fd* | –– |  | BvM14-Fd | ■ | ■ | –– |  |
| 10 | 731322038 | *BvM14-DLD1* | –– |  | BvM14-DLD1 | ■ | ■ | –– |  |
| 11 | 731316096 | *BvM14-clot* | –– |  | BvM14-clot | ■ | ■ | –– |  |
| 12 | 731312103 | *BvM14-TrxH1* | –– |  | BvM14-TrxH1 | ■ | ■ | –– |  |
| 13 | M4DVR1 | *BvM14-Cys* | –– |  | BvM14-Cys | ■ | ■ | –– |  |
| 14 | 731327123 | *BvM14-PDIL1-1* | –– |  | BvM14-PDIL1-1 | ■ | ■ | –– |  |
| 15 | 731312686 | *BvM14-TL29* |  | –– | BvM14-TL29 | ■ | ■ |  | –– |
| 16 | 731346319 | *BvM14-CBSX3* |  | –– | BvM14-CBSX3 | ■ | ■ |  | –– |
| 17 | 731313572 | *BvM14-DDR48* | –– |  | BvM14-DDR48 | ■ | ■ | –– |  |
| 18 | W6JNH5 | *BvM14-nsLTP* | –– |  | BvM14-nsLTP | ■ | ■ | –– |  |

^a^ Protein ID , gi number of NCBI; ^b^Gene name, the genes of 7 differential redox proteins and 11 differential protein ；^c^qRT-PCR (200mM/control), the transcriptional level of the gene between 200mM salt treatment and control; ^d^RT-PCR (400mM/control), the transcriptional level of the gene between 400mM salt treatment and control; ^e^Protein name, the genes of 7 differential redox proteins and 11 differential protein ; ^f^redox level (200mM/control), the ratio of redox peptide between 200mM salt treatment and control; ^g^redox level (400mM/control), the ratio of redox peptide between 400mM salt treatment and control; ^h^Protein abundance (200mM/control), the ration of protein abundance between 200mM salt treatment and control; ^i^Protein abundance (400mM/control), the ration of protein abundance between 400mM salt treatment and control; –– , no change； , downregulated expression； , upregulated expression; ■, no redox modification. (The transcriptional level: up-regulation: 2^−ΔΔCT^>1, down-regulation: 2^−ΔΔCT^<1; The redox level and protein level: up-regulation: Fold change>1.2 and p-value <0.05, down-regulation: Fold change <0.8 and p-value <0.05).
