# Supplementary material for: Cys-SH based quantitative redox proteomics of salt induced response in sugar beet monosomic addition line M14
Source: Bot Stud. 2021 Oct 18;62:16. doi: 10.1186/s40529-021-00320-x (PMC8523603; doi:10.1186/s40529-021-00320-x)
Supplement: Supplementary file 8 — Additional file 8: Table S6. List of protein IDs shown in Figure 5. [file 40529_2021_320_MOESM8_ESM.docx]

Supplemental Table S6. List of the protein IDs in Figure 5

| No. | Protein ID^a^ | Protein name (Abbreviation) | Protein name (Full name) |
| --- | --- | --- | --- |
| 1 | A0A1J6IUE1 | Trx3-1 | Thioredoxin-like 3-1 |
| 2 | 731316096 | Clot | Thioredoxin-like protein Clot |
| 3 | 731312103 | TrxH1 | Thioredoxin H-type 1 |
| 4 | A0A0K9QDU1 | POD | Peroxidase |
| 5 | A0A1S3CE63 | RD19A | Cysteine proteinase RD19a-like |
| 6 | A0A314V1F4 | RNase LE | Extracellular ribonuclease LE-like |
| 7 | O50036 | HSP70 | Heat shock 70 protein |
| 8 | A0A0K9RNM7/W6JNH5 | nsLTP | Non-specific lipid-transfer protein |
| 9 | 731313572 | DDR48 | Stress protein DDR48 |
| 10 | A0A161DY72 | DUF642 | DUF642 |
| 11 | A0A1R3GZ43 | EGF | EGF-like calcium-binding protein |
| 12 | A0A1D1Y6P4 | VSR2 | Vacuolar-sorting receptor 2 |
| 13 | A0A1U7ZGK1 | TIM8 | Mitochondrial import inner membrane translocase subunit TIM8 |
| 14 | A0A2P6UZB2 | TPT | Triosephosphate |
| 15 | 731344143 | MOM | Mitochondrial outer membrane protein porin of 36 kDa |
| 16 | P09559 | PRK | Phosphoribulokinase |
| 17 | A0A1U8LRP7/P81760 | TL17 | Thylakoid lumenal 17.4 kDa protein |
| 18 | 731312686 | TL29 | Thylakoid lumenal 29 kDa protein |
| 19 | O24365 | CSP41 | Chloroplast mRNA-binding protein CSP41 |
| 20 | 731365676 | GRIP | GRIP and coiled-coil domain-containing protein C27D7.02c |
| 21 | A0A1D6GF76 | Cytb7 | Cytochrome b-c1 complex subunit 7 |
| 22 | P12355 | Psa-f | Photosystem I reaction center subunit III |
| 23 | D7KW69 | Fd-1 | Ferredoxin |
| 24 | A0A0J8E3S1 | Fd | Ferredoxin |
| 25 | Q8MC96 | ATP synthase | ATP synthase epsilon chain |
| 26 | G1E6K5 | CA | Carbonic anhydrase |
| 27 | P10871 | RuBisCO | Ribulose bisphosphate carboxylase/oxygenase activase |
| 28 | A0A061F296 | RuBP | Rubredoxin-like superfamily protein |
| 29 | A0A0K9QU20 | FBA | Fructose-bisphosphate aldolase |
| 30 | O20252 | SBPase | Sedoheptulose-1,7-bisphosphatase |
| 31 | O24360 | CP12 | calvin cycle protein CP12 |

^a^ Protein ID, gi number of NCBI;
