# Supplementary material for: Cys-SH based quantitative redox proteomics of salt induced response in sugar beet monosomic addition line M14
Source: Bot Stud. 2021 Oct 18;62:16. doi: 10.1186/s40529-021-00320-x (PMC8523603; doi:10.1186/s40529-021-00320-x)

Supplemental Table S7. MS/MS spectra showing redox modified cysteine sites.

G1E6K5：FMVFACSDSR


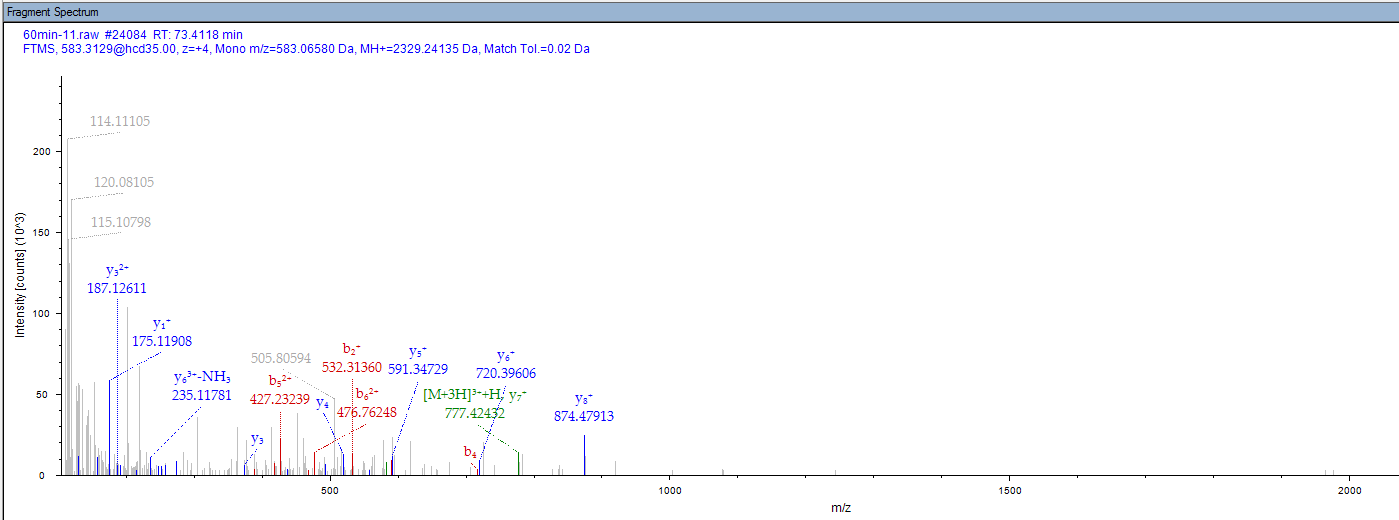

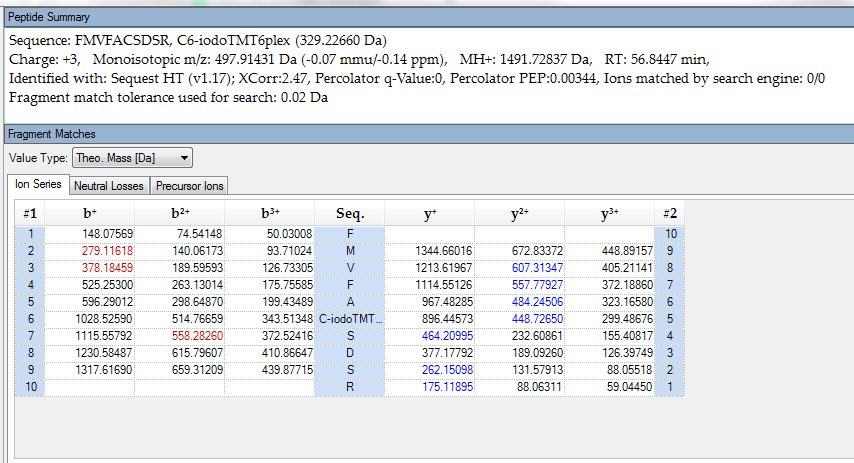


A0A0K9RGC9：YWPTFGPQCNLYVPAPLLR


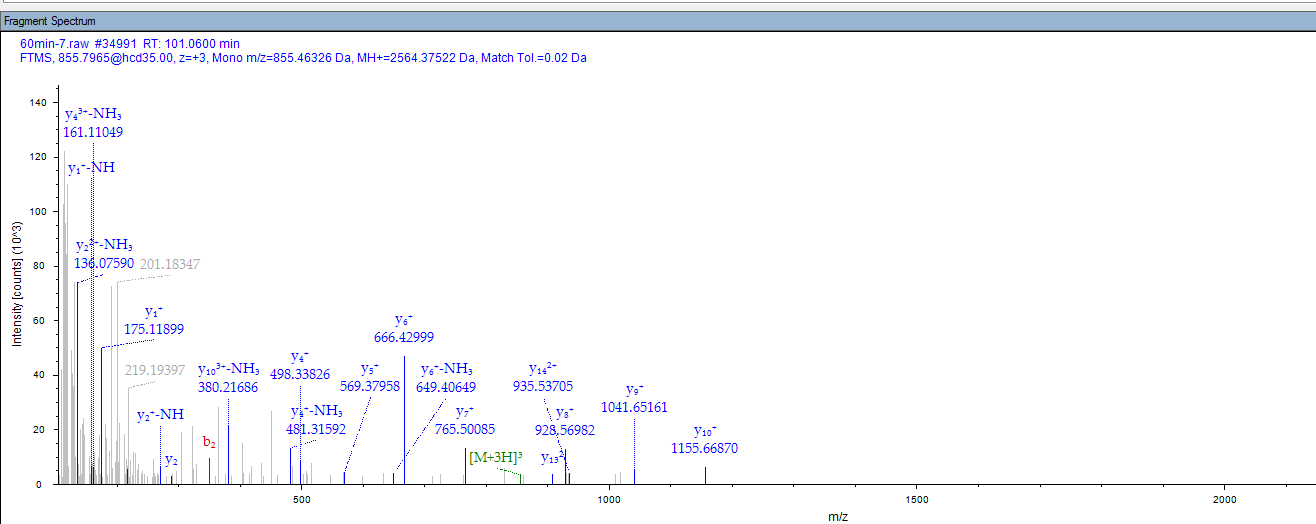

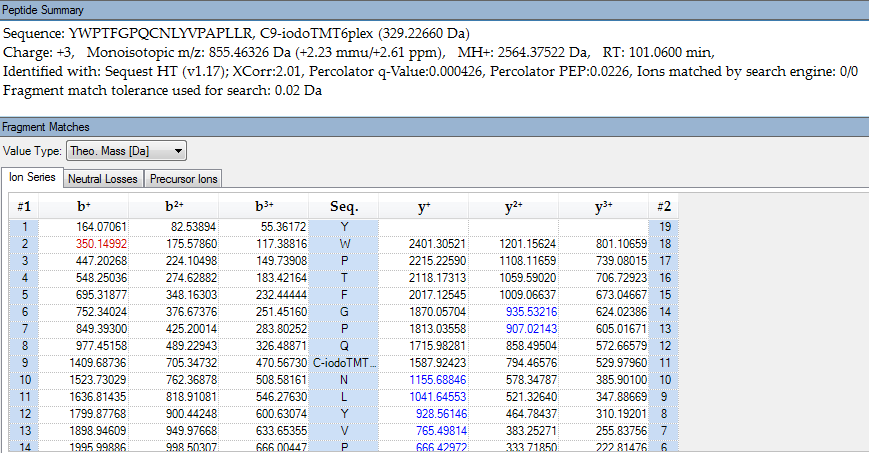


A0A1S3CE63：LVSLSEQQLVDCDHECDPEER


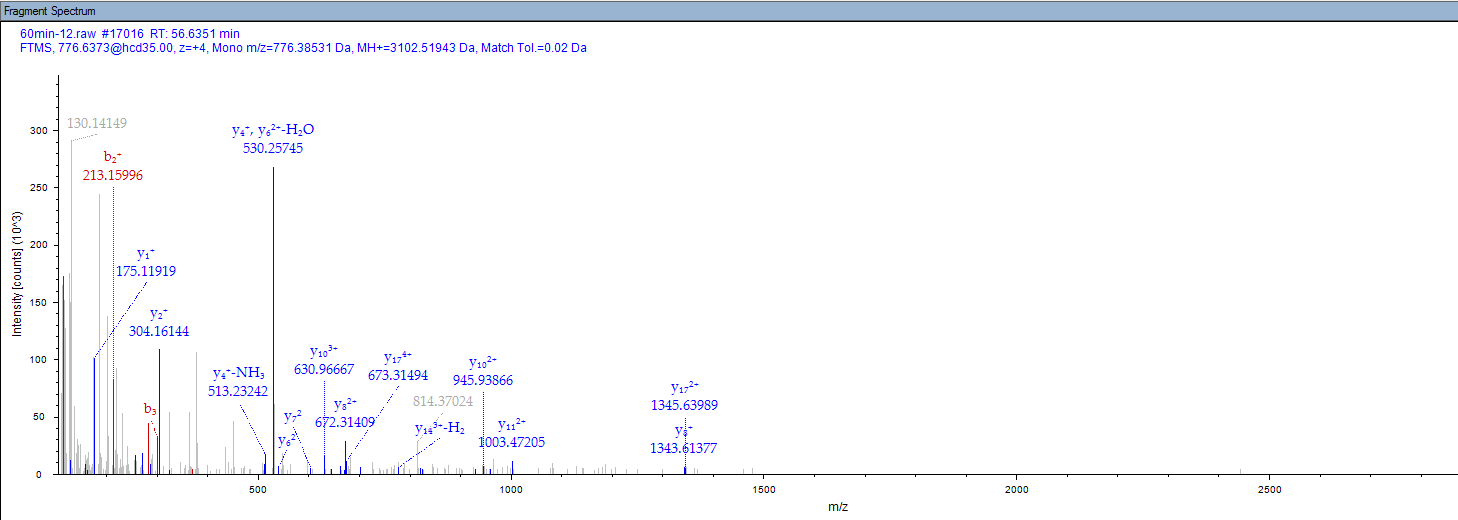

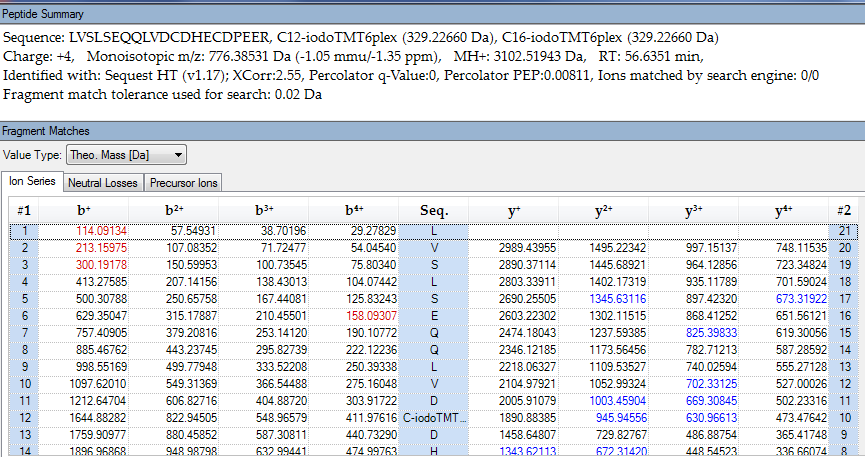


A0A2P6UZB2：VIACVGETLEQR


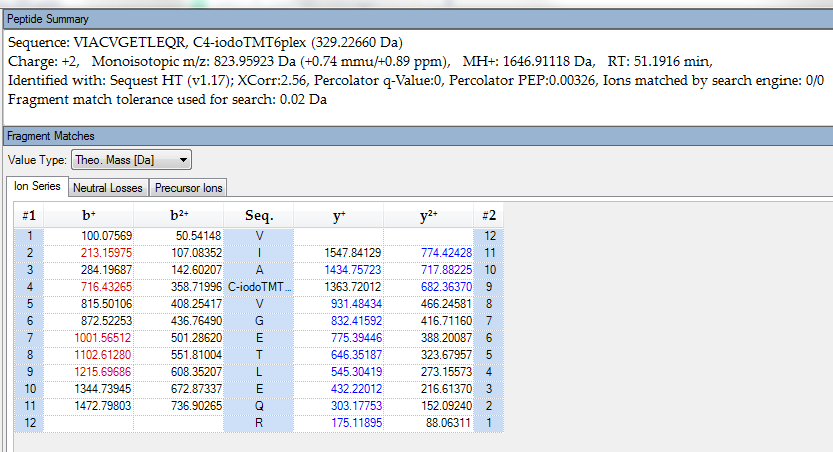

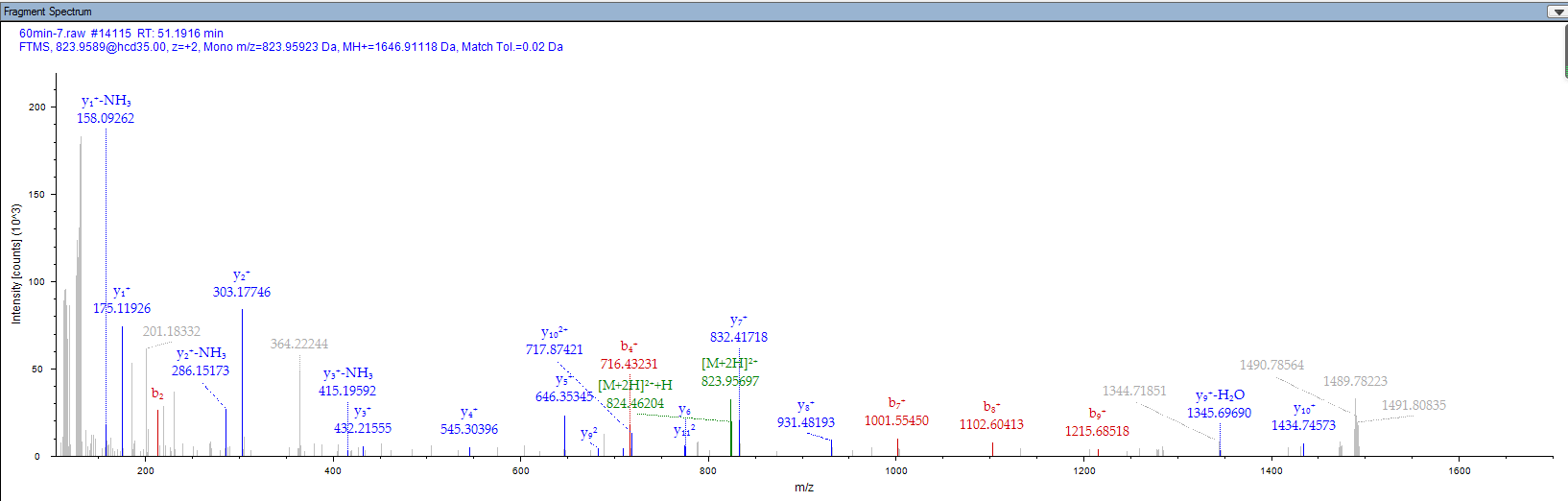


A0A0K9RNM7:CGVSIPGPVGPQADCSQIH


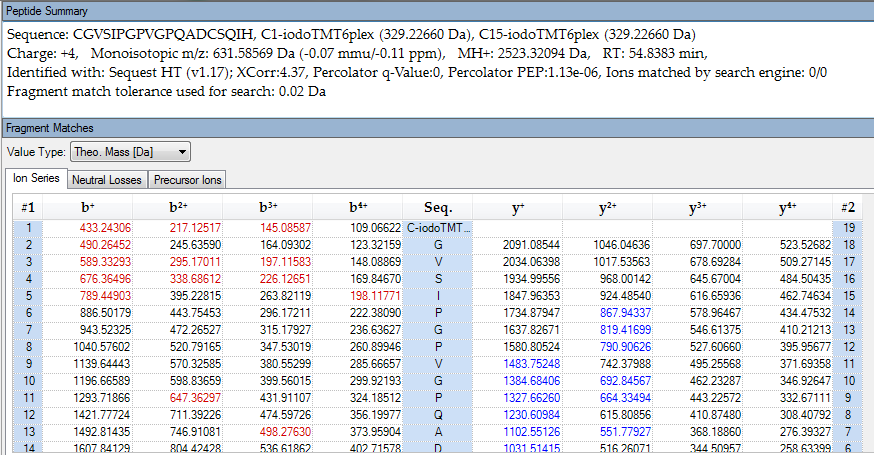

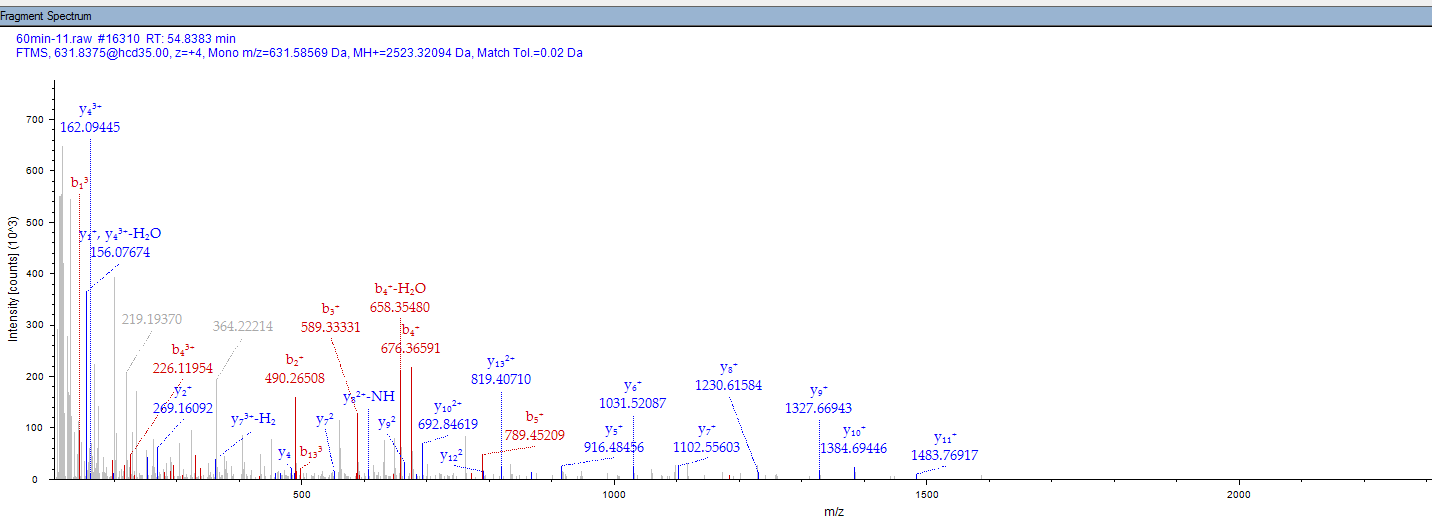


LPPLSTEPNRCER

A0A1S2XUR4:VLVTGTGPLGCVPGELASQGSQNGECAPEPQR


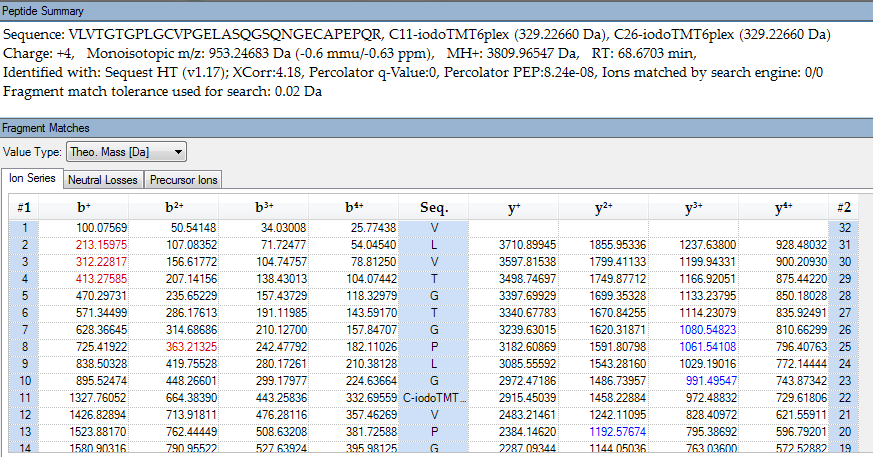

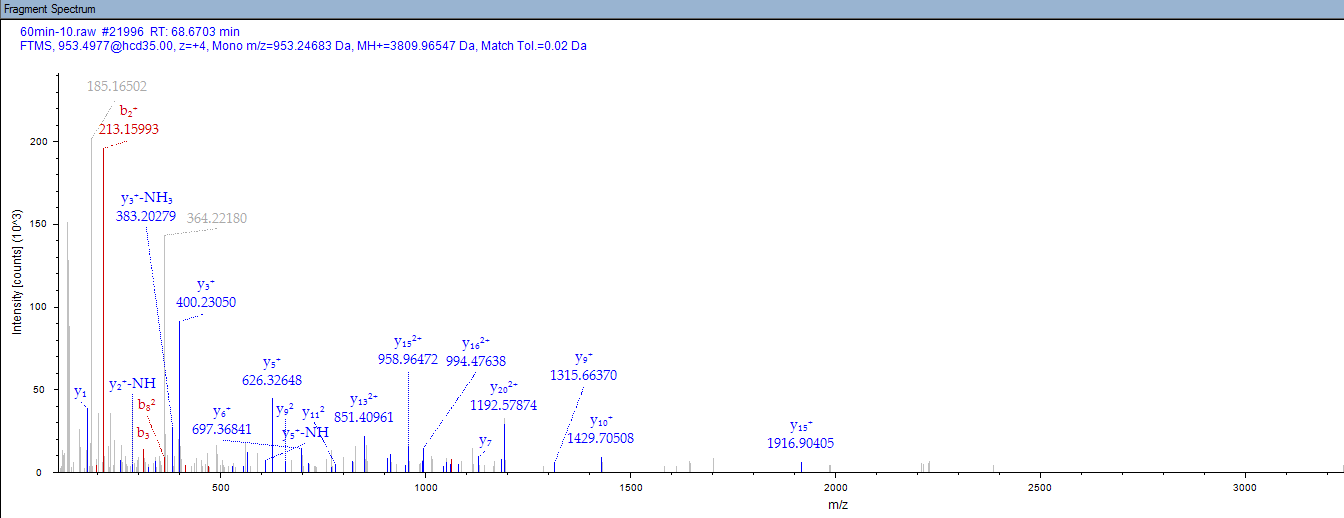


A0A1U7ZGK1:FSSSEATCLNNCAQR


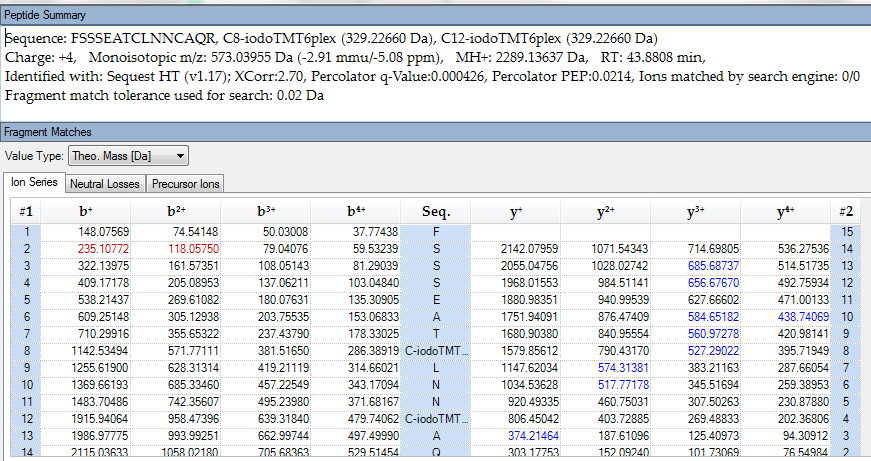

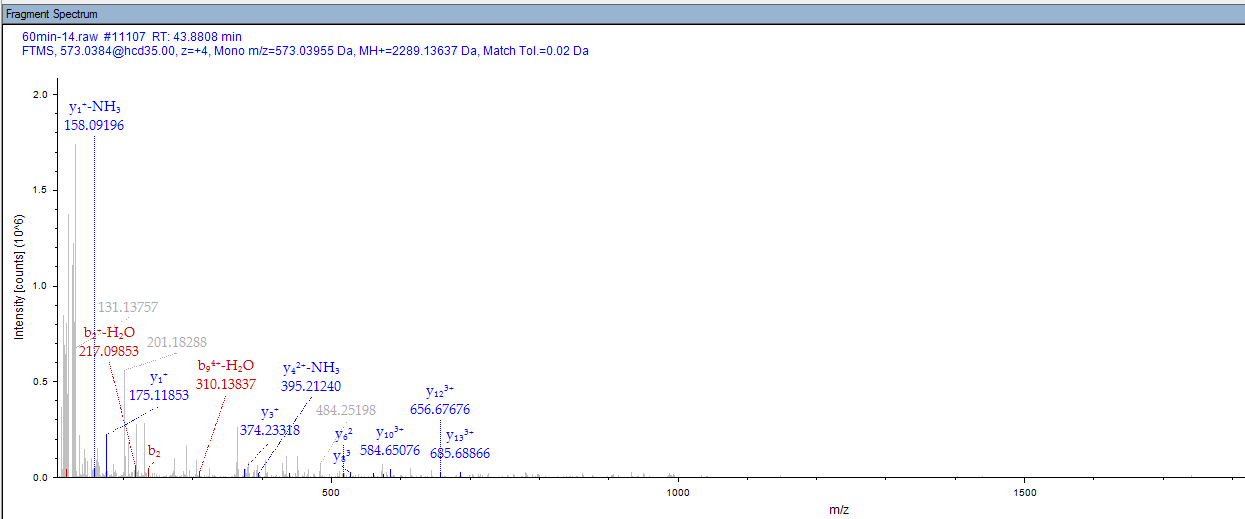


P81760:LPPLSTEPNRCER


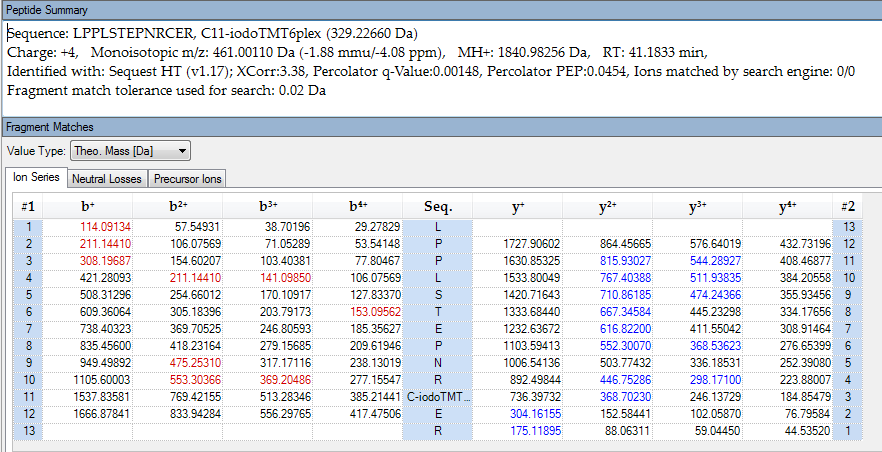

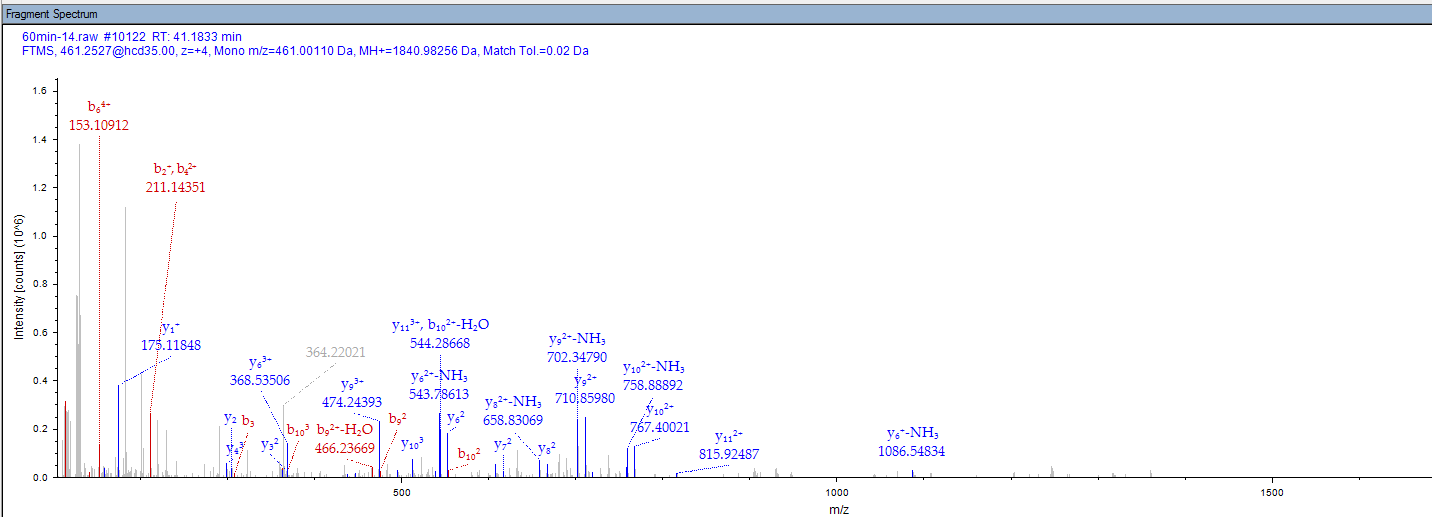


A0A314V1F4:ISFCVDNTASNLIECPR


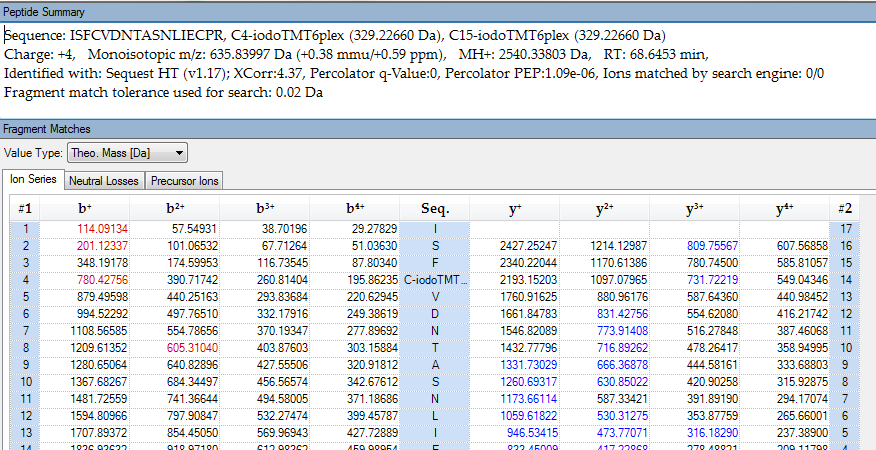

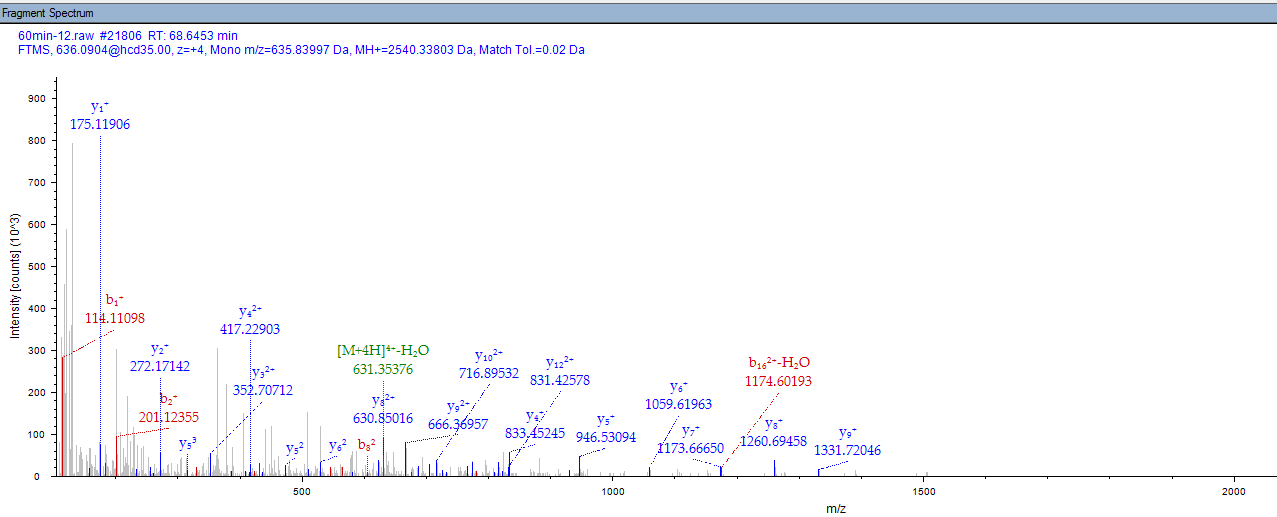


A0A314V1F4:NAIEGGVGFTPAIGCNVDPAGTTQLYR


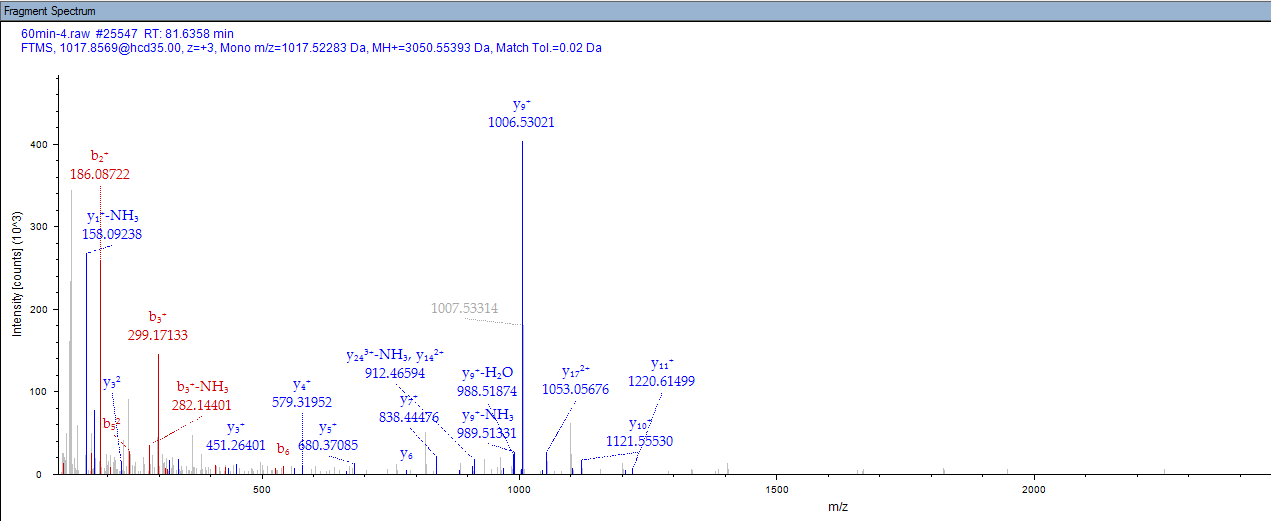

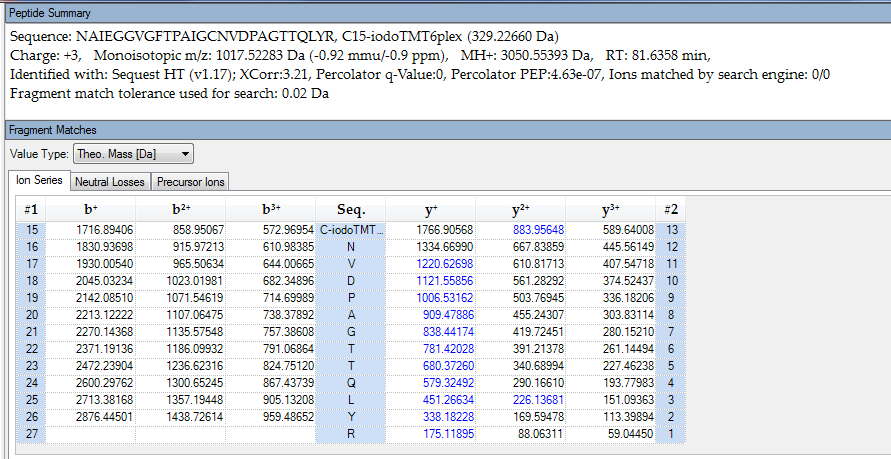


C0Z387:CFVGGLAWATDDR


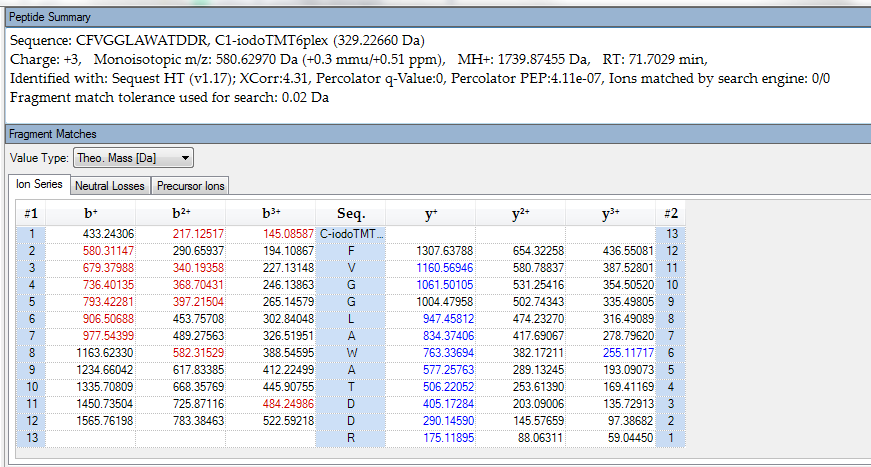

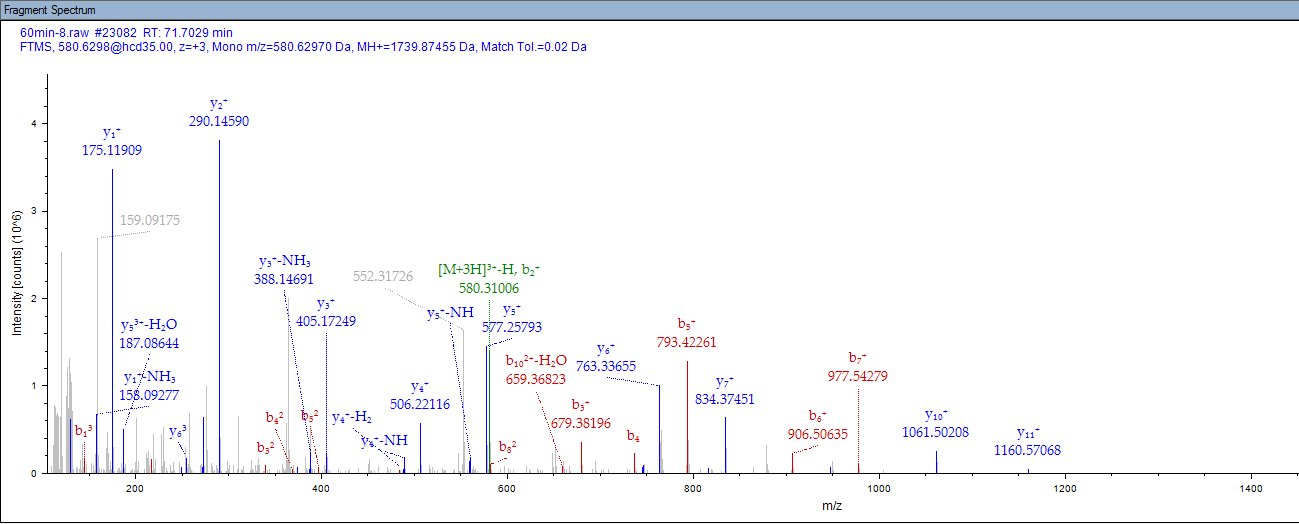


A0A0K9QZ36：QYDCELLIR


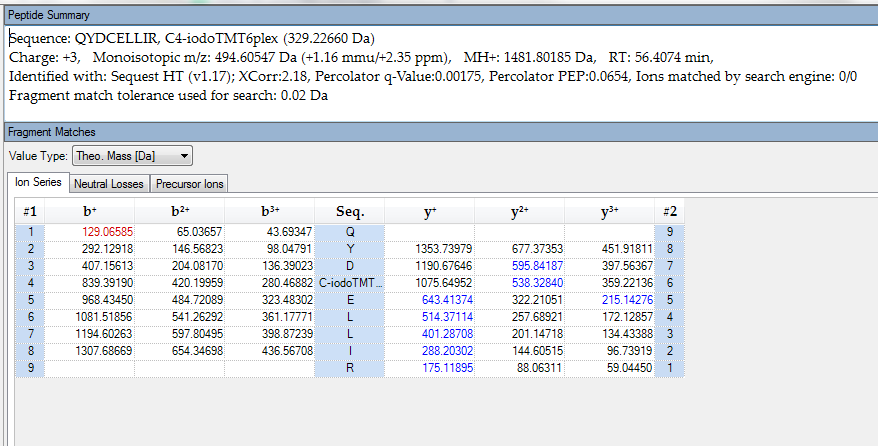

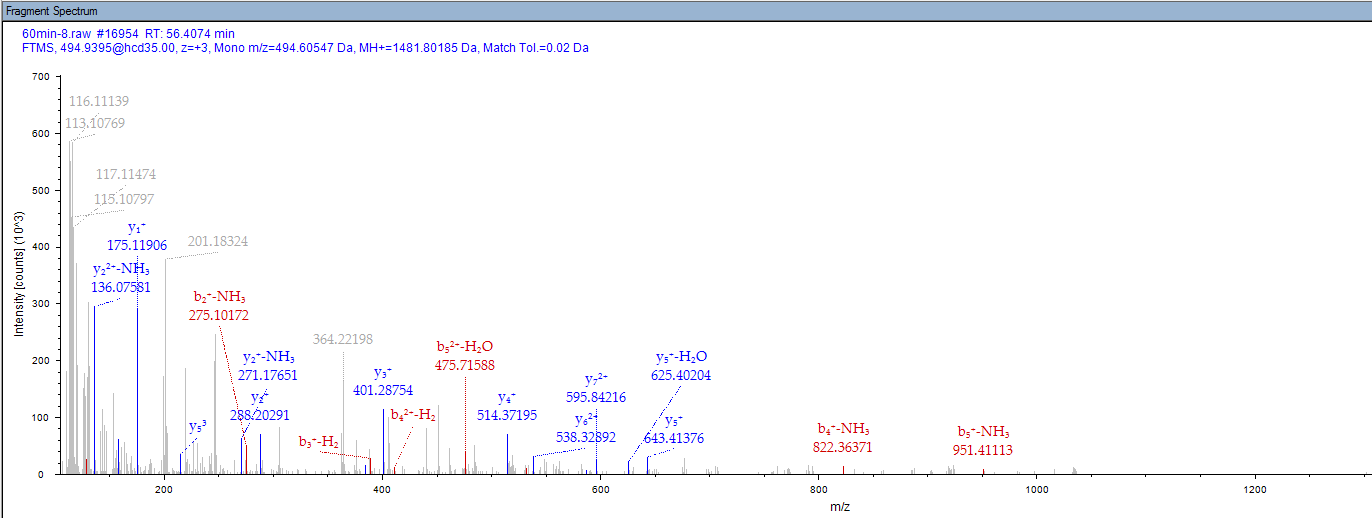


A0A0K9S0G4：IEYYATTAEPSCELNVVR


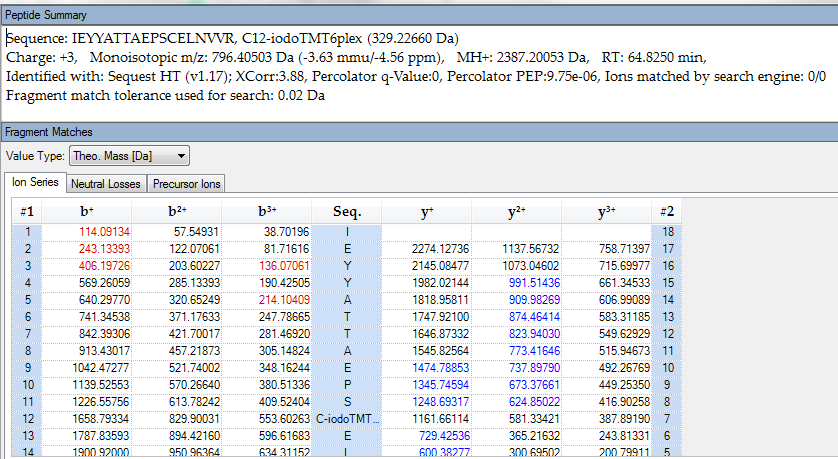

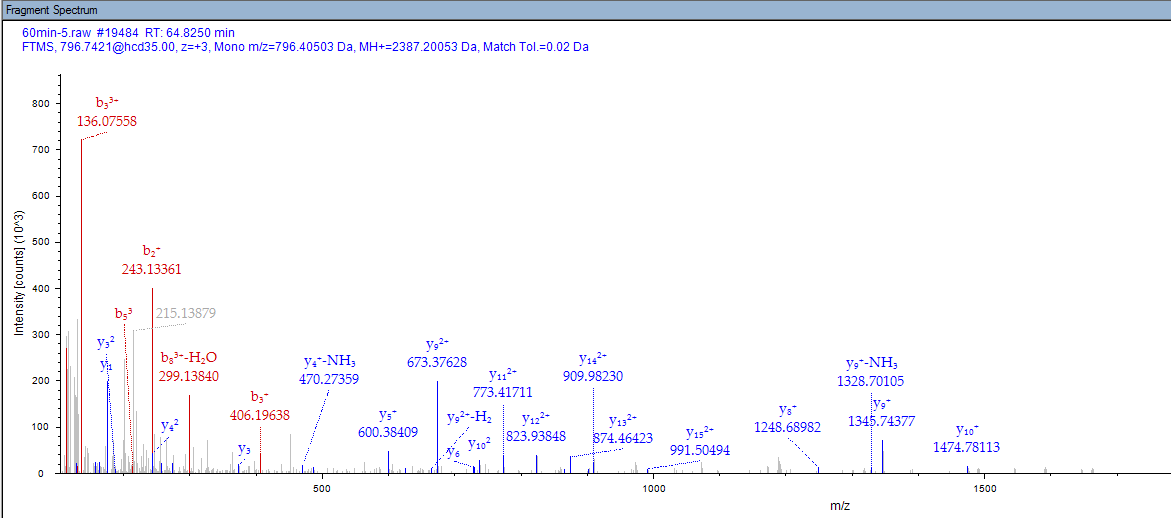


A0A0K9S0G4：SGLAYCDLVVGSGVPAPYNTLINVHYTAR


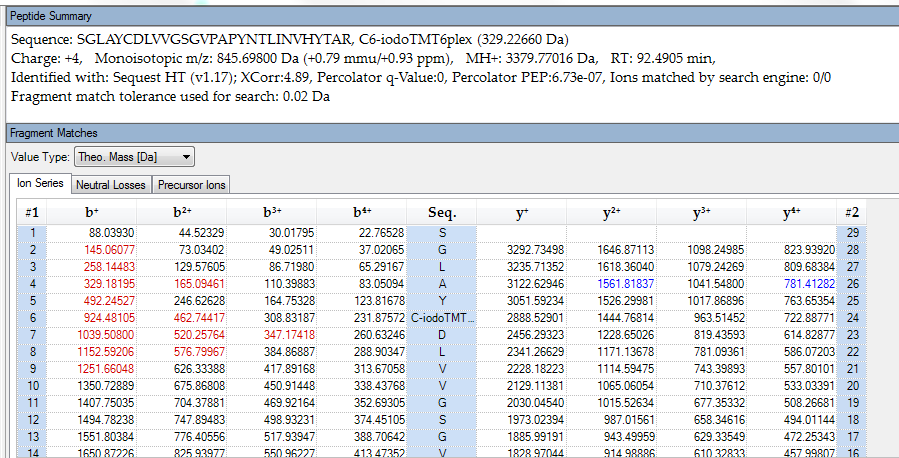

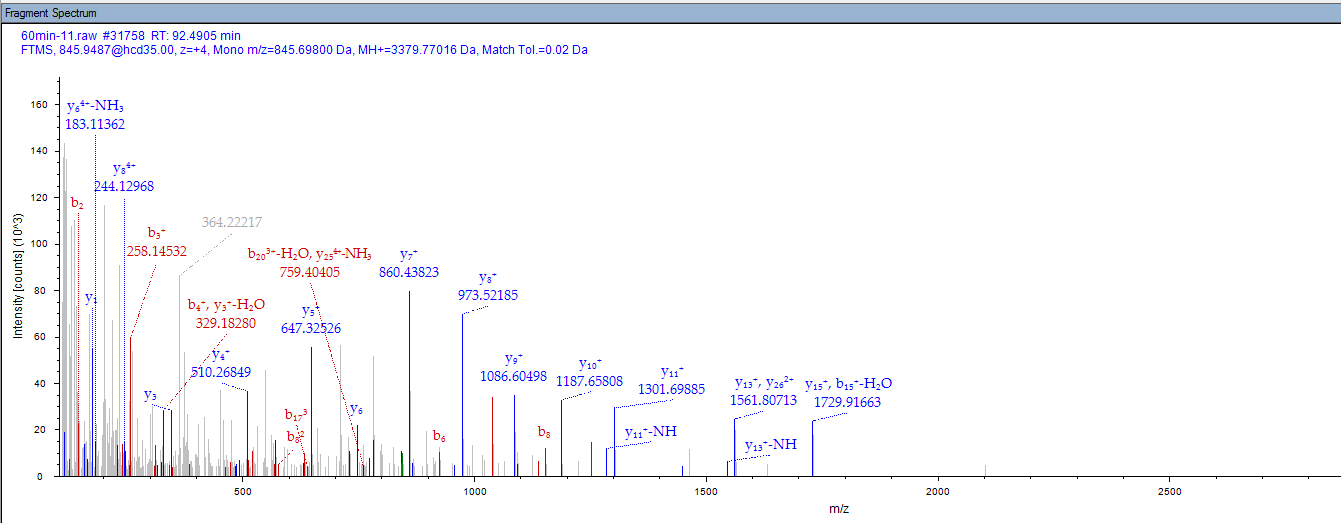


A0A1J3HHY8：FVNSGTEACMGVIR


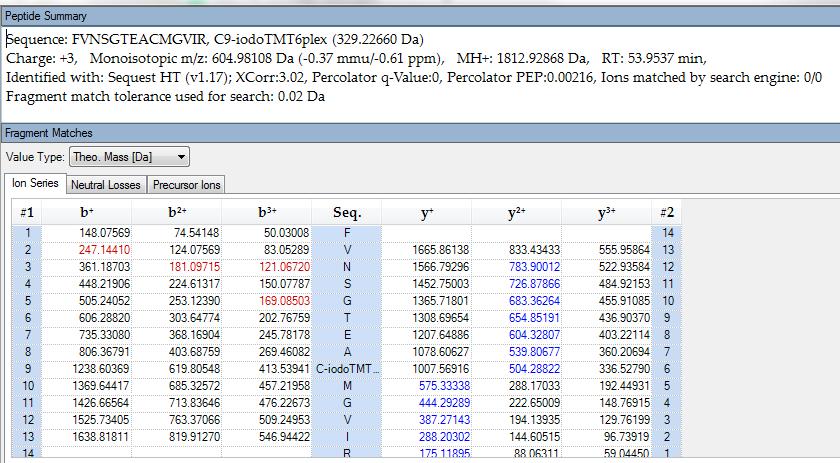

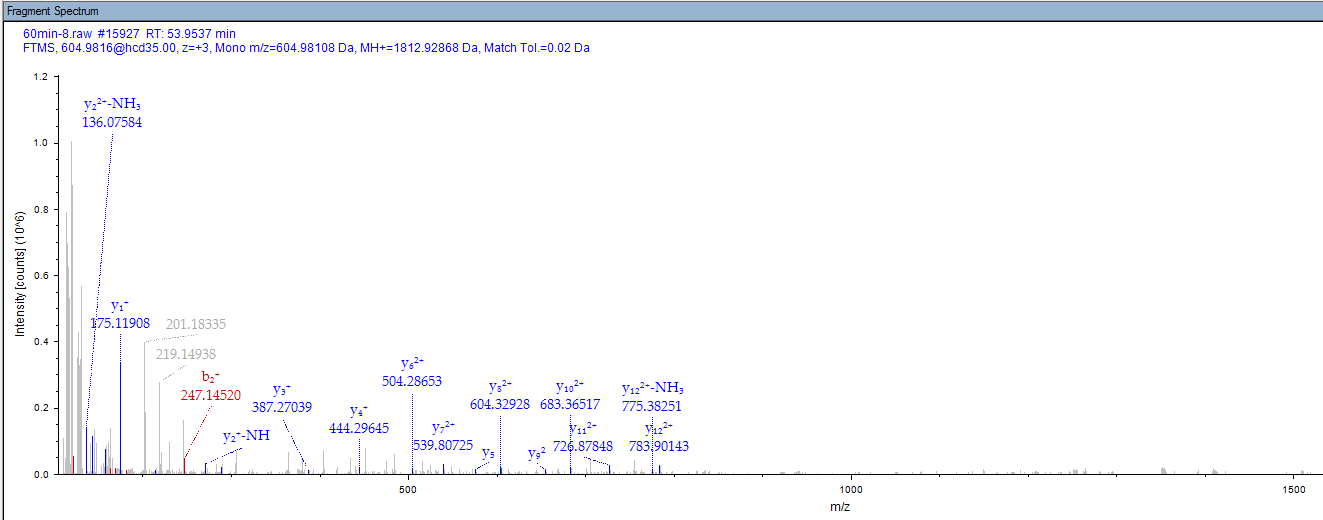


O24365：LCAQATGR


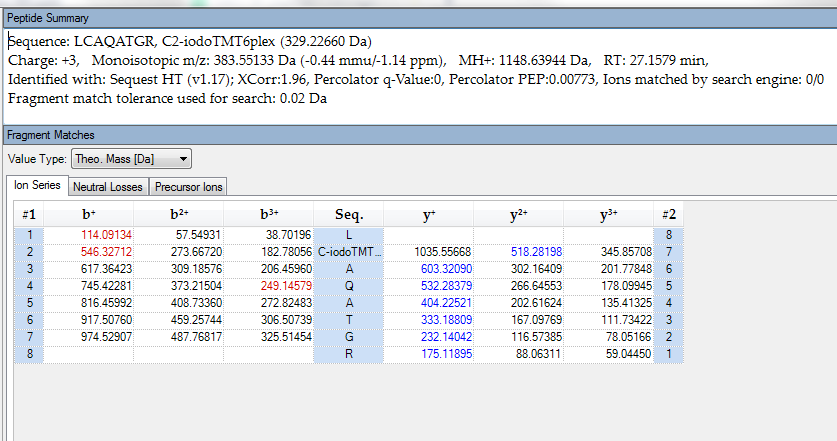

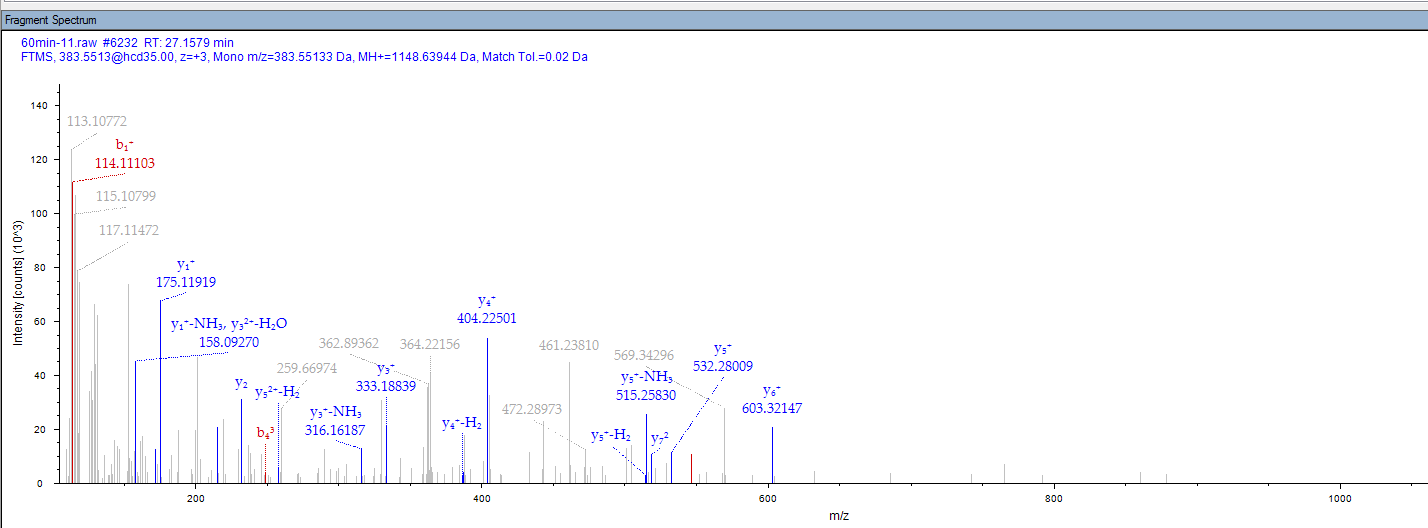


A0A1D1Y6P4
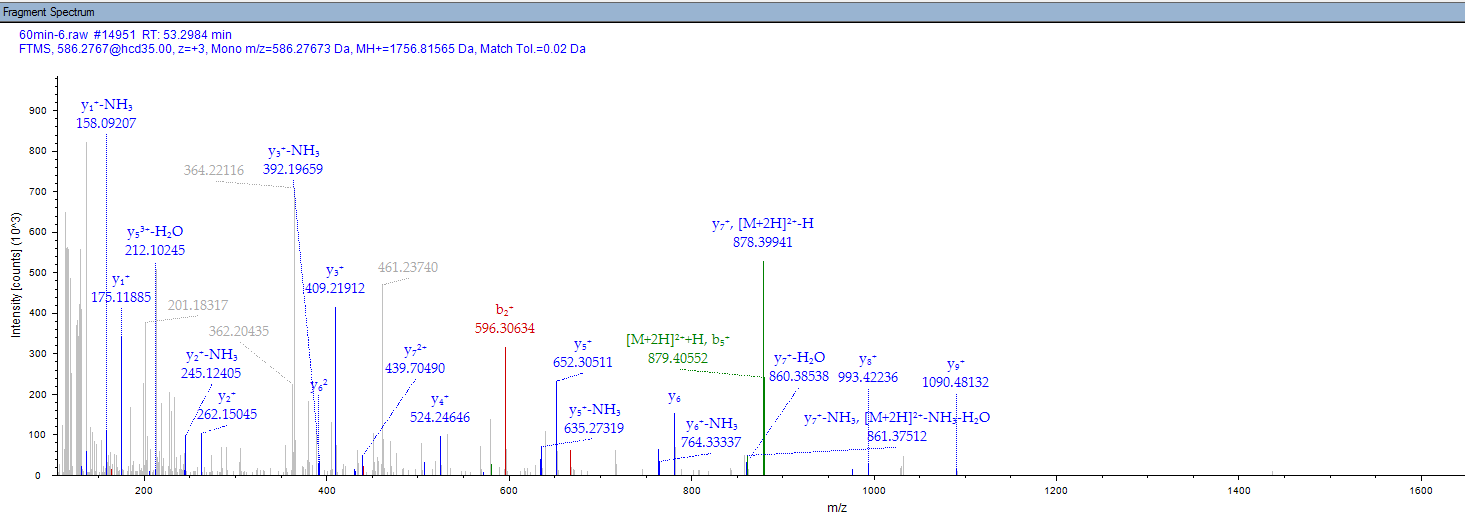

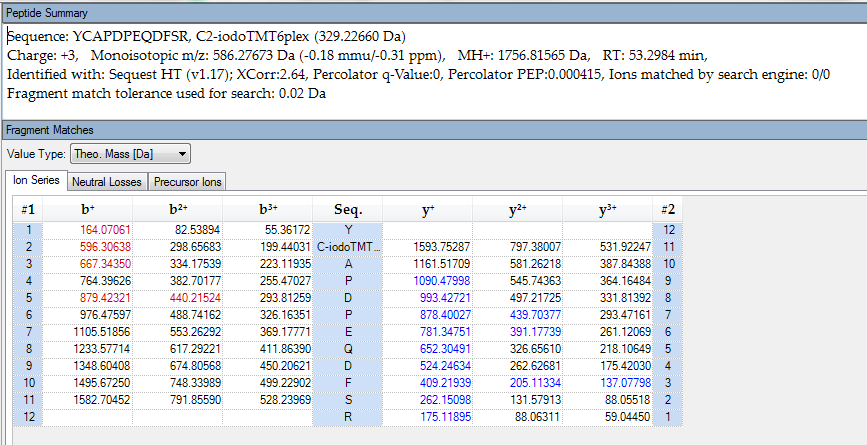
：YCAPDPEQDFSR

A0A161DY72：SDDFSSLCGPVIDDVR


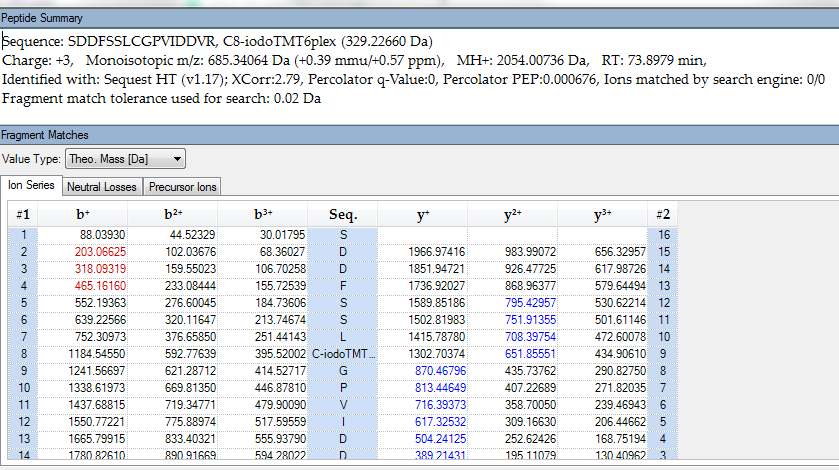

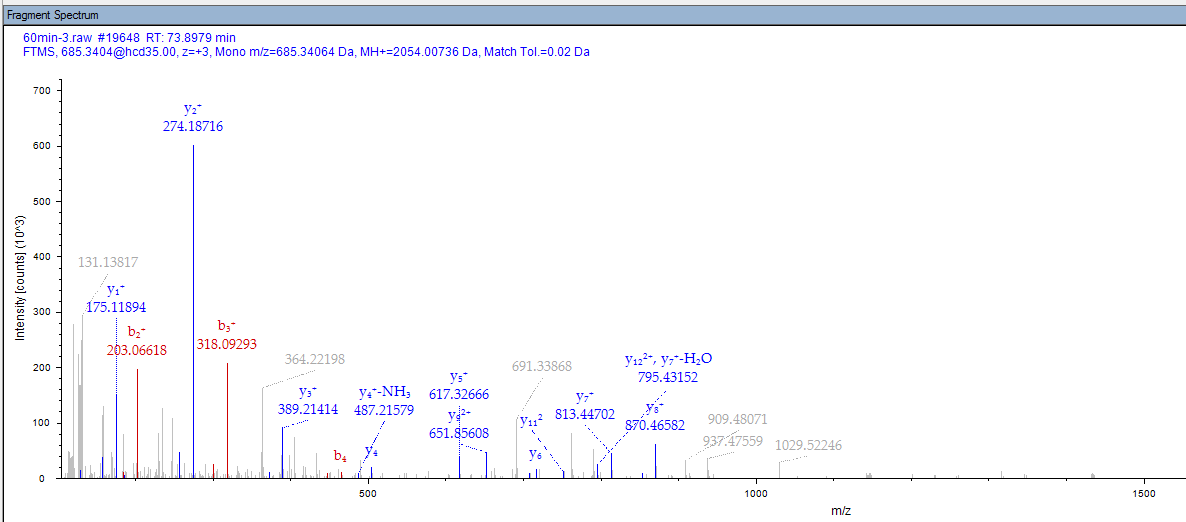


A0A161DY72：VAEIMIHNPGVEEDPACGPLIDSVAMR


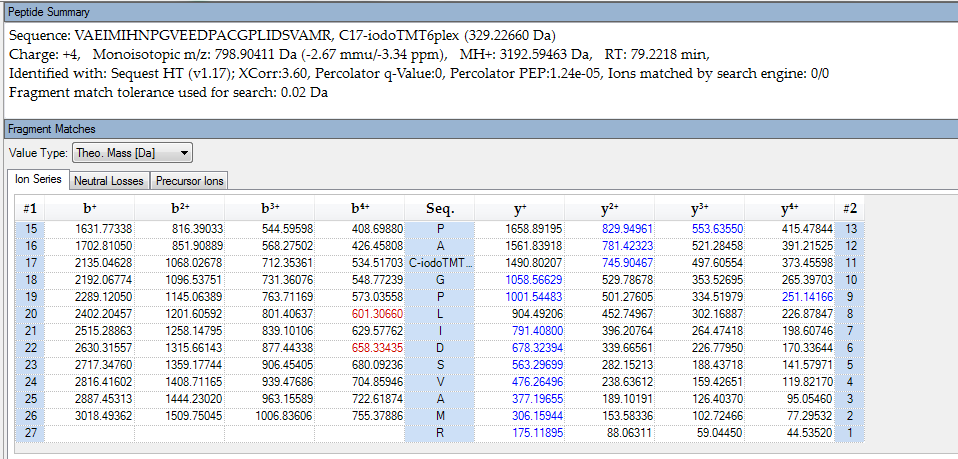

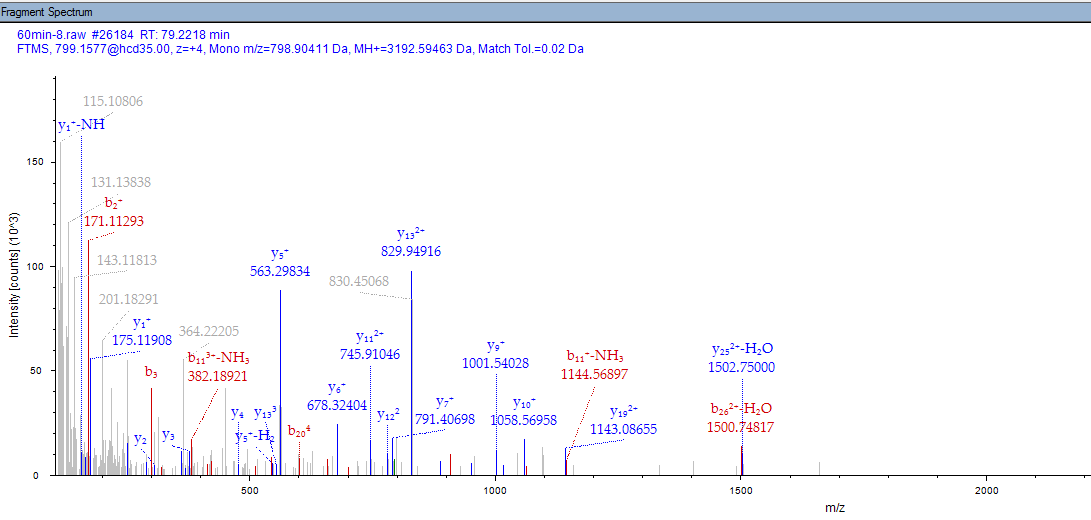


A0A0K9QD73：TGQALVIGLYDEPVTPGQCNMIVER


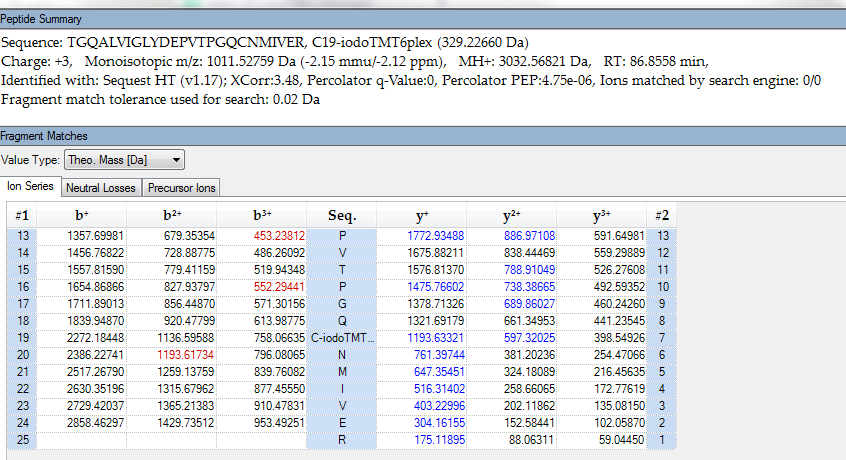

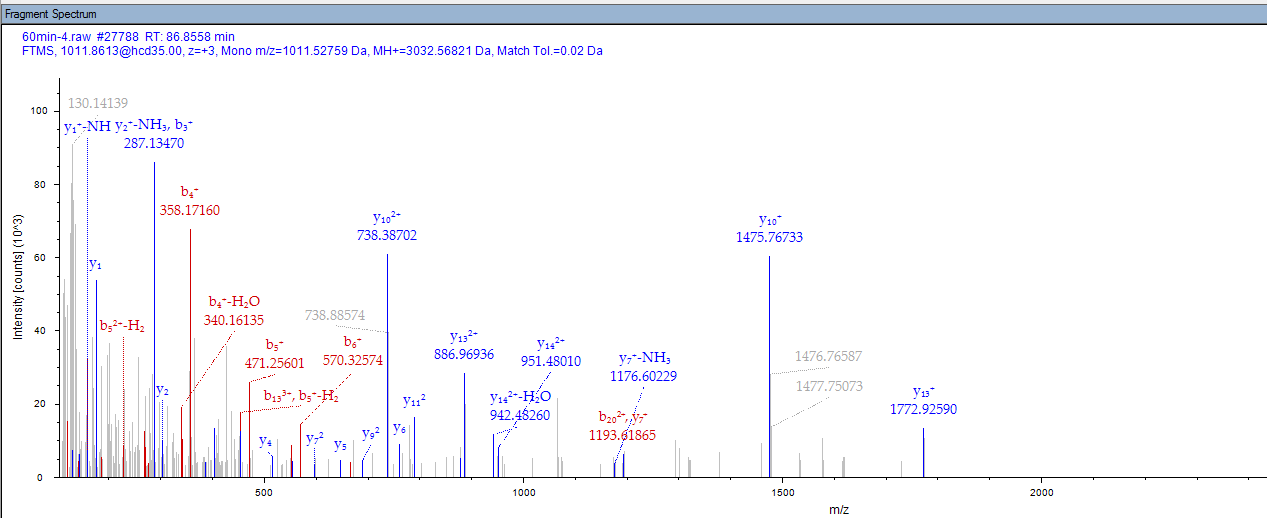


A0A061F296：FAVLNTGIYECR


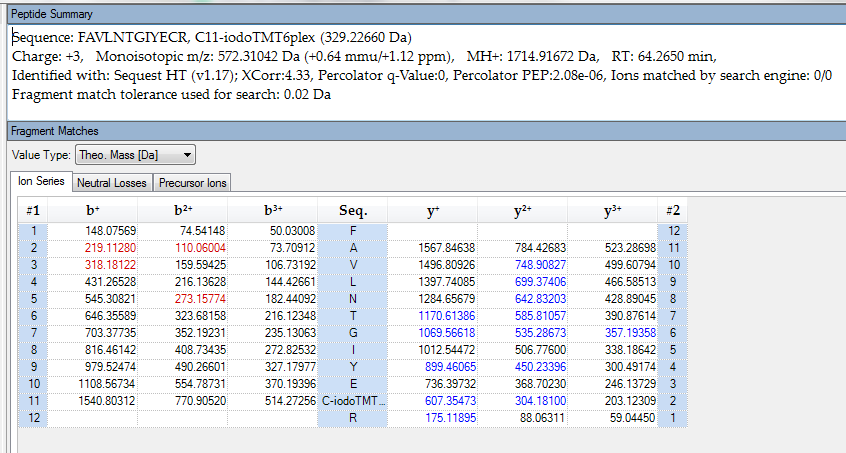

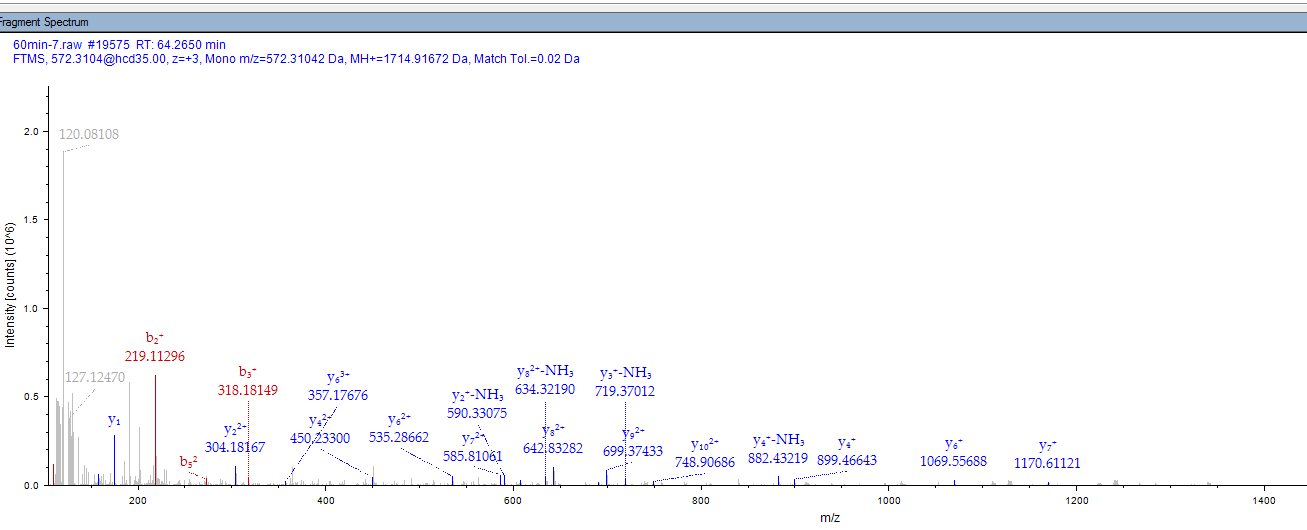


A0A0K9QU20：TVVSVPCGPSALAVKEAAWGLAR


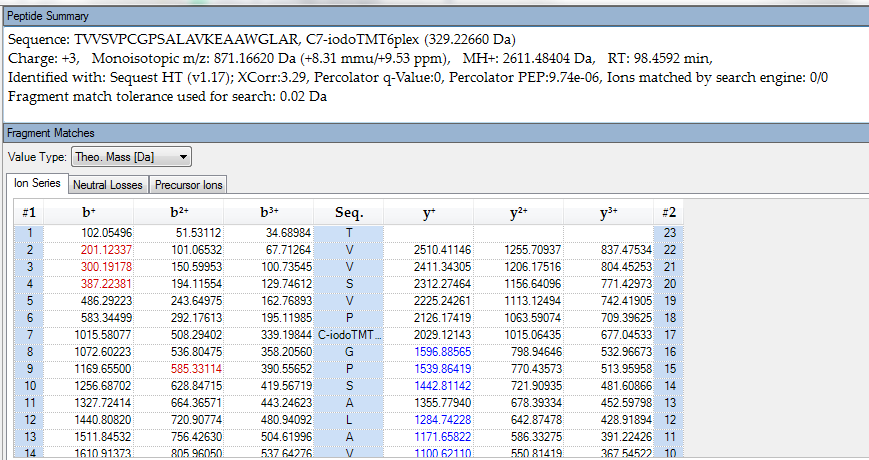

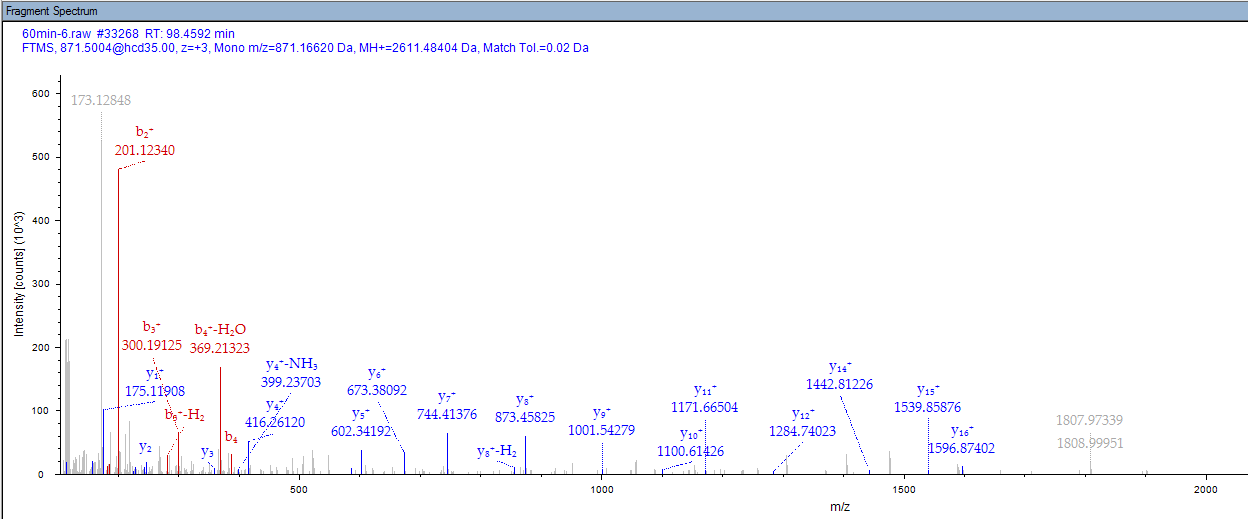


A0A1U7XAS5：CEYELVMER


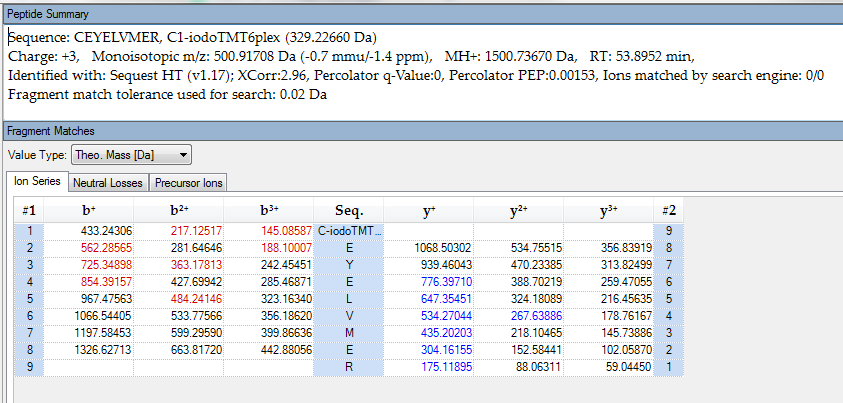

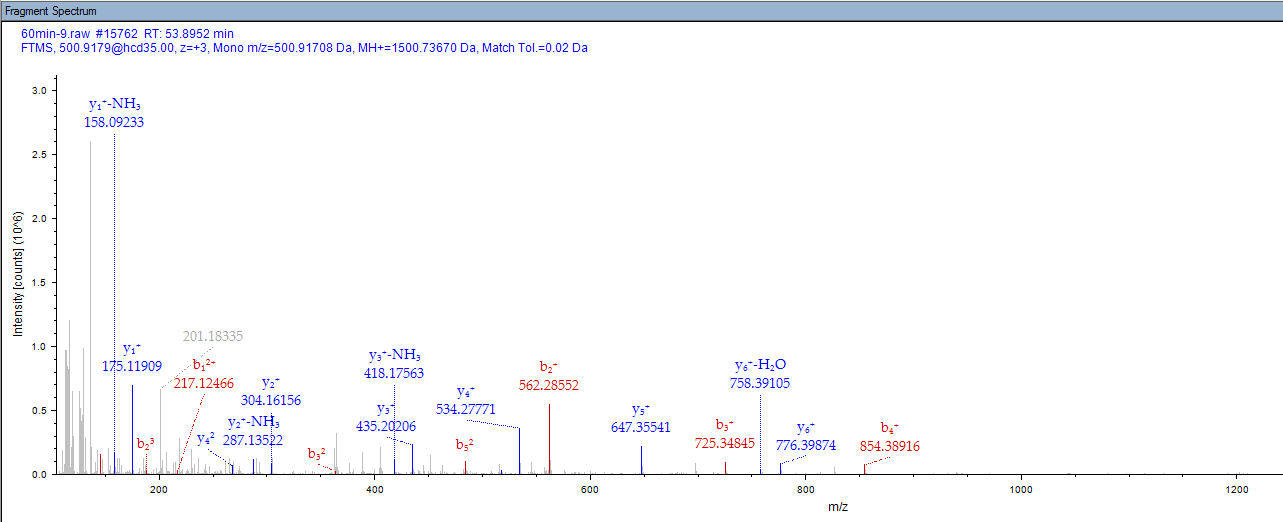


A0A1U8EH95：KGNMCVLFINDLDAGAGR


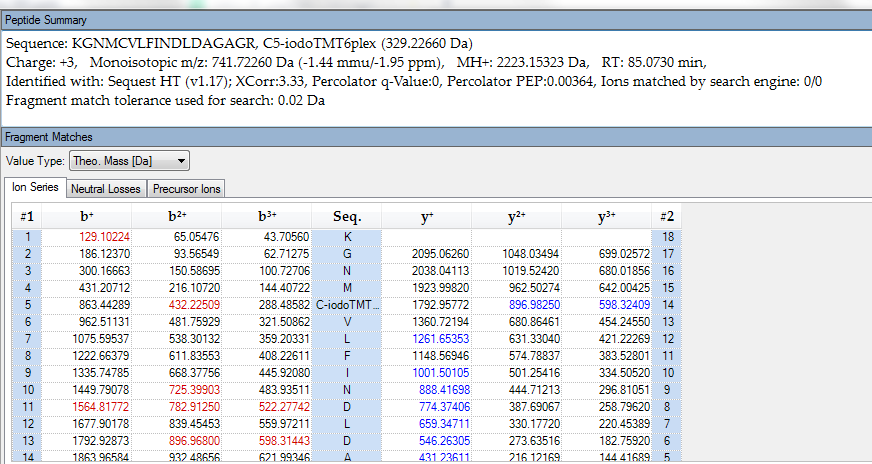

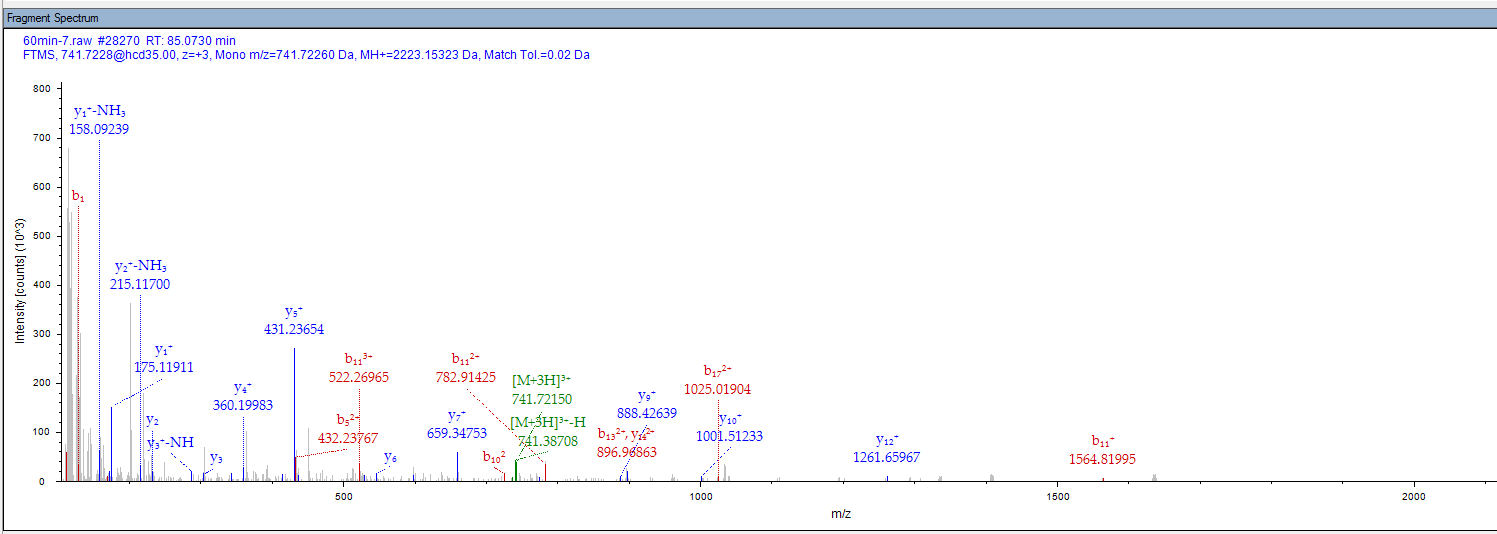


E5GBR8：FAVLNTGIYECR


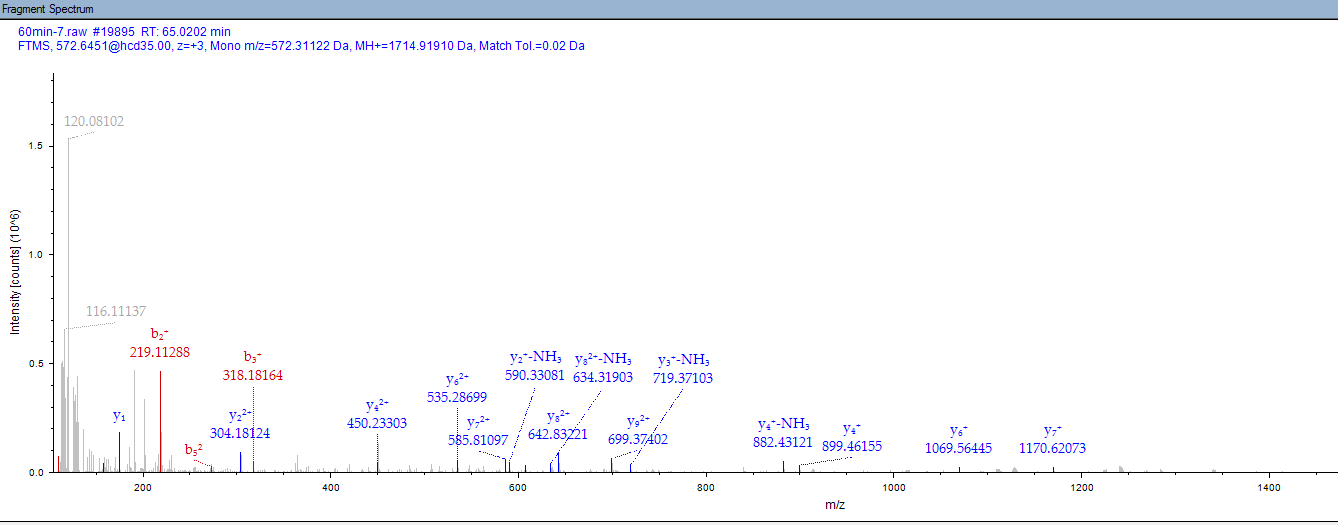

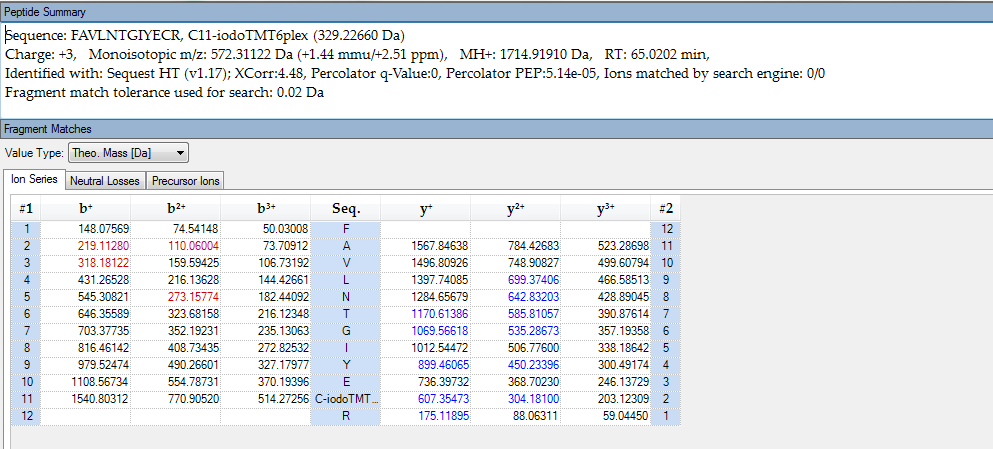


O20252：LFCPGNLR


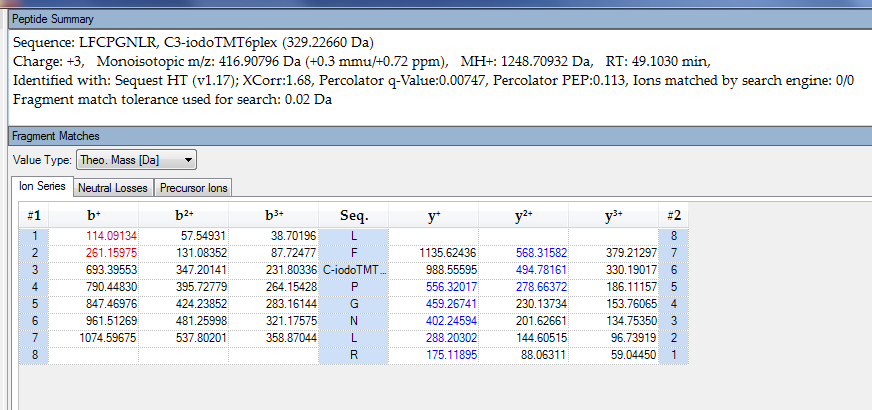

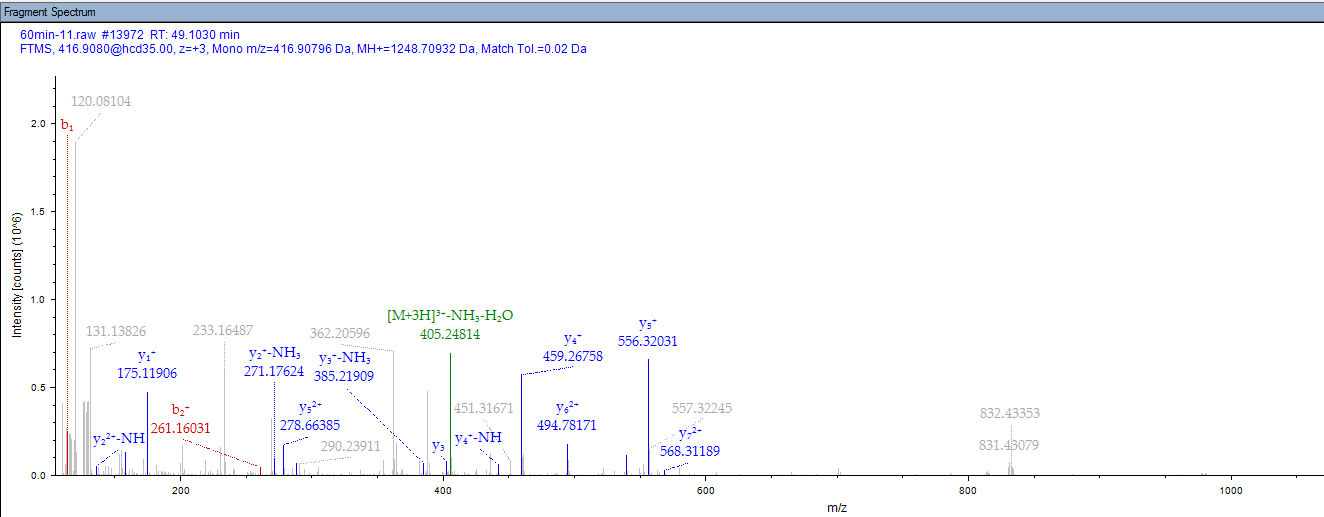


O24360：KEAQEMCSDDPASGECVAVWDEVEELSAAASHAR


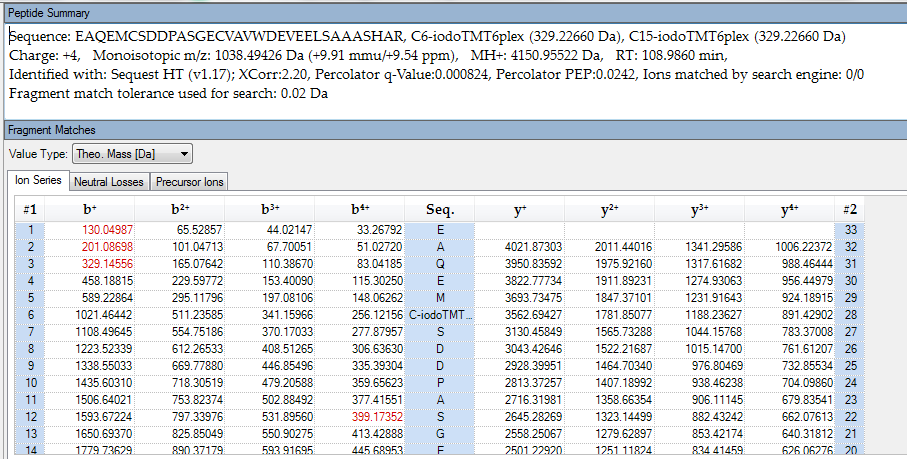

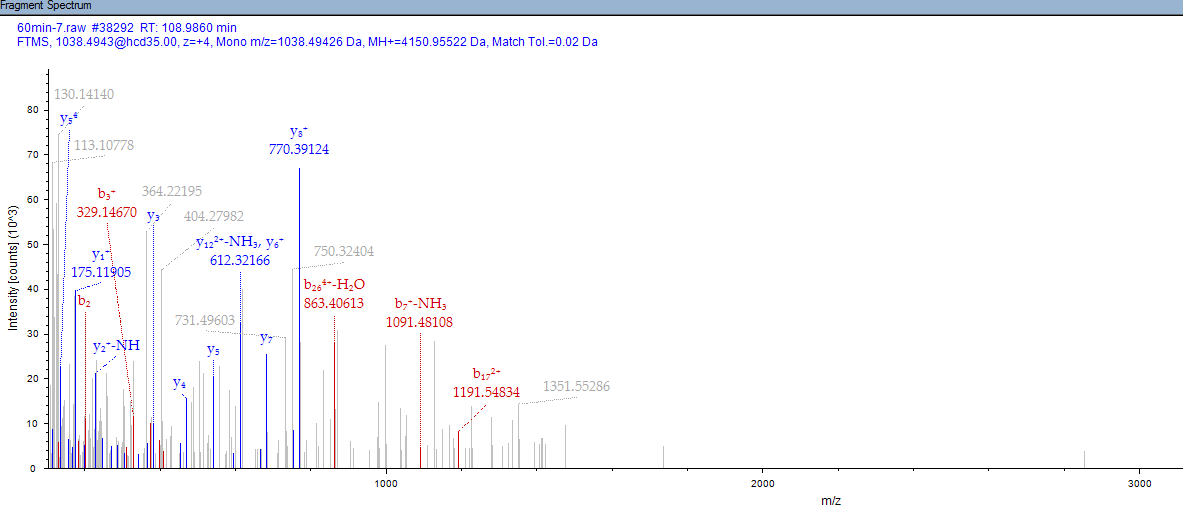


P09559：FFNPVYLFDEGSTISWIPCGR


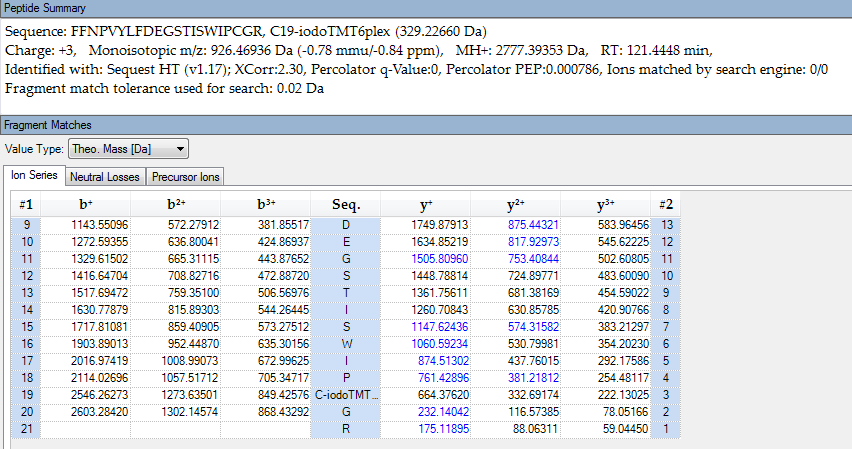

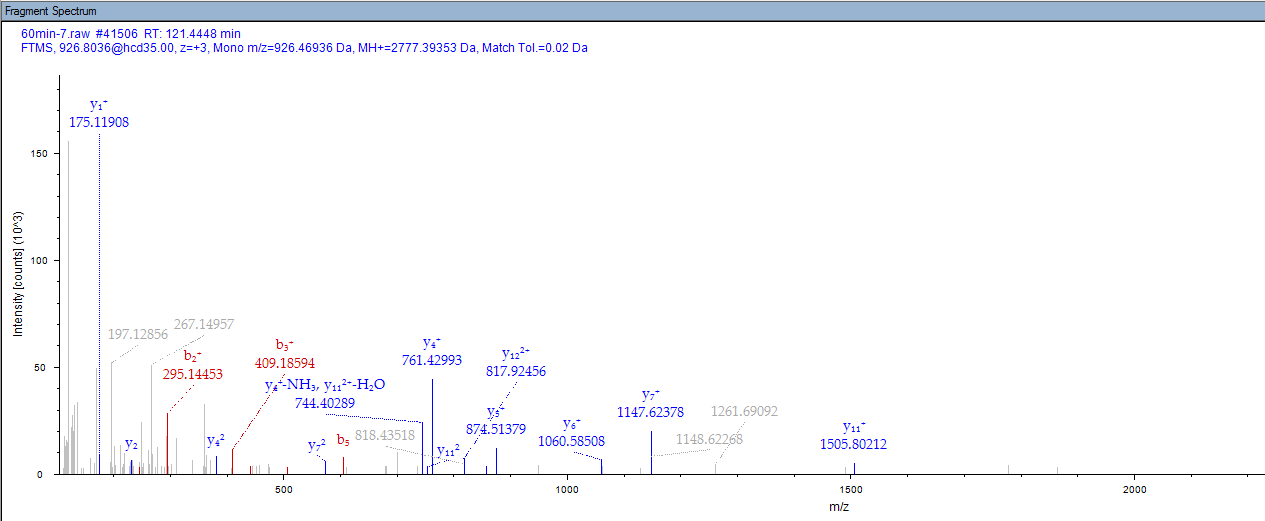


P12355：FENYGNYGLLCGSDGLPHLIVSGDQR


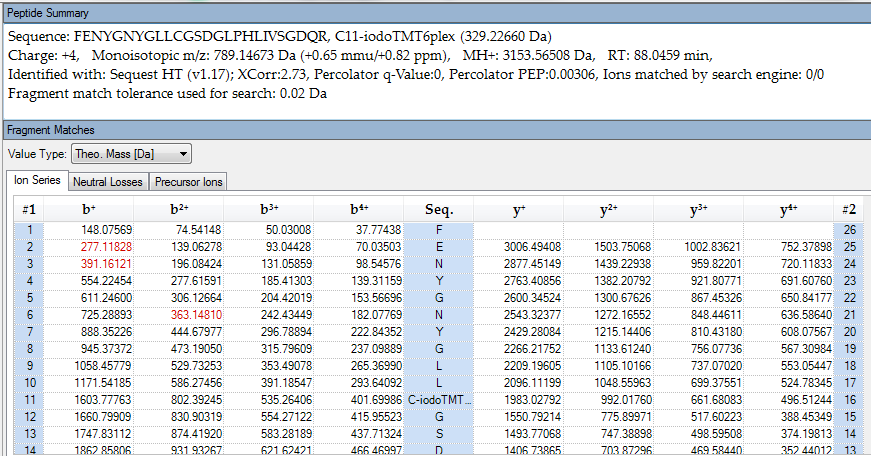

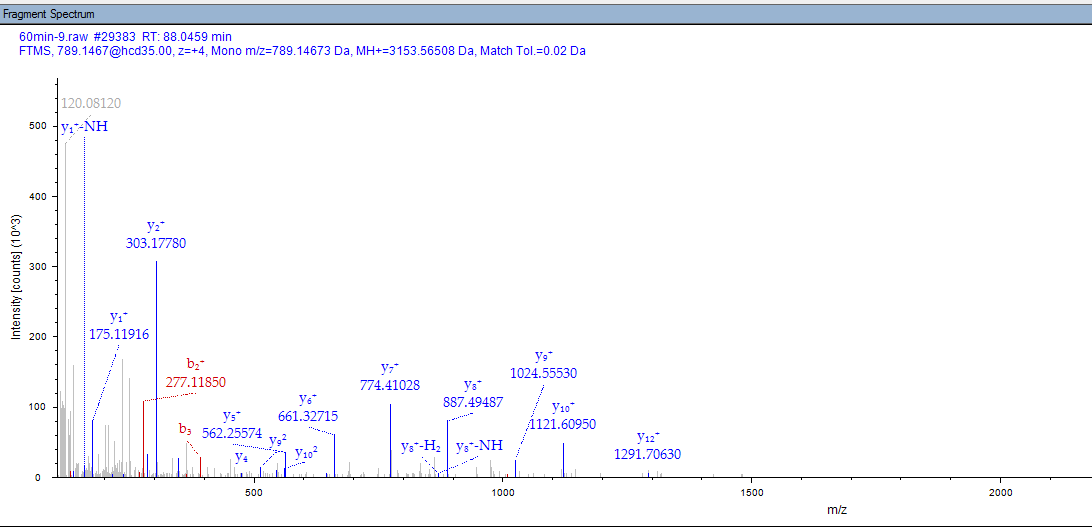


P10871：IGVCTGIFR


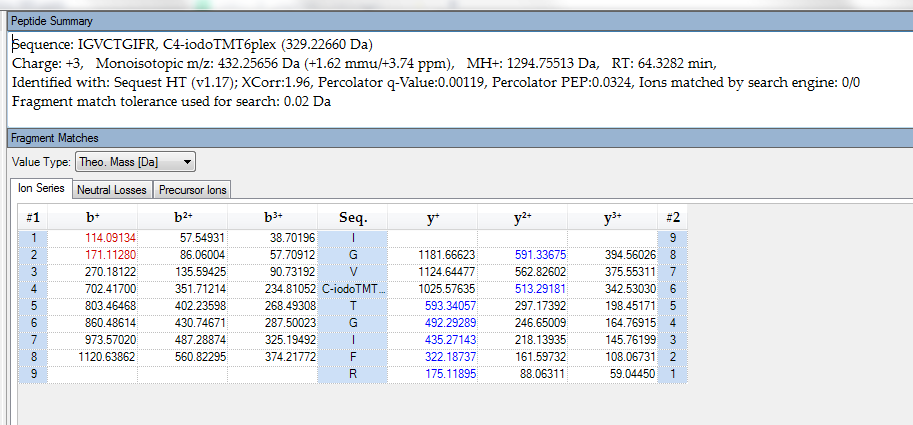

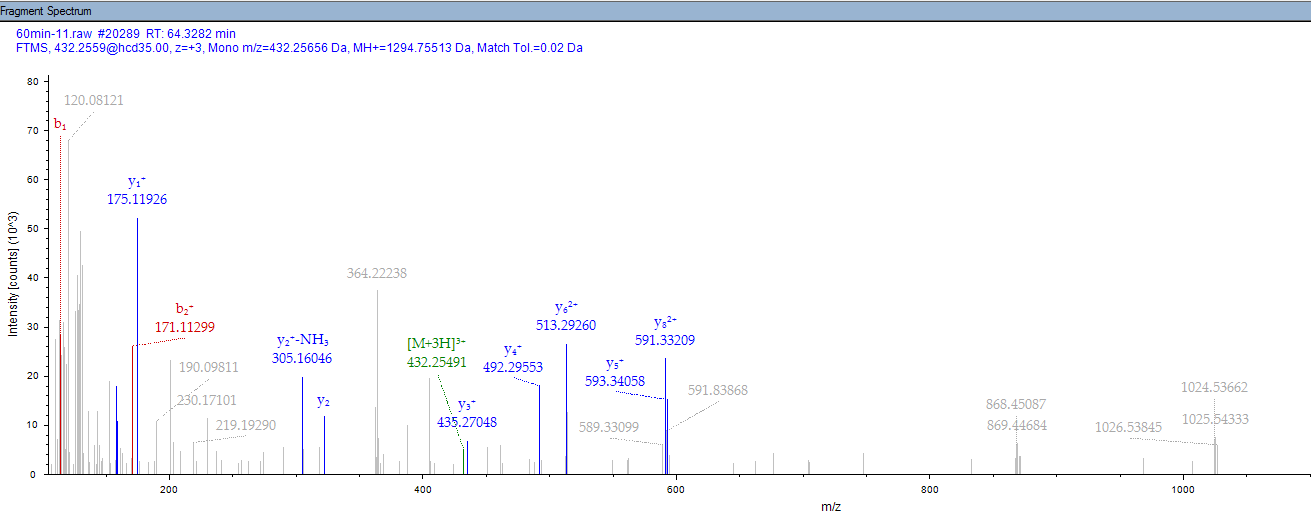


A0A0K9QDU1：NSFYASTCPGVEGIVR


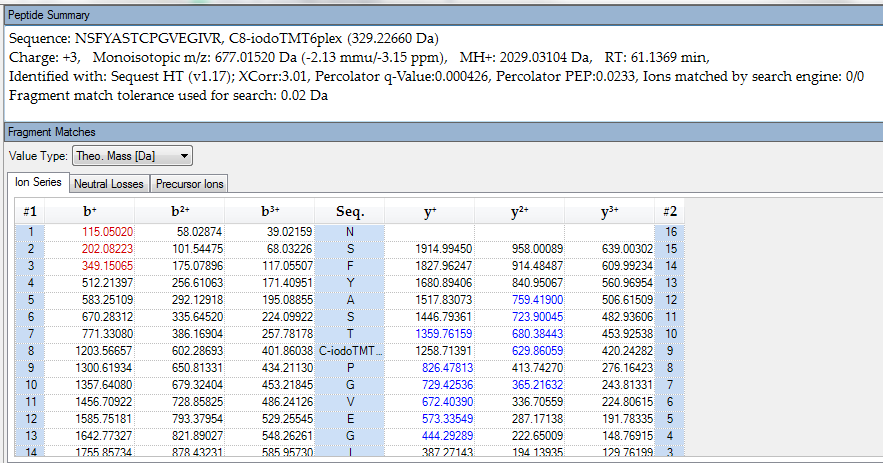

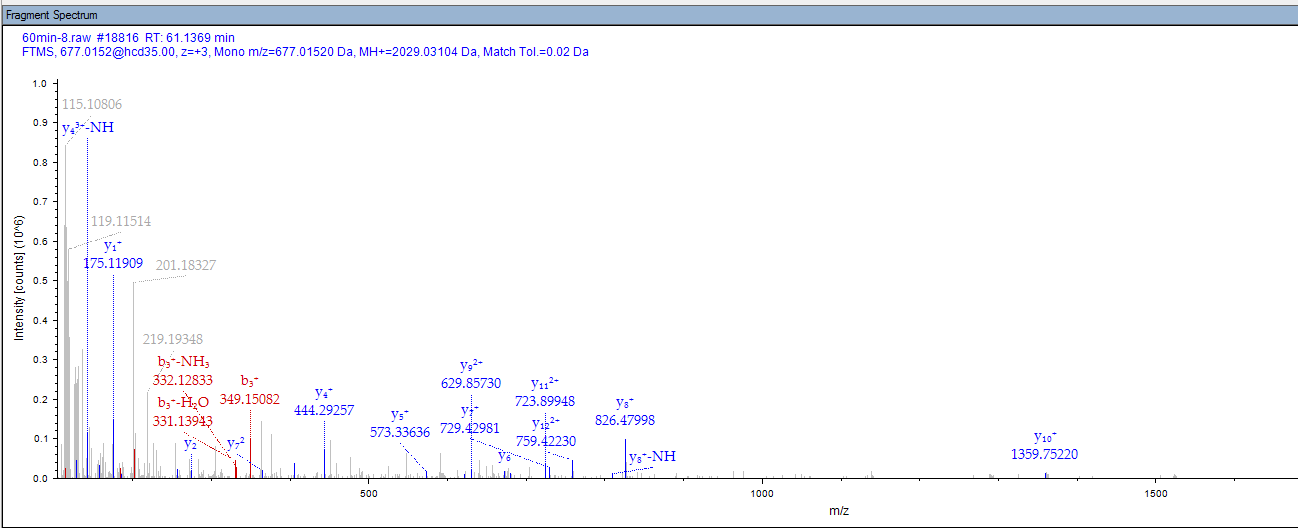


A0A0K9R8D4：AGQFCGGFTAIER


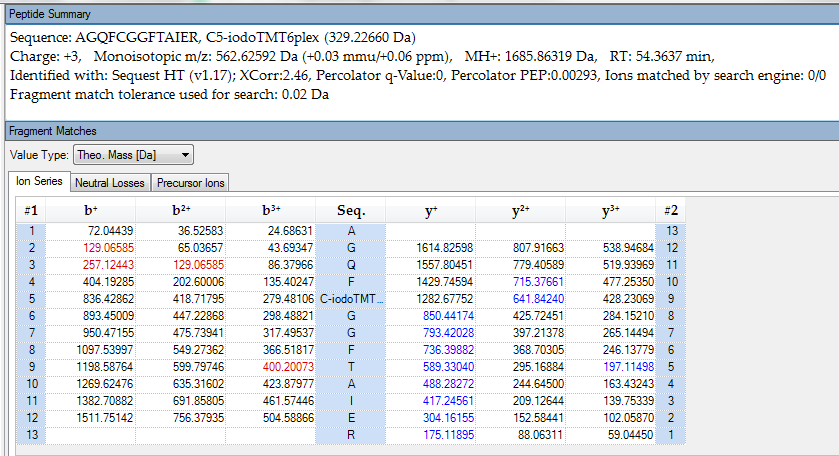

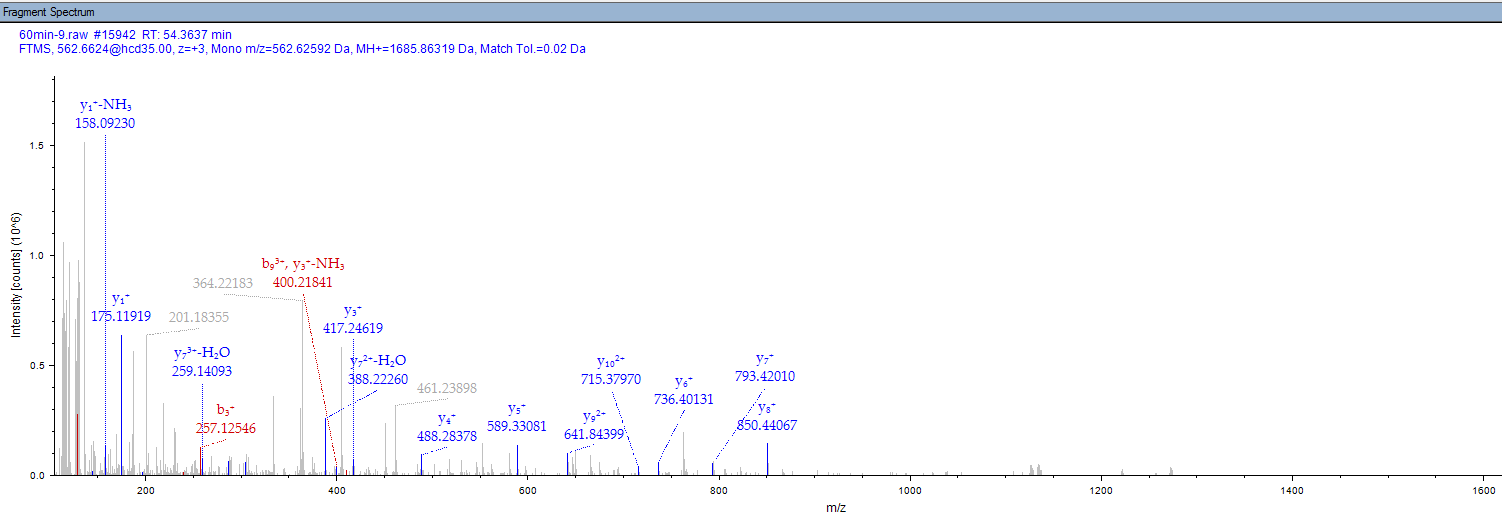


A0A068TKJ7：YTEGFSGADITEICQR


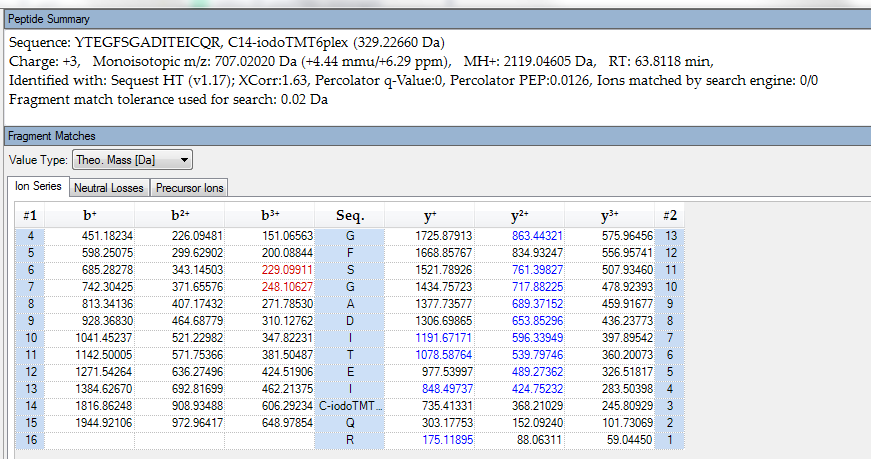

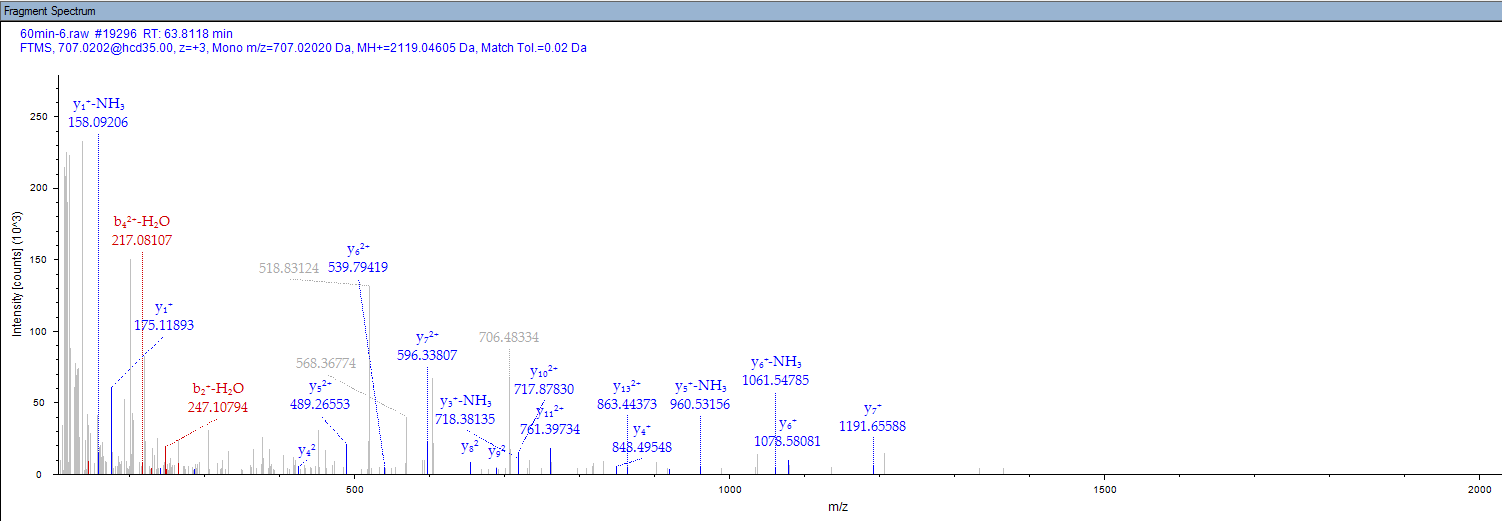


A0A0J8CV41：TLPEEILNSIIGETGVCPQAR


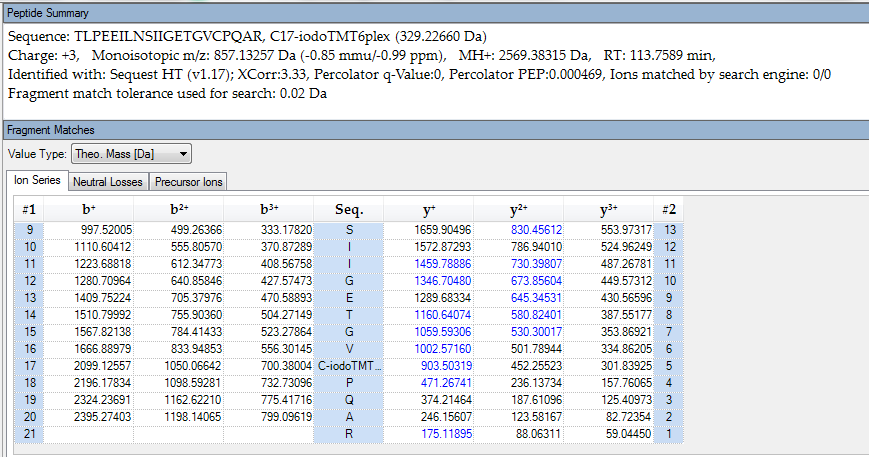

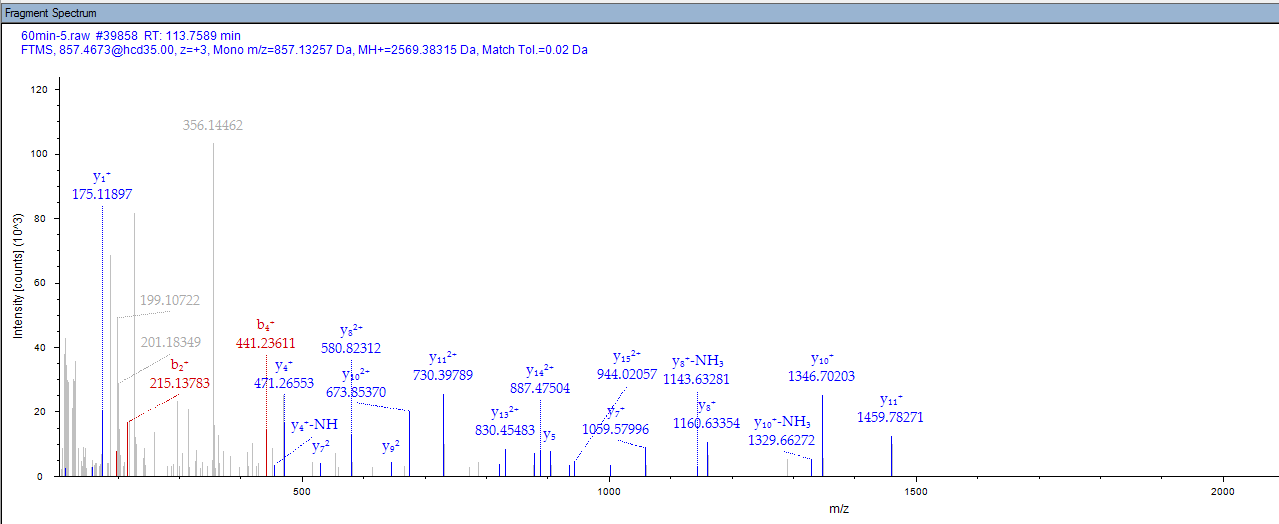


A0A0K9R8D4：AGQFCGGFTAIER


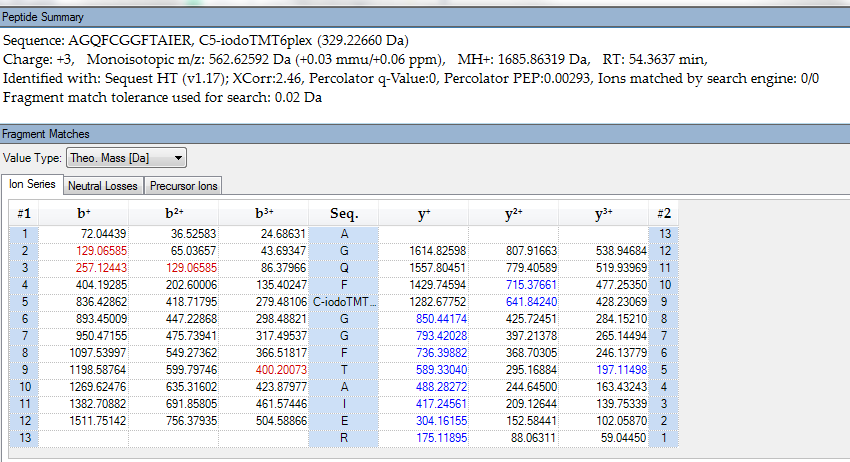

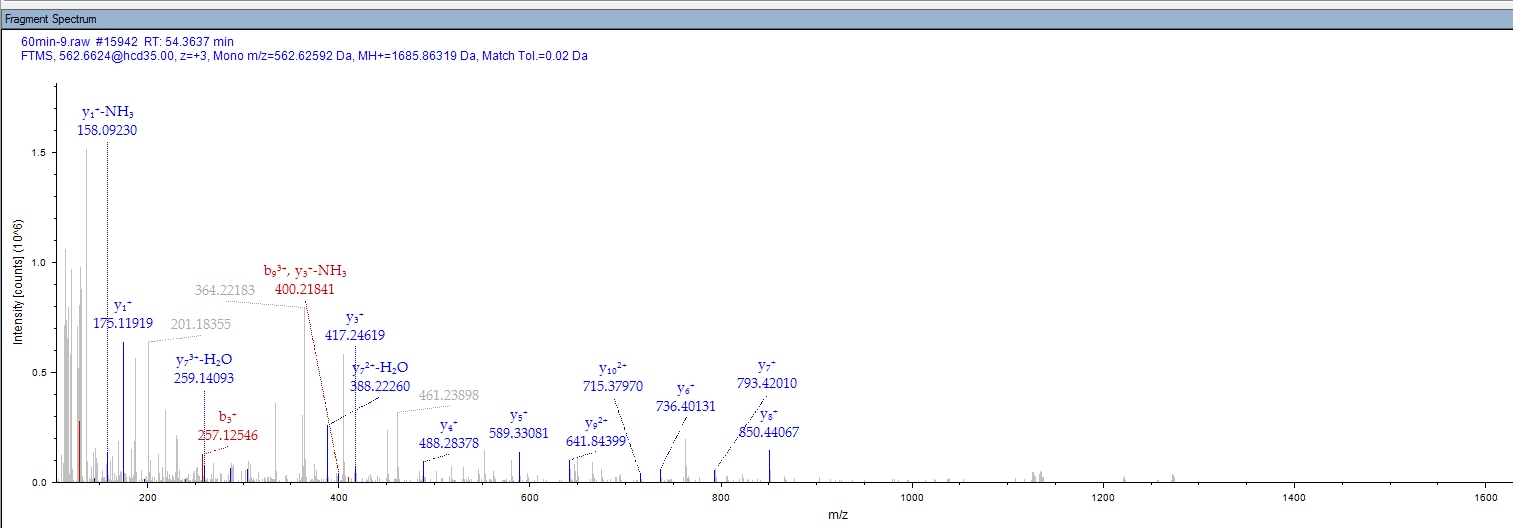


Q9M0C2：VTCVSGTNQGVPQPCR


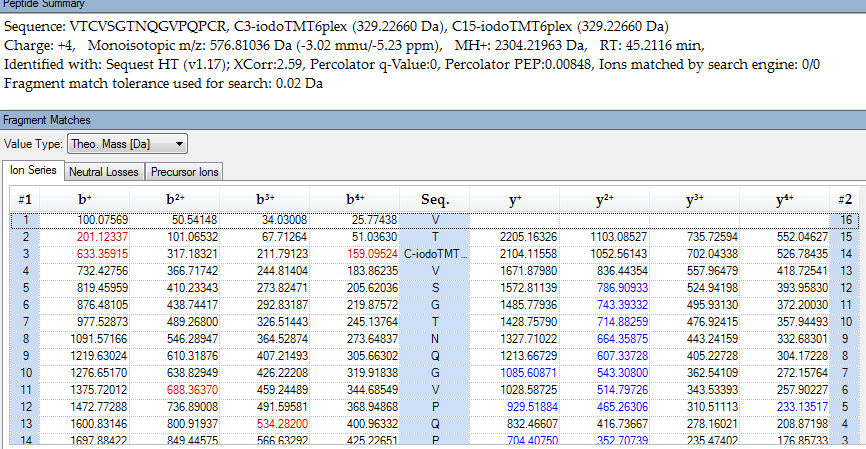

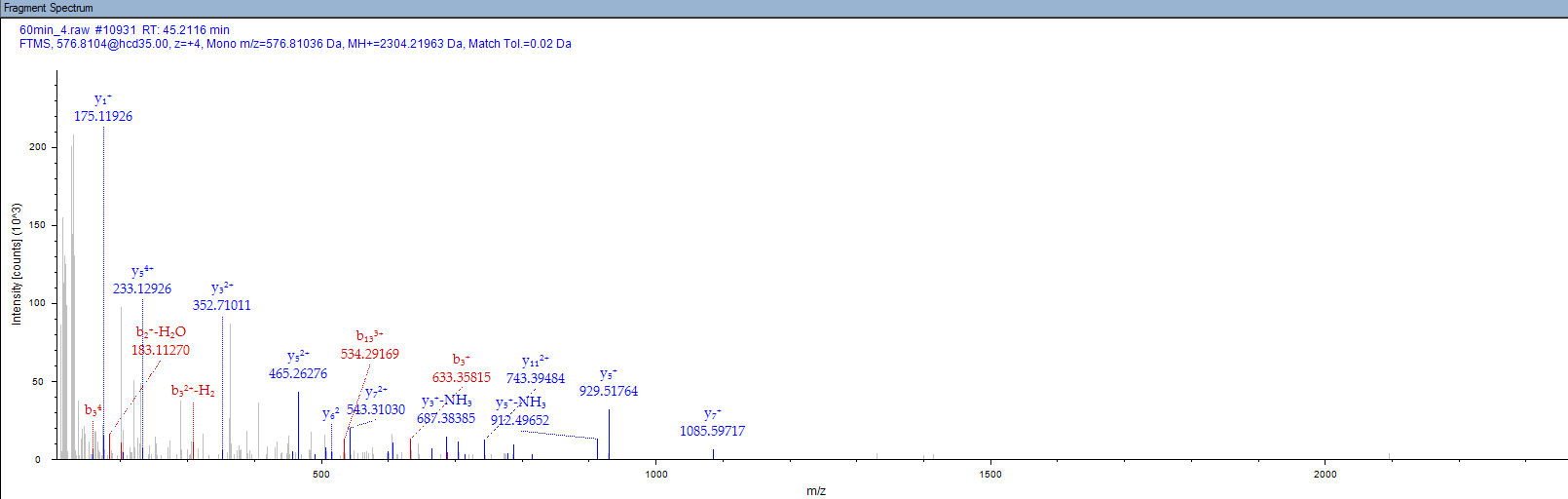


A0A0K9RNM7：CGVSIPGPVGPQADCSQIH


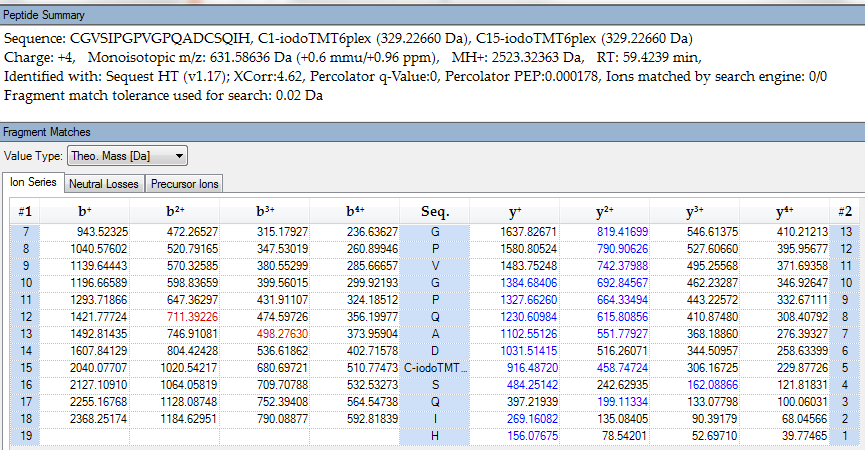

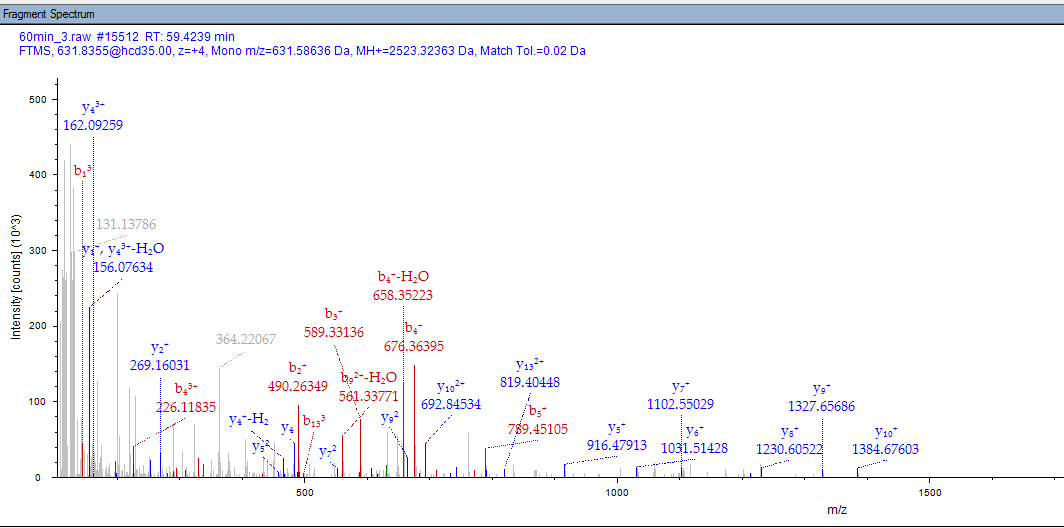


A0A0K9R8D4：AGQFCGGFTAIER


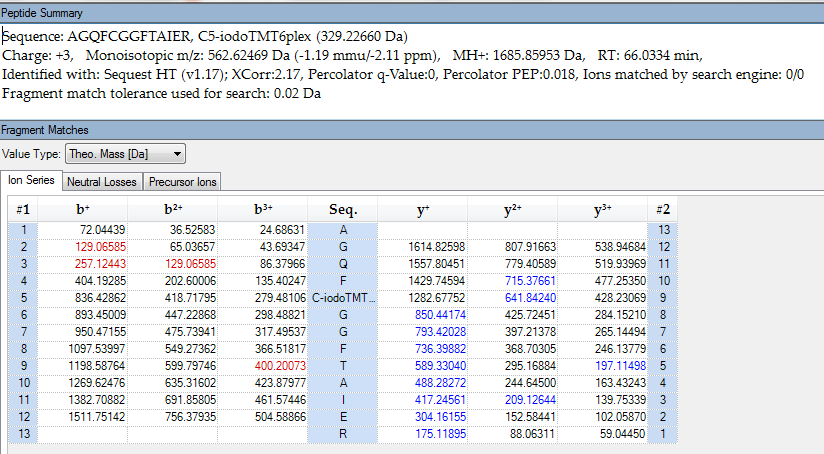

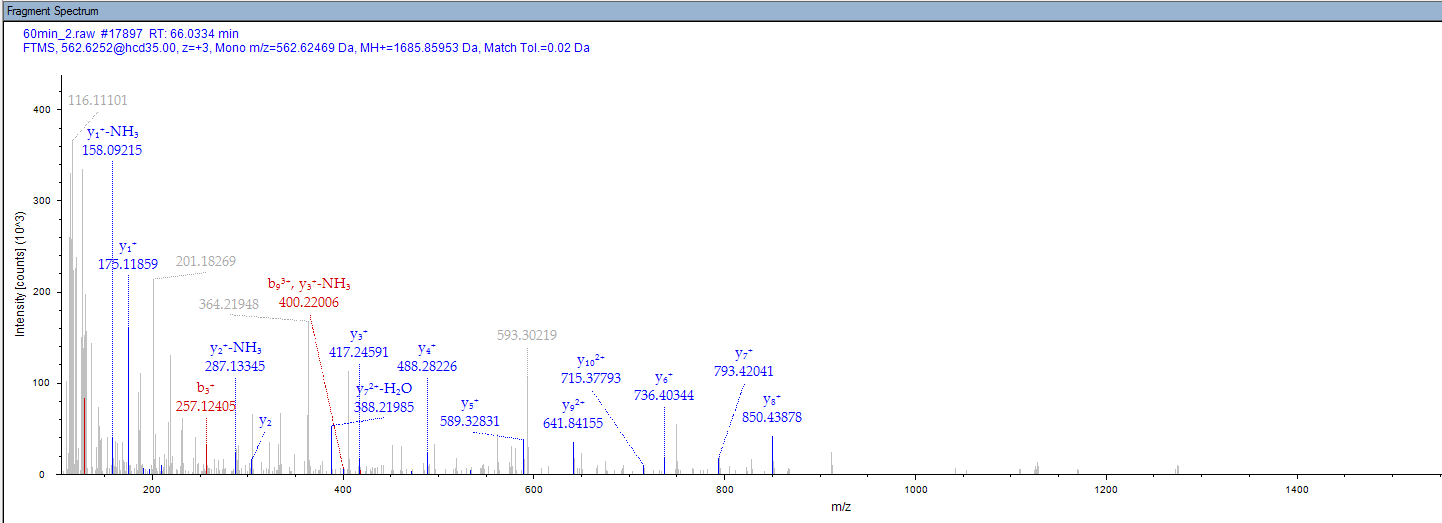


P10871：MCALFINDLDAGAGR


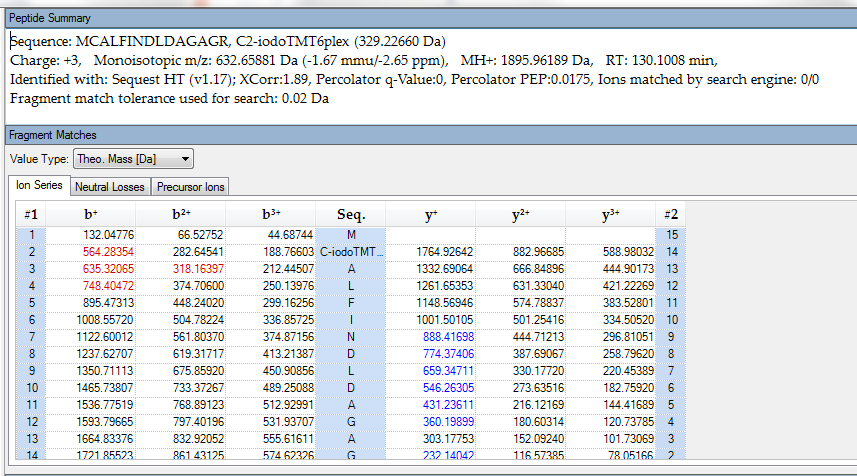

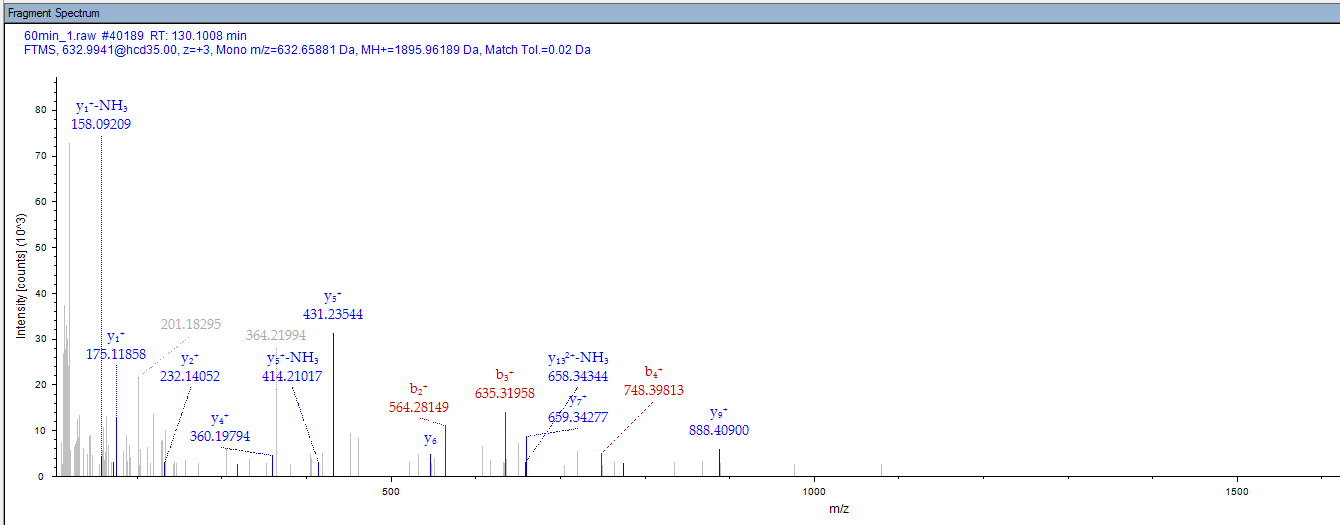

Supplement: Supplementary file 9 — Additional file 9: Table S7. MS/MS spectra showing redox modified cysteine sites. [file 40529_2021_320_MOESM9_ESM.docx]
